# The Genomes of the Fungal Plant Pathogens *Cladosporium fulvum* and *Dothistroma septosporum* Reveal Adaptation to Different Hosts and Lifestyles But Also Signatures of Common Ancestry

**DOI:** 10.1371/journal.pgen.1003088

**Published:** 2012-11-29

**Authors:** Pierre J. G. M. de Wit, Ate van der Burgt, Bilal Ökmen, Ioannis Stergiopoulos, Kamel A. Abd-Elsalam, Andrea L. Aerts, Ali H. Bahkali, Henriek G. Beenen, Pranav Chettri, Murray P. Cox, Erwin Datema, Ronald P. de Vries, Braham Dhillon, Austen R. Ganley, Scott A. Griffiths, Yanan Guo, Richard C. Hamelin, Bernard Henrissat, M. Shahjahan Kabir, Mansoor Karimi Jashni, Gert Kema, Sylvia Klaubauf, Alla Lapidus, Anthony Levasseur, Erika Lindquist, Rahim Mehrabi, Robin A. Ohm, Timothy J. Owen, Asaf Salamov, Arne Schwelm, Elio Schijlen, Hui Sun, Harrold A. van den Burg, Roeland C. H. J. van Ham, Shuguang Zhang, Stephen B. Goodwin, Igor V. Grigoriev, Jérôme Collemare, Rosie E. Bradshaw

**Affiliations:** 1 Laboratory of Phytopathology, Wageningen University, Wageningen, The Netherlands; 2 Centre for Biosystems Genomics, Wageningen, The Netherlands; 3 Laboratory of Bioinformatics, Wageningen University, Wageningen, The Netherlands; 4 Department of Plant Pathology, University of California Davis, Davis, California, United States of America; 5 Agricultural Research Center, Plant Pathology Research Institute, Giza, Egypt; 6 U.S. Department of Energy Joint Genome Institute, Walnut Creek, California, United States of America; 7 King Saud University, Riyadh, Saudi Arabia; 8 Institute of Molecular BioSciences, Massey University, Palmerston North, New Zealand; 9 Keygene N.V., Wageningen, The Netherlands; 10 Applied Bioinformatics, Plant Research International, Wageningen, The Netherlands; 11 CBS–KNAW Fungal Biodiversity Centre, Utrecht, The Netherlands; 12 Department of Forest Sciences, University of British Columbia, Vancouver, Canada; 13 Institute of Natural Sciences, Massey University, Albany, New Zealand; 14 CNRS and Aix-Marseille Université, Marseille, France; 15 Department of Plant Pathology, Tarbiat Modares University, Tehran, Iran; 16 Department of Bio-Interactions, Plant Research International, Wageningen, The Netherlands; 17 Cancer Genome Institute, Fox Chase Cancer Center, Philadelphia, Pennsylvania, United States of America; 18 INRA, Aix-Marseille Université, Marseille, France; 19 Department of Wheat Breeding, Seed and Plant Improvement Institute, Karaj, Iran; 20 University of Northern British Columbia, Prince George, Canada; 21 Department of Plant Biology and Forest Genetics, Swedish University of Agricultural Sciences, Uppsala, Sweden; 22 Department of Bioscience, Plant Research International, Wageningen, The Netherlands; 23 Swammerdam Institute for Life Sciences, University of Amsterdam, Amsterdam, The Netherlands; 24 Industrial Research Limited, Lower Hutt, New Zealand; 25 USDA–ARS/Department of Botany and Plant Pathology, Purdue University, West Lafayette, Indiana, United States of America; Stanford University School of Medicine, United States of America

## Abstract

We sequenced and compared the genomes of the Dothideomycete fungal plant pathogens *Cladosporium fulvum (Cfu)* (syn. *Passalora fulva*) and *Dothistroma septosporum (Dse)* that are closely related phylogenetically, but have different lifestyles and hosts. Although both fungi grow extracellularly in close contact with host mesophyll cells, *Cfu* is a biotroph infecting tomato, while *Dse* is a hemibiotroph infecting pine. The genomes of these fungi have a similar set of genes (70% of gene content in both genomes are homologs), but differ significantly in size (*Cfu* >61.1-Mb; *Dse* 31.2-Mb), which is mainly due to the difference in repeat content (47.2% in *Cfu* versus 3.2% in *Dse*). Recent adaptation to different lifestyles and hosts is suggested by diverged sets of genes. *Cfu* contains an α-tomatinase gene that we predict might be required for detoxification of tomatine, while this gene is absent in *Dse*. Many genes encoding secreted proteins are unique to each species and the repeat-rich areas in *Cfu* are enriched for these species-specific genes. In contrast, conserved genes suggest common host ancestry. Homologs of *Cfu* effector genes, including *Ecp2* and *Avr4*, are present in *Dse* and induce a Cf-Ecp2- and Cf-4-mediated hypersensitive response, respectively. Strikingly, genes involved in production of the toxin dothistromin, a likely virulence factor for *Dse*, are conserved in *Cfu*, but their expression differs markedly with essentially no expression by *Cfu in planta*. Likewise, *Cfu* has a carbohydrate-degrading enzyme catalog that is more similar to that of necrotrophs or hemibiotrophs and a larger pectinolytic gene arsenal than *Dse*, but many of these genes are not expressed *in planta* or are pseudogenized. Overall, comparison of their genomes suggests that these closely related plant pathogens had a common ancestral host but since adapted to different hosts and lifestyles by a combination of differentiated gene content, pseudogenization, and gene regulation.

## Author Summary

We compared the genomes of two closely related pathogens with very different lifestyles and hosts: *C. fulvum* (*Cfu*), a biotroph of tomato, and *D. septosporum* (*Dse*), a hemibiotroph of pine. Some differences in gene content were identified that can be directly related to their different hosts, such as the presence of a gene involved in degradation of a tomato saponin only in *Cfu*. However, in general the two species share a surprisingly large proportion of genes. *Dse* has functional homologs of *Cfu* effector genes, while *Cfu* has genes for biosynthesis of dothistromin, a toxin probably associated with virulence in *Dse*. *Cfu* also has an unexpectedly large content of genes for biosynthesis of other secondary metabolites and degradation of plant cell walls compared to *Dse*, contrasting with its host preference and lifestyle. However, many of these genes were not expressed *in planta* or were pseudogenized. These results suggest that evolving species may retain genetic signatures of the host and lifestyle preferences of their ancestor and that evolution of new genes, gene regulation, and pseudogenization are important factors in adaptation.

## Introduction

*Cladosporium fulvum* and *Dothistroma septosporum* are two related fungal species belonging to the class Dothideomycetes. *C. fulvum* is a biotrophic pathogen of tomato that has served as a model system for plant-microbe interactions since its first effector gene, *Avr9*, was cloned in 1991 [1]. It is not related to species in the genus *Cladosporium sensu strictu*, and has recently been renamed *Passalora fulva* [2]. However, to be consistent with past literature it will be referred to here as *C. fulvum*. Phylogenetic analyses based on sequences of the internal transcribed spacer (ITS) region of the ribosomal DNA revealed that *C. fulvum* is closely related to *D. septosporum* and other Dothideomycete fungi such as species of *Mycosphaerella* isolated from eucalyptus [3]. *D. septosporum* is an economically important hemibiotrophic pathogen of pine species that is well known for its production of an aflatoxin-like toxin, dothistromin [4]. A taxonomic revision also occurred for this species: prior to 2004 the name *Dothistroma pini* (syn. *D. septosporum* syn. *D. septospora*) was widely used. The revision involved a split into two species: the best-studied and most widespread species was named *D. septosporum*, and a less common species retained the name of *D. pini* [5].

The disease caused by *C. fulvum*, leaf mold of tomato, likely originates from South America, the center of origin of tomato [6]. The first outbreak of the disease was reported in South Carolina, USA, in the late 1800s [7]. Since then, disease outbreaks have occurred worldwide in moderate temperature zones with high relative humidity. The disease was of high economic importance during the first half of the 20^th^ century, but its importance waned after introgression of *Cf* (for *C* *.* *f* *ulvum*) resistance genes by breeders into tomato cultivars began providing effective control [8]. However, recent outbreaks have been reported in countries where tomato cultivars lacking *Cf* resistance genes are grown, and in areas where intensive year-round cultivation of resistant tomato plants led to fungal strains overcoming *Cf* genes [9], [10].

In contrast, the foliar forest pathogen *D. septosporum* (Dorog.) Morelet has a relatively recent history and has been less intensively studied than *C. fulvum*. *D. septosporum* infects over 70 species of pine, as well as several minor hosts including some *Picea* species [11]. During the 1960s–1980s, Dothistroma needle blight (DNB) was largely a problem of Southern hemisphere pine plantations, where primary control was achieved by fungicide applications or planting of resistant species (reviewed in [12]). Since the early 1990s DNB incidence has increased greatly in the Northern hemisphere, with some epidemics causing unprecedented levels of mortality [13], [14]. In northwest British Columbia, disease outbreaks are correlated with summer rainfall levels, suggesting that climate change could have unpredictable and severe effects on DNB outbreaks in forests [15].

**Figure 1:**
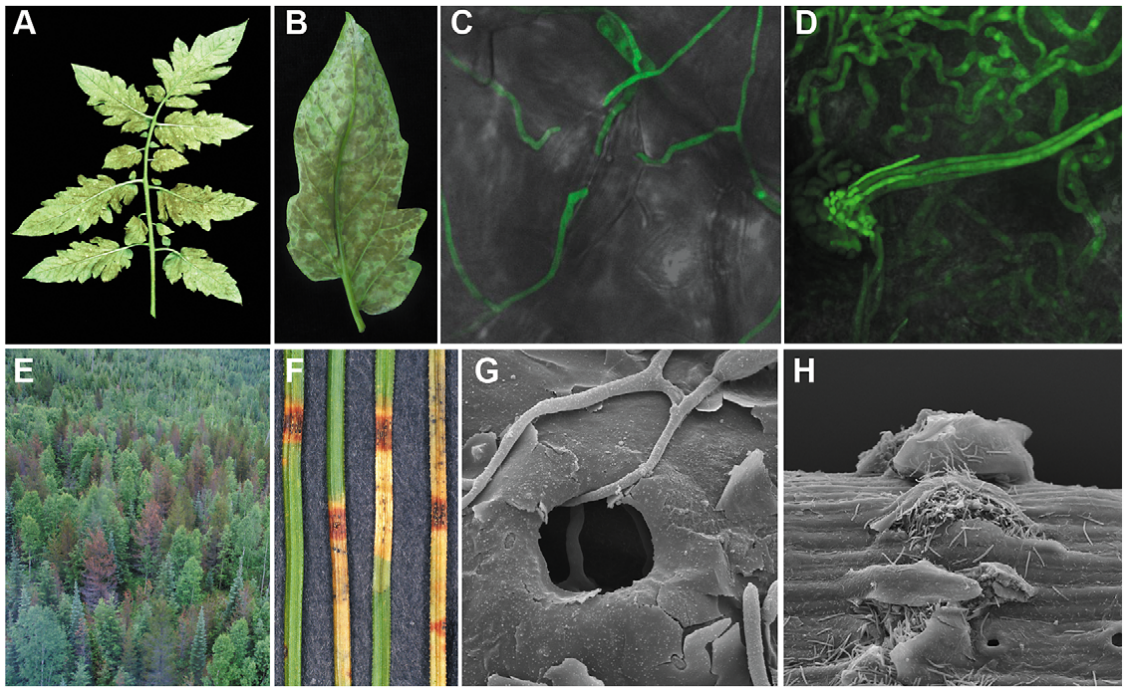
Symptoms caused by *Cladosporium fulvum* and *Dothistroma septosporum* on their host plants. (A–D) Disease symptoms of *Cladosporium fulvum* on tomato. A) *C. fulvum* sporulating on the lower side of a tomato (*Solanum lycopersicum*) leaf two weeks post inoculation; B) Close-up of *C. fulvum* sporulating on the lower side of a single leaflet two weeks post inoculation; C) Runner hyphae of *C. fulvum* (GFP-transgenic strain) at the surface of the leaf; two of them are penetrating a stoma of tomato four days post inoculation; D) Conidiophores of *C. fulvum* (GFP-transgenic strain) emerging from a stoma at 10 days post inoculation. (E–H) Disease symptoms of *Dothistroma septosporum* on pine. E) Mortality of mature lodgepole pines (*Pinus contorta* var. *latifolia*) in northwest British Columbia, Canada caused by *D. septosporum*; F) Red band lesions with conidiomata on *Pinus radiata* needles; G) Penetration of hypha into stoma of *P. radiata* needle 4 weeks post inoculation; H) Eruption of conidiospores through epidermis of pine needle 8 weeks post inoculation.

Infection in both the *C. fulvum*-tomato and *D. septosporum*-pine pathosystems starts with conidia that germinate on the leaf surface and produce runner hyphae that enter the host through open stomata. Subsequently, the fungi colonize the apoplastic space between mesophyll cells. In the case of *C. fulvum*, conidiophores emerge from stomata 10–14 days later producing massive amounts of conidia that can re-infect tomato [16], [17], [18] (Figure 1A–1D). *D. septosporum* produces conidia, several weeks after infection, on conidiomata that erupt through the needle epidermis where they can be spread to other pines by rain splash [19], [20] (Figure 1E–1H). Whilst *C. fulvum* is considered a biotroph, *D. septosporum* is assumed to be a hemibiotroph based on similarities of its lifecycle to other Dothideomycete fungi.

There is no evidence that *C. fulvum* has an active sexual cycle, although both mating type idiomorphs occur in its global population [21]. Although *D. septosporum* also has a predominantly asexual lifestyle, it is known to be sexually active in some parts of the world. The sexual stage *Mycosphaerella pini* Rostr. (syn *Scirrhia pini* Funk & Parker) has been reported in some forests in Europe and North America but has not yet been found in other regions, such as South Africa or the United Kingdom, even though both mating types are known to be present [22]. The rare sightings of the sexual stage are due partly to difficulties in identification, but also reflect findings from population studies that show mixed modes of reproduction with a significant clonal component [23], [24]. So far, attempts to induce a sexual cycle between opposite mating types of *D. septosporum* in culture in our laboratory or others (Brown A, unpublished data) have failed. Further research is required to determine environmental conditions conducive to sexual reproduction. The *D. septosporum* isolate whose genome was sequenced is derived from a clonal population with a single mating type that was introduced into New Zealand in the 1960s [22], [25].

The *C. fulvum*-tomato interaction complies with the gene-for-gene model [2], [26]. During infection *C. fulvum* secretes effector proteins into the apoplast of tomato leaves which function not only as virulence factors, but also as avirulence (Avr) factors when recognized by corresponding tomato Cf resistance proteins. This recognition leads to Cf-mediated resistance that often involves a hypersensitive response (HR) preventing further ingress of the fungus into its host plant tomato [8]. To date many cysteine-rich effectors have been cloned from *C. fulvum*, including Avr2, Avr4, Avr4E and Avr9, that can trigger Cf-2-, Cf-4-, Cf-4E-, and Cf-9-mediated resistance, respectively, and Ecps (*e*xtra*c*ellular *p*roteins) like Ecp1, Ecp2, Ecp4, Ecp5 and Ecp6 that trigger Cf-Ecp-mediated resistance [27], [28], [29]. Specific functions for some *C. fulvum* effectors have been determined: Avr4 is a chitin-binding protein that protects fungi against the deleterious effects of plant chitinases [30], [31], Ecp2 is a virulence factor that occurs in many fungi [32], [33] and Ecp6 sequesters chitin fragments released from fungal cell walls by chitinases during infection thereby dampening their potential to induce pathogen-associated molecular pattern (PAMP)-triggered immunity [28]. Initially, the Avr and Ecp effectors seemed unique to *C. fulvum*, but in recent years homologs of Avr4, Ecp2 and Ecp6 with functions in virulence have been found in other fungal genomes, including members of the Dothideomycetes [27], [28], [33].

Whilst most studies of *C. fulvum* have focused on effectors and their interactions with components in both resistant and susceptible plants, studies of *D. septosporum* have instead focused on dothistromin, a toxin produced by the fungus that accumulates in infected pine needles. Dothistromin is a broad-spectrum toxin with structural resemblance to a precursor of the highly toxic and carcinogenic fungal metabolite, aflatoxin [34]. Although dothistromin is not essential for pathogenicity [35], recent observations suggest it to be a virulence factor, affecting lesion size and spore production (Kabir MS and Bradshaw RE, unpublished data). Some dothistromin biosynthetic genes were identified in *D. septosporum* but unexpectedly they were in several mini-clusters rather than in one co-regulated cluster of genes as reported for aflatoxin-producing species of *Aspergillus* [36], [37], [38]. The similarity of dothistromin to aflatoxin enabled predictions to be made about other *D. septosporum* genes involved in dothistromin production [39]; the complete set of dothistromin genes will help us understand the evolution of dothistromin and aflatoxin gene clusters.

Here we report the sequence and comparison of the genomes of *C. fulvum* and *D. septosporum*, which have very similar gene contents but differ significantly in genome size as a result of different repeat contents. We found unexpectedly high levels of similarity in genes previously studied in one or other of these fungi, including those encoding Avr and Ecp effectors of *C. fulvum*, and dothistromin toxin genes of *D. septosporum*. Surprisingly, compared to *D. septosporum*, *C. fulvum* has higher numbers of genes normally associated with a necrotrophic or hemibiotrophic lifestyle such as genes for carbohydrate-degrading enzymes and secondary metabolite biosynthesis. However, in *C. fulvum* some of these genes were lowly or not expressed *in planta* and others were pseudogenized. Other *C. fulvum* genes that are absent in *D. septosporum* are putatively involved in virulence on its host plant tomato, such as the α-tomatinase gene. We suggest that regulation of gene expression and pseudogenization, in addition to evolution of new genes, are important traits associated with adaptation to different hosts and lifestyles of the two fungi that, however, also retained some signatures of their common ancestral host.

## Results/Discussion

### *C. fulvum* and *D. septosporum* are closely related species with very different genome sizes

**Figure 2:**
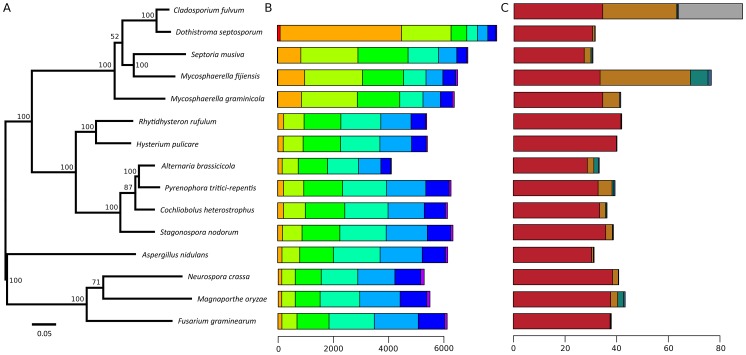
Species phylogeny, amino acid similarity, and repeat content. A) Maximum likelihood phylogenetic tree based on 51 conserved protein families showing evolutionary relationships of *Cladosporium fulvum* and *Dothistroma septosporum*. Branch lengths are indicated by the bar (substitutions/site); bootstrap values are shown as percentage. B) Genome-wide amino acid similarity of homologous proteins between *C. fulvum* and other sequenced fungal species. A pair of proteins is only reported as homologous when the predicted similarity (blastp) spans at least 70% of their lengths and their length difference is at most 20%. Axis indicates number of homologous proteins. Bar shading indicates similarity: red, 91–100%; orange, 81–90%; light green, 71–80%; medium green, 61–70%; turquoise, 51–60%; light blue, 41–50%; dark blue, 31–40%; and purple, 0–30%. Homologous proteins with high amino acid similarity are likely orthologs, whereas for those with lower similarity this relation cannot be inferred. C) Repeat content of *C. fulvum*, *D. septosporum* and other sequenced fungal species. Bar shading indicates repeat class: red, unique non-repeat regions; brown, repeat elements; green, continuous tracts of N characters; blue, duplicated regions; and grey, poorly assembled regions (*C. fulvum* only). Axis indicates number of nucleotides (Mb).

The 30.2-Mb genome of *D. septosporum* (http://genome.jgi.doe.gov/Dotse1/Dotse1.home.html; GenBank AIEN00000000) was sequenced at 34-fold coverage (Table S1) and then assembled into 20 scaffolds (>2-kb), 14 of which were 407-kb or larger, have telomere sequences at one or both ends (Table S2) and mostly match chromosome sizes estimated from pulsed-field gel electrophoresis [36]. The six smallest scaffolds ranged from 2.3- to 5.2-kb in size so are not significant parts of the genome. The excellent assembly of the *D. septosporum* genome was facilitated by its very low repeat content of only 3.2% (Table 1; Table 2; Protocol S1). In contrast, the repeat-rich genome of *C. fulvum* (http://genome.jgi-psf.org/Clafu1/Clafu1.home.html; GenBank number AMRR00000000) was very difficult to assemble. Fourteen 2-kb paired-end or shotgun 454 sequencing runs for *C. fulvum* resulted in a 21-fold coverage of the 61.1-Mb assembly in 2664 scaffolds >2-kb (Table 1) with a total repeat content of 47.2% (Table 2). The sequencing strategy was initially based on the assumption of a genome size of around 40-Mb, but soon it appeared that the *C. fulvum* genome was much larger due to the high repeat content. Problems with the assembly are not caused by the sequencing coverage of *C. fulvum* because it is estimated to be sufficiently high for good coverage of the gene-encoding areas. Instead, they are a consequence of its high repeat content. An estimated additional 26-Mb of *C. fulvum* DNA reads could not be assembled as they were predominantly repeat sequences (Figure 2). In the remainder of the manuscript we refer to chromosomes (1 to 14) for *D. septosporum* and scaffolds for *C. fulvum*. Summary statistics for the two genomes are shown in Table 1 and at the Joint Genome Institute (JGI) Genome portal (jgi.doe.gov/fungi) [40]. The *C. fulvum* and *D. septosporum* genomes are predicted to encode approximately 14 and 12.5 thousand gene models, respectively. Nevertheless, the *C. fulvum* and *D. septosporum* genomes share more than 6,000 homologous gene models with at least 80% similarity at the predicted amino acid level, whereas this number drops to 3,000 gene models this similar when comparing *C. fulvum* or *D. septosporum* with other closely related Dothideomycete species such as *Mycosphaerella graminicola* and *M. fijiensis* (Figure 2, Figure S1). Similarly, most introner-like element clusters found in *C. fulvum* and *D. septosporum* are closely related, more than to elements in other Dothideomycetes [41].

**Table 1:** *C. fulvum* and *D. septosporum* genome statistics.

Species	Genome assemblysize (Mb)	Number of predicted gene models	Sequencing coverage depth	Number of scaffolds^a^	Scaffold L50/N50^b^ (Mb)	% GC content
*C. fulvum*	61.1	14,127	21	2,664>2 kb 279>50 kb	250/0.06	48.8
*D. septosporum*	30.2	12,580	34	20>2 kb 14>50 kb	5.0/2.6	53.1

a For details see Protocol S1.

b L50 is defined as the smallest number of scaffolds that make up 50% of the genome; N50 is defined as the size (Mb) of the smallest of the L50 scaffolds.

**Table 2:** Repetitive elements in the *C. fulvum* and *D. septosporum* genomes.

		*C. fulvum*			*D. septosporum*		
Repeat class	Repeat type	Total bases covered	Percent of genome^a^	Percent of repetitive fraction^a^	Total bases covered	Percent of genome	Percent of repetitive fraction
Class I	Ty1-Copia LTR	3,208,305	5.3	11.1	43,598	0.1	4.5
Retrotransposons	Ty3-Gypsy LTR	13,557,457	22.2	47.0	178,860	0.6	18.6
	Misc. LTR	0	0	0	167,893	0.6	17.5
	LINE	9,464,476	15.5	32.8	0	0	0
	**Sub-total**	**26,230,238**	**42.9**	**90.9**	**390,351**	**1.3**	**40.60**
Class II	DDE-1	960,373	1.6	3.3	0	0	0
DNA transposons	hAT	144,765	0.2	0.5	0	0	0
	Helitron	14,901	0.02	0.1	384,233	1.3	40.0
	Mariner	96,124	0.2	0.3	57,646	0.2	6.0
	MITE	98,047	0.2	0.3	2,165	0.01	0.2
	MuDR_A_B	50,899	0.1	0.2	0	0	0
	**Sub-total**	**1,365,109**	**2.2**	**4.7**	**444,044**	**1.5**	**46.2**
Unclassified	Unclassified	1,255,529	2.1	4.4	127,027	0.4	13.2
**TOTAL**		**28,850,876**	**47.2**		**961,422**	**3.2**	

a Numbers refer to repeats present in assembled genome.

Phylogenetic analysis of *C. fulvum* and *D. septosporum* genomes in the context of nine other Dothideomycetes [42] confirms that these two species are the most closely related of the sampled species (Figure 2), as was inferred earlier from ITS [3] and mating type sequences [21]. This gives us two very closely related genomes with drastically different genome sizes mostly due to the greatly increased repeat content of *C. fulvum*.

### *C. fulvum* and *D. septosporum* differ in content and classes of repeats that are affected by repeat-induced point mutation

The massive increase in repetitive elements in *C. fulvum* might result from expansion of one or more repeat families that are also present in *D. septosporum*. Therefore, we classified the different repeat families in *D. septosporum* and compared them with those in *C. fulvum*. This revealed that some of the repetitive element families present in *D. septosporum* have expanded in *C. fulvum* (Table 2). This is most remarkable for the Class I retrotransposons which comprise over 90% of the repetitive fraction in *C. fulvum* and together account for over 26-Mb of the assembled genome. Retrotransposons are also highly abundant in the large repeat-rich genome of the hemibiotrophic sexual pathogen *Mycosphaerella fijiensis* (Dhillon B, Goodwin SB and Kema GHJ, unpublished data). Both Copia and Gypsy LTR retroelements are expanded in *C. fulvum* compared to the *D. septosporum* genome, whereas LINEs are detected only in *C. fulvum* (Table 2). Some other fungal species that are closely related to each other, but have a different lifestyle, also differ in repeat content, such as the Leotiomycetes of which *Botrytis cinerea* (<1% repeats) and *Sclerotinia sclerotiorum* (7% repeats) are necrotrophs, while *Blumeria graminis* f. sp. *hordei* (64% repeats) is an obligate biotroph [43], [44]. The latter species is particularly enriched in Class I elements and one of several biotrophs that show expansion of genome size associated with high repeat content [44], [45].

**Figure 3:**
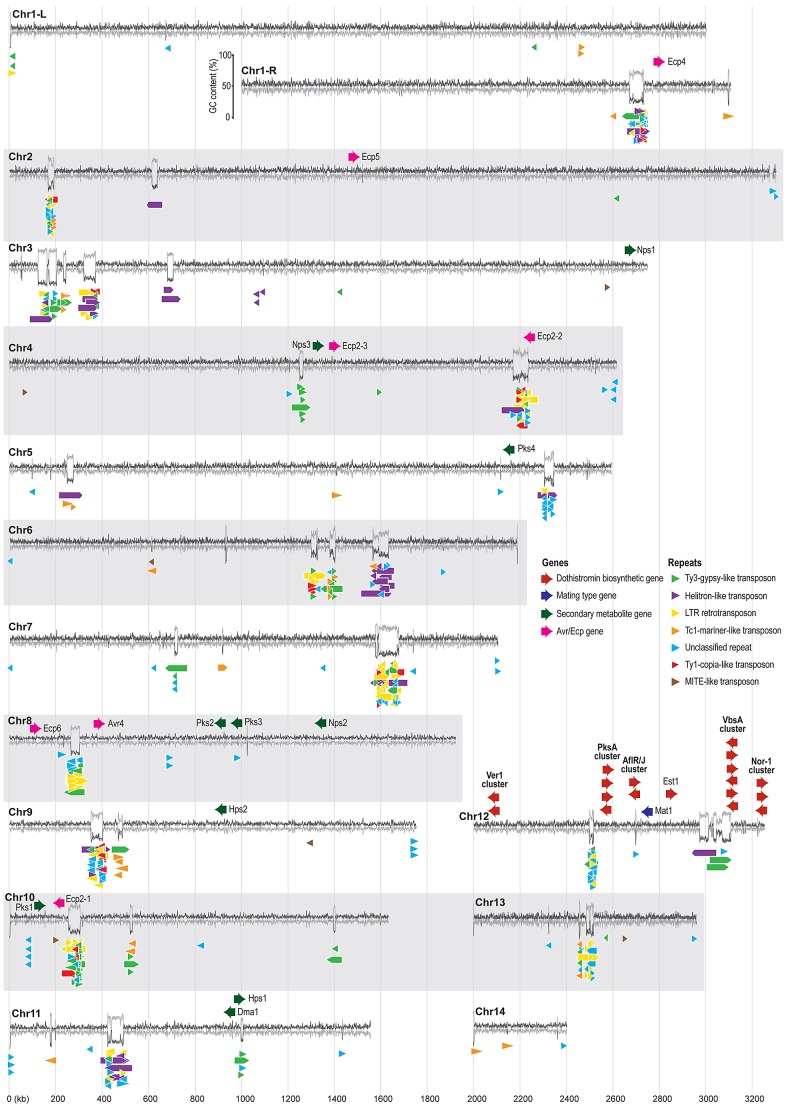
Organization of repeats and pathogenicity-related genes in the *Dothistroma septosporum* genome. The fourteen chromosomes from the *D. septosporum* genome assembly are shown as GC (dark grey line) and AT (pale grey line) content (%) plots made from a 500-bp sliding window using Geneious (www.geneious.com). All chromosomes have telomere sequence at both ends except chromosomes 2, 11 and 14 which have telomere sequences only at the left end as shown in the figure. Chromosome 1 has been split into two parts in the figure (L, R) because of its length, and the GC/AT content scale is shown beside the right arm of this chromosome. The positions of putative *Avr* and *Ecp* effector, secondary metabolite, dothistromin biosynthesis, and mating type genes are shown above the GC/AT content plot, while the positions of repeats (>200-bp) are shown below the plot. Color-coding of the gene and repeat types is indicated in the legend. Most chromosomes have repeat clusters at one or two sites that coincide with regions of high AT content. The chromosome sizes are to scale, as indicated by the vertical pale grey lines, with the values (in kb) shown at the bottom; neither the genes nor the repeats are drawn to scale.

In contrast to the retroelements, Class II DNA transposons comprise only a small percentage (4.7%) of the overall repetitive elements in *C. fulvum*, but 46.2% of the repeats in *D. septosporum*, although they make up only a small portion of the genome overall. Interestingly, helitron-like DNA transposons comprise 40% of all repeats in *D. septosporum* and are 25.8-fold higher in terms of sequence coverage than in *C. fulvum*, whereas the DDE-1, hAT, and MuDR_A_B DNA transposons present in *C. fulvum* are not present in *D. septosporum*. Helitrons are transposons that replicate by a rolling-circle mechanism and are found in a wide range of eukaryotes, including the white rot fungus *Phanerochaete chrysosporium* [46], and are thought to have a role in genome evolution [47]. Helitron-like repeats are particularly abundant on *D. septosporum* chromosomes 3, 6 and 11 (Figure 3) and usually occur in clusters, sometimes along with other types of repetitive elements.

The organization of repeats in *D. septosporum* is striking in that for the majority of the chromosomes, most repeats are localized into just one or two large regions containing a mixture of repeat element types (Figure 3), although other small repeat clusters also occur. In many eukaryotes, centromeres are characterized by repetitive DNA [48], and therefore we propose that some of the larger complex repeat regions are centromeres, in line with similar suggestions made for other fungal genomes [49], although experimental confirmation is required. The absence of any repeat cluster from chromosome 14, along with the observation that it harbors only one telomere, suggests that it is a chromosome fragment.

Repeats in fungi are affected by *r*epeat-*i*nduced *p*oint mutation, also referred to as RIP, a defense mechanism employed by fungi to suppress transposable element activity that was first described in *Neurospora crassa* [50]. RIP is a process by which DNA accumulates G:C to A:T transition mutations. It occurs during the sexual stage in haploid nuclei after fertilization but prior to meiotic DNA replication. Clear evidence of RIP was found in both the *C. fulvum* and *D. septosporum* genomes (Table 3) and is mainly confined to repeat-rich regions. In total 25.9-Mb were RIP'd in *C. fulvum* and 1.1-Mb in *D. septosporum*, which represent 42.4% and 3.7% of their genomes, respectively. RIP occurred mainly on large repeated sequences (≥500 nucleotides) that represent 97.2% of all repeats in *C. fulvum* and 98.0% in *D. septosporum* (Table 3). The high rate of RIP in repeat regions is in the same range as that seen in other Dothideomycetes such as *S. nodorum* (97.2%; Table S3) [42], [51]. Although RIP is present at high levels in *C. fulvum*, we propose that it has not been able to prevent transposon expansion possibly due to very rare sexual activity.

**Table 3:** Occurrence of Repeat-Induced Point Mutation (RIP) signatures and repeats in *C. fulvum* and *D. septosporum*.

	*Cladosporium fulvum*		*Dothistroma septosporum*	
	Bases (kb)	Loci^a^	Bases (kb)	Loci^a^
RIP'd sequence in genome	25,882 (42.4%)^b^	5447	1,114 (3.7%)^b^	65
Repeat sequence ≥500 nt in genome^c^	27,170 (44.5%)^b^	7101	798 (2.6%)^b^	133
Repeat sequence ≥500 nt with RIP signature^d^	26,397 (97.2%)^e^	6506 (91.6%)^e^	782 (98.0%)^e^	114 (85.7%)^e^

a Number of loci, defined as consecutive blocks of sequence assigned as repeats or RIP'd regions.

b Percentage of the genome is indicated in brackets.

c Only classified repeats (as shown in Table 2) were considered.

d Repeated sequence ≥500 nt that (at least partially) overlaps with RIP'd sequence.

e Percentage of classified repeats is indicated in brackets.

Of the RIP'd loci, *C. fulvum* has almost none (0.5%) and *D. septosporum* little (16.9%) outside the main classified repeat regions. This is different from *N. crassa* (Table S3), where 35.2% of all RIP'd loci are predicted to be non-repeat-associated. For *N. crassa* it has been shown that even single gene duplication events are prey to the RIP machinery, thereby exemplifying its efficiency and sensitivity [50]. Clearly such sensitivity is not applicable to *C. fulvum* and *D. septosporum*, nor for three other studied Dothideomycetes (Table S3). In the Dothideomycete phytopathogenic fungus *Leptosphaeria maculans*, RIP slippage is found in regions adjacent to repetitive elements. In that species RIP has occurred in genes encoding small secreted proteins, such as the effector genes *AvrLm6* [52] and *AvrLm1* [53] that are located in repeat-rich regions of the genome [54]; mutations in these genes caused by the RIP process enabled the fungus to overcome *Lm6* and *Lm1*-mediated resistance, respectively. However, we found no evidence of RIP slippage into the known effector genes of *C. fulvum* and related effector genes in *D. septosporum*.

### The *C. fulvum* and *D. septosporum* genomes show extensive intrachromosomal rearrangements

One way to assist the assembly of a fragmented genome is to use synteny with a well-assembled genome of a closely related species to order the scaffolds [55]. We attempted to use the *D. septosporum* genome to improve the *C. fulvum* assembly in this way. However, although it was possible to map *C. fulvum* scaffolds onto the assembled *D. septosporum* genome (Figure S2A), individual *C. fulvum* scaffolds are not collinear along their length, but have only short blocks of synteny to different parts of the *D. septosporum* chromosomes. The syntenic regions of the *C. fulvum* and *D. septosporum* genomes are associated with just 461 of the *C. fulvum* scaffolds (Table 4). In contrast, the remaining >4,000 *C. fulvum* scaffolds are non-syntenic. A more detailed analysis with the ten largest *C. fulvum* scaffolds (two are shown in Figure S2B) revealed that they each match primarily to only one *D. septosporum* chromosome, suggesting predominantly intrachromosomal rearrangements (mesosynteny), as described for other Dothideomycete fungi [42], [56] (Condon B et al., unpublished data). As found in other fungi [43], [57] non-syntenic regions are repeat-rich; for *C. fulvum* 79.7% of the repeat sequences are present in non-syntenic regions (Table 4).

**Table 4:** Syntenic and non-syntenic regions between *C. fulvum* and *D. septosporum* are unevenly distributed over the *C. fulvum* scaffolds.

Feature	Syntenic	Non-syntenic	Total	Syntenic %	Non-syntenic %
Number of scaffolds	461	4,404	4,865	9.5	90.5
Number of repeats	2,090^a^	6,234	8,324	25.1	74.9
Mb in scaffolds	37.4^b^	23.7	61.1	61.2	38.8
Mb in repeats	5.6^c^	21.9	27.5	20.3	79.7
Mb in whole genome	22.3^b^	38.8	61.1	36.5	63.5

a Number of repeat regions on syntenic vs. non-syntenic scaffolds.

b A syntenic scaffold is one that contains at least a single syntenic block, but may not be syntenic along its entire length. Total syntenic scaffold size (37.4-Mb) is therefore larger than total syntenic size in whole genome (22.3-Mb).

c Summed repeat length on syntenic *versus* non-syntenic scaffolds.

### Non-syntenic, repeat-rich regions are enriched in genes encoding secreted proteins

Secreted proteins are important for communication of plant-pathogenic fungi with their hosts. They comprise not only enzymes required for penetration and growth on plant cell walls, but also proteins needed to compromize the basal defence system of plants by either suppressing or attacking it, as has been reported for several fungal effector proteins [58]. The percentage of proteins predicted to be secreted is similar for both *C. fulvum* (8.5%) and *D. septosporum* (7.2%), and in the same range as that predicted for other Dothideomycete fungi such as *M. graminicola* (9.1%) and *S. nodorum* (10.8%) [40], [42], [51], [59].

Genes encoding secreted proteins including effectors are subject to evolutionary selection pressure imposed by environmental and host plant factors [58], and they often show a high level of diversification. Repeat-rich, gene-poor regions have been proposed to contain genes involved in adaptation to new host plants. For example, in some *Phytophthora* species and in *L. maculans* significantly higher proportions of *in planta*-induced species-specific effector genes encoding secreted proteins are found in repeat-rich compared to repeat-poor regions [50], [59] and in pathogenic strains of *Pyrenophora tritici-repentis* transposable elements are associated with effector diversification (Manning V, Ciuffetti L, unpublished data). We hypothesized that we would find more genes encoding secreted proteins in repeat-rich regions that are less syntenic between the *C. fulvum* and *D. septosporum* genomes than in repeat-poor syntenic regions. We therefore compared the number of genes and their similarity at the nucleotide and protein levels in syntenic and non-syntenic regions of these two genomes (Table 5) using *C. fulvum* as the reference sequence due to its higher overall content of repeat elements in non-syntenic regions. The regions syntenic between *C. fulvum* and *D. septosporum*, representing 22.3-Mb of the *C. fulvum* genome, contain 70% of all predicted genes whereas 30% of the genes are located in the non-syntenic repeat-rich regions representing 38.8-Mb of the *C. fulvum* genome (Table 5). The syntenic regions contain most of the homologous genes that encode proteins with the highest level of conservation between the two genomes, whereas the proteins encoded by genes located in the non-syntenic, repeat-rich regions are less conserved. In syntenic regions, 89.9% of gene models have a bi-directional best BLAST hit (BDBH) to a *D. septosporum* gene model, with a mean predicted amino acid similarity of 85.2%, compared to non-syntenic with only 51.7% of gene models with BDBH and 65.1% amino acid similarity (Table 5). As expected, we found the repeat-rich, non-syntenic regions to have higher proportions of gene models encoding secreted proteins (10.4%, with a mean predicted amino acid similarity of 60.7%) and small secreted cysteine-rich proteins (2.8%) than in syntenic regions (7.6%, with a mean amino acid similarity of 81.1%, and 1.5% respectively) (Table 5), as has been reported for *L. maculans* [54].

**Table 5:** Location of gene models of *C. fulvum* in regions syntenic or non-syntenic with the *D. septosporum* genome.

	Number of gene models^a^			Percentage of gene models^b^	
	Total	Syntenic	Non-syntenic	Syntenic	Non-syntenic
All proteins^c^	14,127	9,890	4,237	70.0	30.0
BDBH^d^	11,092	8,900	2,192	89.9^g^	51.7^g^
Secreted proteins^e^	1,195	754	441	7.6^h^	10.4^h^
Secreted Cys-rich proteins^f^	271	151	120	1.5	2.8

a Values are numbers of gene models located in regions of the *C. fulvum* genome syntenic or non-syntenic with the *D. septosporum* genome as described in Materials and Methods.

b For all proteins the percentage of gene models represents the fraction of all gene models present in the *C. fulvum* genome; for other categories (BDBH, secreted proteins, secreted Cys-rich proteins) the percentage of gene models represents the fraction of gene models present in syntenic and non-syntenic regions.

c All proteins encoded by predicted gene models in the *C. fulvum* genome.

d Bi-directional best BLAST hit between *C. fulvum* and *D. septosporum* proteins with at least 50% (global) pairwise amino acid similarity and at least 60% coverage by overlap-corrected blastp HSPs.

e Gene models predicted to encode secreted proteins.

f Secreted small cysteine-rich proteins contain less than 300 amino acids of which at least four are cysteines.

g The mean amino acid similarities of all protein gene models in syntenic regions and non-syntenic regions are 85.2% and 65.1%, respectively.

h The mean amino acid similarities of secreted protein gene models in syntenic regions and non-syntenic regions are 81.1% and 60.7%, respectively.

### *C. fulvum* and *D. septosporum* share functional effectors

**Figure 4:**
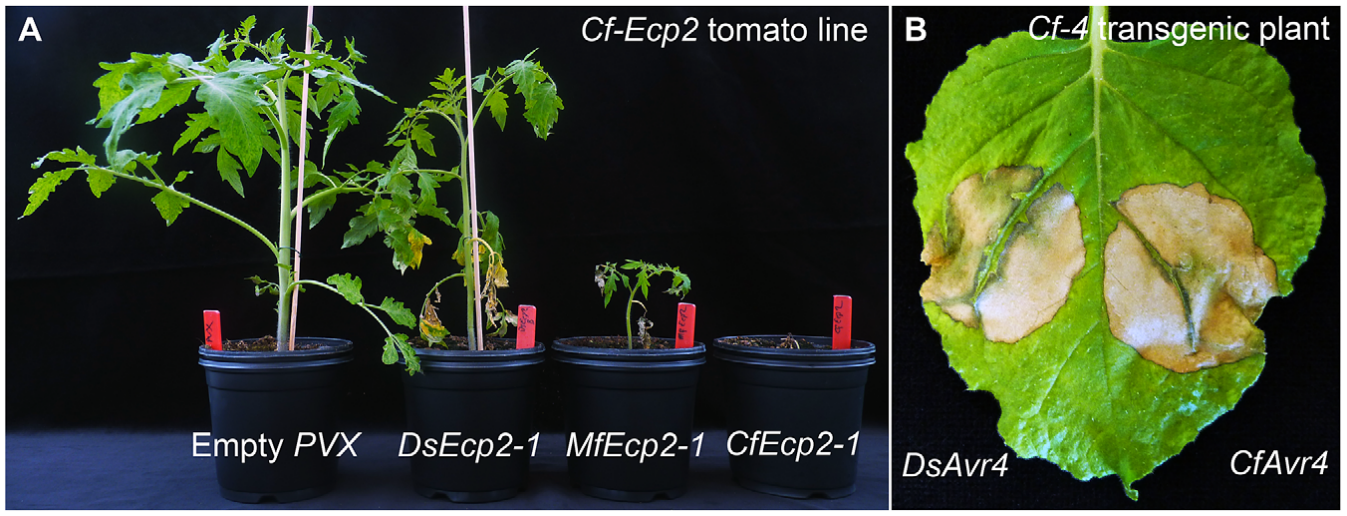
Recognition of *Dothistroma septosporum* effectors by tomato Cf receptors. A) *DsEcp2-1*, the *D. septosporum* ortholog of *CfEcp2-1*, was cloned into *pSfinx*. Tomato plants were inoculated with *Agrobacterium tumefaciens* transformants expressing *pSfinx::DsEcp2-1*. A hypersensitive response (HR) was induced in the tomato line carrying the *Cf-Ecp2* resistance gene (MM-Cf-Ecp2). Empty vector was used as a negative control and caused only mosaic symptoms. Pictures were taken at four weeks post inoculation. B) The *C. fulvum* avirulence gene *Avr4* (*CfAvr4*) and its ortholog in *D. septosporum (DsAvr4)* were heterologously expressed in *Cf-4* transgenic *Nicotiana benthamiana* using the *A. tumefaciens* transient transformation assay (ATTA). Expression of *CfAvr4* and *DsAvr4* results in an HR demonstrating that the tomato Cf-4 receptor recognizes DsAvr4. Picture was taken at six days post inoculation.

Some *C. fulvum* effector homologs have previously been reported to occur in other Dothideomycete species including *M. fijiensis, M. graminicola*, and several *Cercospora* species [33], but in the *D. septosporum* genome we found the highest number of *C. fulvum* effector homologs discovered to date, including *Avr4, Ecp2-1, Ecp2-2, Ecp2-3, Ecp4, Ecp5* and *Ecp6*. Of those, Avr4, Ecp2-1 and Ecp6 are core effectors [33] and show the highest identity (51.7%, 59.8% and 68.6% amino acid identity, respectively) with those present in *C. fulvum*, whilst *Ecp4* and *Ecp5* are pseudogenized. We were interested to know whether the *D. septosporum* effectors would be functional in triggering a Cf-mediated hypersensitive response (HR). Therefore, we inoculated plants of tomato cultivar Moneymaker (MM) carrying the Cf-Ecp2 resistance trait with *Agrobacterium tumefaciens* expressing potato virus X (PVX) containing *D. septosporum Ecp2-1* and used PVX-containing *C. fulvum Ecp2-1* as a positive control. *D. septosporum Ecp2-1* triggered a Cf-Ecp2-1-mediated HR (Figure 4A), whilst MM tomato plants lacking Cf-Ecp2 did not show any HR when inoculated with PVX containing *Ds-Ecp2-1* (results not shown). We also showed that the *D. septosporum* homolog of *C. fulvum Avr4* is functional in triggering a Cf-4-mediated HR in *Nicotiana benthamiana*, as determined with an *Agrobacterium* transient transformation assay (Figure 4B). This is remarkable because *D. septosporum* infects a gymnosperm which is only distantly related to tomato, but apparently produces effectors that can be recognized by tomato Cf resistance proteins. It would be interesting to examine whether gymnosperms carry functional homologs of the well-studied *Cf* tomato resistance gene homologs [33], [60] or other major *R* genes that could confer resistance to *D. septosporum*. Major *R* genes have been shown to be involved in resistance of some pine species to *Cronartium* spp. rust pathogens [61], [62] and are thought to function in a gene-for-gene manner [63].

**Figure 5:**
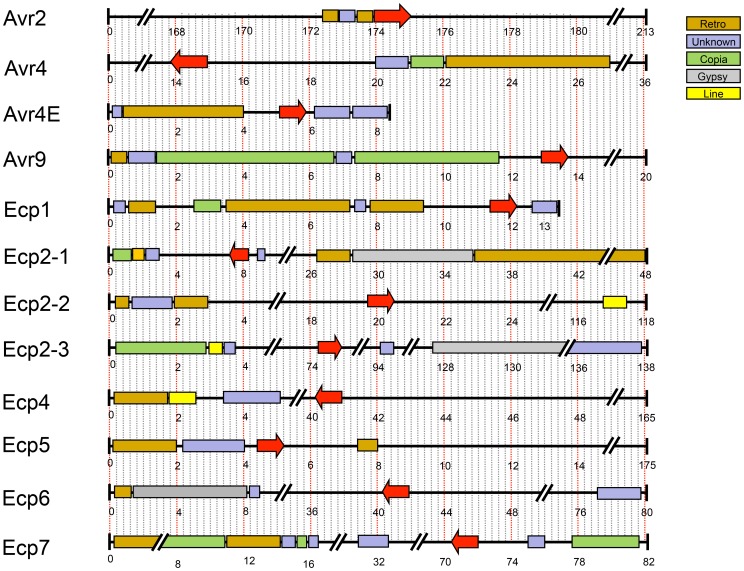
Repetitive regions flanking known effectors of *Cladosporium fulvum*. Scaffolds harboring sequenced *C. fulvum* effector genes with flanking repeats. Repeat regions longer than 200-bp are shown, with different types indicated in different color code. Red arrows depict the effector genes and sizes are in kb. The sizes of scaffolds range from 8 to 213-kb but some are shortened to fit the figure due to differences in size.

In *C. fulvum*, adaptation to resistant tomato cultivars is sometimes associated with deletion of effector genes [64]. Presence of repeats or location near a telomere can cause repeat-associated gene deletion [65]. We analyzed the location of all cloned *C. fulvum* effector genes in its genome. Many scaffolds containing an effector gene are very small (Figure 5), suggesting that they are surrounded by large repeats hampering assembly into larger scaffolds. The location of the *C. fulvum* effectors is shown in Figure 5 and the types of flanking repeats are detailed in Table S4. The well-characterized effector gene *Avr9* is located on a very small (20-kb) scaffold (Figure 5) and is likely flanked on both sides by repeats; on one side there are 11-kb of repeats on the scaffold and on the other side probably also repeats just outside the region shown that prevented further scaffold assembly. This suggests that the absolute correlation found between deletion of the *Avr9* gene in *C. fulvum* and overcoming Cf-9-mediated resistance [64] is most likely due to the close proximity of *Avr9* to large, unstable repeat regions. As well as causing deletions, transposons can contribute to genome plasticity by mutation due to transposition into coding sequences. During co-evolution, transposons have inserted into effector genes causing their inactivation and overcoming Cf-mediated resistance in *C. fulvum*, as has been reported for inactivation of both *Avr2* [64] and *Avr4E* [66]. The *C. fulvum* homologous effector genes present in *D. septosporum* are also often in close proximity to repeat-rich areas that may represent centromeres (Figure 3), but the biological significance of this is not yet clear.

Pseudogenization of two *D. septosporum* effector genes, *Ecp4* and *Ecp5*, homologous to those reported for *C. fulvum* [67], could point to host adaptation in the DNB fungus at the pine genus, species or cultivar level. Future population analysis of both fungal strains and host genotypes will reveal the mechanism behind this phenomenon.

### New hydrophobin genes in *C. fulvum*

Another class of well-studied *C. fulvum* small cysteine-rich secreted proteins is the hydrophobins. These amphipathic proteins are implicated in developmental processes in filamentous fungi and are localized on the outer surface of fungal cell walls [68]. They are divided into class I and class II hydrophobins based on sequence differences that also correlate with their different solubility [68].

Six hydrophobin genes (*Hcf-1 to Hcf-6*) had previously been identified from *C. fulvum* [66], [67]. We identified five additional hydrophobin genes in the *C. fulvum* genome [two class I (Cf187601 and Cf189770) and three class II (Cf197052, Cf188363 and Cf183780)] (Figure S3), which makes *C. fulvum* the Ascomycete species with the largest number of hydrophobin genes reported so far. In the *D. septosporum* genome only four hydrophobin genes were found, one of which (Ds75009) is predicted to encode a class II hydrophobin and was highly expressed both in culture and *in planta*. Based on EST data the 11 *C. fulvum* hydrophobin genes show a range of different expression patterns. Of the six class I *C. fulvum* hydrophobins, two were only expressed in culture [Cf184635 (*Hcf-2*) and Cf189850 (*Hcf-4*)], three were expressed both in culture and *in planta* [Cf189770, Cf187601 and Cf193176 (*Hcf-1*)], and one was not expressed in culture or *in planta* [Cf184193 (*Hcf-3*)]. Three of the class II *C. fulvum* hydrophobins were only expressed in culture [Cf197052, Cf188363 and Cf193013 (*Hcf-5*)], whilst Cf193331 (*Hcf-6*) and Cf183780 were expressed neither in culture nor *in planta*. None of the *C. fulvum* hydrophobin genes were expressed *in planta* only. It has been proposed that hydrophobins may act as ‘stealth’ factors, preventing the invading fungus from detection by its host plant [69] or protecting it against deleterious effects of plant chitinases and β-1,3 glucanases as reported for *C. fulvum* [70]. Early functional studies focused on the hydrophobin genes *Hcf-1* (Cf193176) and *Hcf-2* (Cf184635). Knocking down expression of *Hcf-1, Hcf-2*, or both genes by homology-dependent gene silencing did not compromise virulence [71],[72]; a similar result was reported for knock-down mutants of class I *Hcf-3* and *Hcf-4* and class II *Hcf-6* genes [73]. A phylogenetic tree (Figure S3) shows that the four class I genes (*Hcf-1* to *Hcf-4*) are paralogs, suggesting functional redundancy that might explain the lack of a phenotype; functional redundancy may also exist between different classes. It would be interesting to examine the role in virulence of the two most similar hydrophobin class I and class II genes of *C. fulvum* and *D. septosporum* (Cf 189770/Ds67650 and Cf197052/Ds75009, respectively) either by knock-out or knock-down strategies.

### Carbohydrate active enzyme gene and expression profiles reflect adaptation to different host plants

Because *C. fulvum* and *D. septosporum* have very different plant hosts and pathogenic lifestyles, we expected that their capacity to degrade carbohydrates would also differ and that this might be reflected in their gene complements and expression profiles. We compared numbers of genes predicted to encode carbohydrate-active enzymes (CAZymes) [74] in these two fungi to those in other fungi representative of different lifestyles. As seen for grouped families of CAZyme genes in Table 6 (e.g., GH family of glycoside hydrolases), both *C. fulvum* and *D. septosporum* have gene numbers in the same range as hemibiotrophic and necrotrophic fungi, and many more than the obligate biotroph *B. graminis* f. sp. *hordei*. Despite this, both *C. fulvum* and *D. septosporum* have fewer predicted cellulolytic enzyme genes (e.g., GH6, GH7) as well as fewer genes classified in carbohydrate binding module gene families (e.g., CBM1) than most of the other fungi shown except for *M. graminicola* (Table 6, Table S5). The reduced number of predicted genes for cell wall-degrading enzymes in *M. graminicola* was hypothesized to represent an adaptation to avoid host defenses during stealth pathogenicity [59], which also may apply to *C. fulvum* and *D. septosporum*. However, it is known that even a small number of genes can enable high levels of enzymatic activity, as has been shown for the strongly cellulolytic fungus *Trichoderma reesei* [75].

**Table 6:** Comparison of selected CAZy gene families between *C. fulvum, D. septosporum*, and five other Ascomycetes.

CAZy families^a^	Predicted function^b^	*Cf* c	*Ds*	*Mg*	*Sn*	*Mo*	*Ss*	*Bg*
		Biotroph	Hemibiotroph	Hemibiotroph	Necrotroph	Hemibiotroph	Necrotroph	Biotroph
**GH family**	**FCW/energy/PCW-C/H/HP/pectin**	**274**	**201**	**191**	**289**	**268**	**223**	**63**
GH3	PCW/FCW	19 (15, 8)	12 (12, 12)	16	16	18	13	1
GH5	PCW/FCW	16 (12, 2)	12 (11, 12)	9	18	13	14	3
GH6	PCW-C	0	0	0	4	3	1	0
GH7	PCW-C	2 (0, 0)	1 (1, 1)	1	5	5	3	2
GH10	PCW-H	2 (2, 1)	1 (1, 1)	2	7	7	2	0
GH28	PCW-pectin	15 (8, 0)	4 (4, 4)	2	4	3	17	0
GH31	PCW-H	15 (12, 0)	10 (9, 8)	7	11	6	6	1
GH32	Energy	4 (2, 1)	2 (2, 2)	4	4	4	1	0
GH35	PCW-H	6 (3, 0)	3 (3, 1)	2	4	0	4	0
GH39	PCW-H	2 (1, 0)	0	1	1	1	0	0
GH43	PCW-HP	22 (14, 3)	11 (9, 9)	10	15	20	4	0
GH78	PCW-pectin	6 (2, 2)	1 (1, 1)	2	4	3	4	1
GH88	PCW-pectin	2 (1, 0)	0	0	1	1	0	0
GH95	PCW-pectin	2 (2, 0)	0	0	2	1	1	0
**PL family**	**PCW-pectin**	**9**	**4**	**3**	**10**	**5**	**5**	**0**
PL1	PCW-pectin	3 (2, 0)	1 (1, 1)	2	4	2	4	0
PL3	PCW-pectin	3 (1, 0)	0	1	2	1	0	0
**CE family**	**FCW/PCW-H/HP/pectin**	**35**	**23**	**18**	**53**	**54**	**32**	**10**
CE5	PCW-H	11 (7, 1)	4 (3, 4)	6	11	18	8	2
**CBM family**	**FCW/energy/PCW**	**28**	**24**	**21**	**77**	**113**	**65**	**14**
CBM1	PCW	0	1 (1, 1)	0	13	22	19	0

a Predicted CAZymes were identified using the carbohydrate-active enzymes database tools (www.cazy.org). GH, glycoside hydrolases; PL, polysaccharide lyases; CE, carbohydrate esterases; CBM, carbohydrate-binding modules. Total gene numbers in these families are shown in bold. Families that are discussed in the text and differ greatly in copy number between *Cf* and *Ds* are also shown. Additional data are in Supporting Table S5.

b Where known, CAZy functions are shown as plant cell wall (PCW) or fungal cell wall (FCW) degrading and modifying enzymes, or energy-related, with substrate preferences for PCWs of cellulose (C), hemicellulose (H), hemicellulose or pectin side-chains (HP) or pectin, using classifications as in [43].

c Fungal species and their pathogenic lifestyles are shown: *Cf, C. fulvum; Ds, D. septosporum; Mg, Mycosphaerella graminicola; Sn, Stagonospora nodorum; Mo, Magnaporthe oryzae; Ss, Sclerotinia sclerotiorum; Bg, Blumeria graminis*. Numbers in parenthesis for *Cf* and *Ds* are number of genes expressed under two conditions (in culture, *in planta*) as described in Tables S12 and S13.

Next we focused on CAZyme gene families that appear to differ in gene number between *C. fulvum* and *D. septosporum*. Because small differences in gene number could be due to mis-annotation, only families that differed by two or more genes were considered and examples of these are shown in Table 6 (full data in Table S5). Potentially interesting is the expansion of genes associated with pectin degradation in *C. fulvum*. For example, in the GH28 family that includes many pectinolytic enzymes, *C. fulvum* has 15 genes whilst *D. septosporum* has only four. A higher pectinolytic activity in *C. fulvum* is concordant with the higher pectin content of its host, tomato, compared to the pine host of *D. septosporum* [76], [77], but larger numbers of genes encoding pectin-degrading enzymes have generally been associated with a necrotrophic rather than a biotrophic lifestyle in fungi [78]. High pectinolytic activity is observed in fungi such as *Botrytis cinerea* [79], [80] that invades soft, pectin-rich plant tissues causing a water-soaked appearance of the infected tissues [43]. However, during colonization of tomato leaves by *C. fulvum* this type of symptom is never observed [79], [80]. Instead of contributing to the destruction of host cell walls, the *C. fulvum* pectinolytic enzymes may facilitate local modification of primary cell walls of mesophyll cells allowing the fungus to thrive in the apoplast of tomato leaves, as suggested for the ectomycorrhizal fungus *Laccaria bicolor* that thrives on plant roots [81].

Although *C. fulvum* has a large arsenal of pectinolytic genes compared to *D. septosporum*, not all of them appear to be functional. For example, two of the six GH78 and one of the two GH88 pectinolytic genes are pseudogenized in *C. fulvum*, whilst the corresponding *D. septosporum* families do not contain pseudogenes. Another constraint to function is that gene expression appears to be tightly regulated. As shown in Table 6, none of the 15 *C. fulvum* GH28 genes appear to be expressed *in planta*, whilst all four *D. septosporum* GH28 genes are expressed. Indeed in all gene families with predicted pectinolytic function shown in Table 6 (GH28, GH78, GH88, GH95, PL1, PL3), expression *in planta* was only detected for 2 of the 31 *C. fulvum* genes, whilst all 6 genes in these pectinolytic gene families were expressed in *D. septosporum*. It is possible that *C. fulvum* pectinases are only expressed very locally to modulate complex primary cell wall structures. The location and accessibility of pectin structures embedded in the cell wall is an important consideration for its enzymatic degradation. For instance, the Basidiomycete *Schizophyllum commune* grows predominantly on beech and birch wood which is poor in pectin [82]. However, the pectin in these cell walls is concentrated around the bored pits that are used by *S. commune* to enter the wood, explaining why this fungus contains a higher number of pectinase genes than would be expected based on the overall host pectin content. Differences in pectinolytic gene content and expression between *C. fulvum* and *D. septosporum* may therefore be related to their different strategies of host invasion and subsequent colonization.

In addition to increased numbers of pectinolytic genes compared to *D. septosporum*, *C. fulvum* has more genes for enzymes that degrade hemicelluloses (e.g., families GH31, GH35 and GH39) [83] and hemicellulose-pectin complexes (GH43) (Table 6). It also contains 11 genes (compared to 4 in *D. septosporum*) encoding CE5 enzymes; these include cutinases that are required for early recognition and colonization of the host by fungal pathogens [84], [85]. The presence of so many genes encoding enzymes for plant cell wall and cuticle degradation in a biotrophic fungus like *C. fulvum* that enters its host *via* stomata is unexpected. However, the number of cutinase genes, and other secreted lipase genes is particularly low in the *D. septosporum* genome compared to other Dothideomycetes, a feature shared with the other tree pathogens *Mycopshaerella populorum* and *M. populicola* [42].

Overall our comparison shows a similar complement of CAZy genes between *C. fulvum* and *D. septosporum*, but an increased number of particular CAZyme families in *C. fulvum* including genes encoding pectin- and hemicellulose-degrading enzymes. However, a large proportion of genes in the *C. fulvum* CAZyme families lack expression *in planta* and some genes are pseudogenized.

### *C. fulvum* and *D. septosporum* share a broad range of carbohydrate substrates

**Figure 6:**
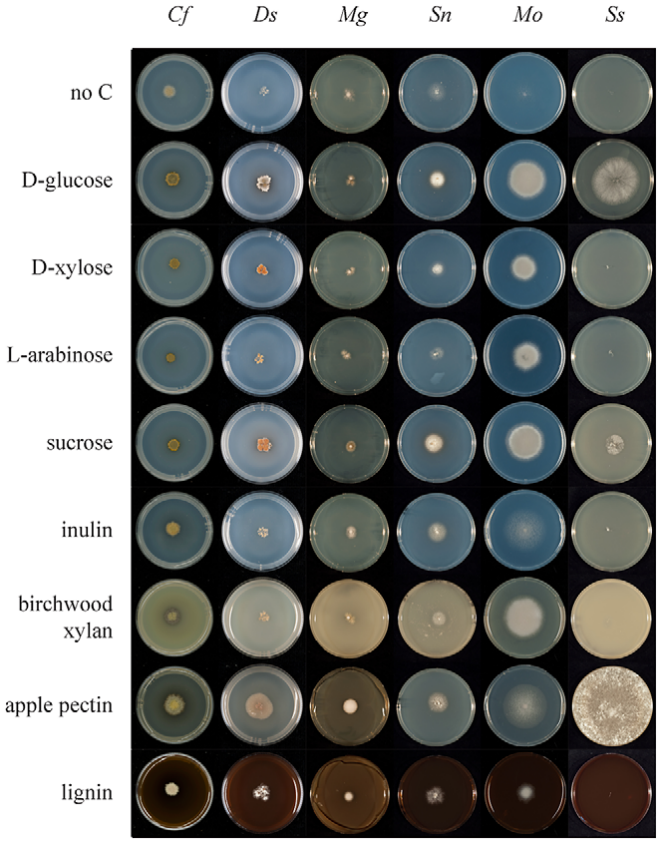
Comparative growth profiling of fungi on various carbohydrate substrates. Growth on different substrates was compared between six fungi on nine media to highlight differences. *Cf*, *C. fulvum*; *Ds*, *D. septosporum*; *Mg*, *M. graminicola*; *Sn*, *S. nodorum*; *Mo*, *Magnaporthe oryzae*; *Ss*, *Sclerotinia sclerotiorum*. D-Glucose, D-xylose, L-arabinose and sucrose were added at a final concentration of 25 mM. Birchwood xylan, apple pectin and lignin were added at a final concentration of 1% (w/v).

A second aspect of carbohydrate metabolism that we considered was a comparison of growth on defined and complex carbon substrates (Figure 6 and Figure S4; www.fung-growth.org). It was anticipated that growth profiles could illuminate differences between pathogens with dicot and gymnosperm hosts and show correlations with their respective gene complements. In a study of polysaccharide hydrolysis activities of many fungal pathogens, King et al. [86] showed preferential substrate utilization based on host specificity (dicot or monocot). In general *D. septosporum* grows more slowly on minimal control medium [87] than *C. fulvum*, but surprisingly overall the growth profiles of the two fungi are similar on most substrates (Figure 6 and Figure S4; Table S6) and both appear to utilize a broader range of substrates than *M. graminicola* (Figure 6). This is not only the case for the oligomeric and polymeric carbon substrates, requiring CAZymes for degradation, but also for monomeric carbon substrates, suggesting a diverse and efficient carbon catabolism in *C. fulvum* and *D. septosporum*. The good growth of *D. septosporum* on sucrose is particularly striking, suggesting that it can utilize sucrose available in apoplastic fluid during its early biotrophic colonization phase.

In terms of complex carbon sources, *D. septosporum* shows a slightly better capacity than *C. fulvum* to utlise apple and citrus pectin (Figure 6 and Figure S4). This seems to contradict the higher pectinolytic gene numbers in *C. fulvum* compared to *D. septosporum*, but is supported by the expression of fewer *C. fulvum* pectinolytic genes during infection of tomato when compared to the *D. septosporum* pectinolytic genes during infection of pine needle (Table 6). Interestingly, good growth on pectin is also observed for *M. graminicola*, despite an even lower number of putative pectinases than *D. septosporum*. This suggests that regulation of expression is a more dominant factor in pectin degradation by these plant pathogens than the number of pectinase-encoding genes in their genomes. In contrast, pectinase gene numbers correlate well with growth profiles of *Aspergillus nidulans*, *A. oryzae* and *A. niger* [88]. Compared to growth on controls lacking a carbon source, *D. septosporum* also showed slightly better growth than *C. fulvum* on lignin. This would be consistent with the higher proportion of lignin in pine needles, estimated to be 25–30% of dry weight [89], compared to less than 10% in dicots [90]. However, due to the very slow growth of both fungi and the non-uniform growth habit of *D. septosporum* on these media, firm conclusions about their abilities to utilize lignin cannot be made.

### Adaptations for coping with chemical and structural defences

Tomato plants produce the antimicrobial saponin, tomatine. The tomato pathogen *Fusarium oxysporum* produces α-tomatinase, which functions as a virulence factor as it degrades tomatine into the non-toxic compounds tomatidine and lycotetraose [91]. A gene predicted to encode α-tomatinase, classified as a GH10 enzyme, was found in the *C. fulvum* genome (JGI ID 188986) but is absent from the *D. septosporum* genome. Another gene found only in *C. fulvum* shows predicted similarity to the GH5 family enzyme hesperidin 6-O-α-L-rhamnosyl-β-glucosidase that can degrade hesperidin [77]. Hesperidin occurs most abundantly in citrus fruits [92] and is a member of the flavonoid group of compounds that is well known for its antimicrobial activity. Flavonoid-degrading enzymes such as hesperidin 6-O-α-L-rhamnosyl-β-glucosidase might enable *C. fulvum* to detoxify hesperidin or related compounds present in tomato.

Chemical defence molecules in pine needles include antimicrobial monoterpenes. Thus it is expected that *D. septosporum* is adapted to tolerate or degrade these compounds whilst *C. fulvum* is not. Recent work on the pine pathogen *Grosmannia clavigera* revealed several classes of genes that are upregulated in response to terpene treatment [93]. After 36 h, major classes of upregulated genes included those involved in β-oxidation as well as mono-oxygenases and alcohol/aldehyde dehydrogenases that may be involved in activating terpenes for β-oxidation. A drug transporter, GLEAN_8030, was functionally analyzed and found to be required for tolerance of the fungus against terpenes, enabling *G. clavigera* to grow on media containing these compounds. A search for three of these genes, including GLEAN_8030, showed that both *C. fulvum* and *D. septosporum* genomes contain putative homologs and share a similar gene complement to each other (Table S7). However, since these genes have not all been functionally characterized in *G. clavigera* and all are predicted to encode proteins involved in general metabolic processes, further work is required to determine the roles of the homologs found in both *C. fulvum* and *D. septosporum*.

As well as chemical mechanisms, plants employ basal structural defence mechanims including lignification of cell walls [94], [95]. Due to the abundance of lignin in pine needles that block access to usable cellulose, fungal pathogens and saprophytes living on pines have a particularly challenging environment [96]. For *D. septosporum* to complete its lifecycle, degradation of pine needle tissue must occur so that conidiophores bearing conidia can erupt through the epidermis (Figure 1H), which contains lignin [97]. This is in contrast to *C. fulvum* whose conidiophores emerge from tomato leaves through stomatal pores (Figure 1D). Thus, we investigated genes that may be involved in lignin degradation.

Some saprophytic fungi utilize oxidoreductases, particularly class-II peroxidases such as lignin peroxidases, manganese peroxidases and laccases, and a number of H_2_O_2_-producing enzymes, to achieve lignin breakdown [98], [99]. However, the number of genes encoding oxidoreductases in *D. septosporum* is no higher than those of other Dothideomycetes (*C. fulvum, M. graminicola* and *S. nodorum*) that infect plants with lower levels of lignin (Table S8). *D. septosporum* appears to have a similar complement of laccase genes as *C. fulvum* and only one distant relative of a class-II peroxidase, missing in *C. fulvum*, but also present in *M. graminicola* and *S. nodorum*. Interestingly, the classical Ascomycete laccases found in *C. fulvum, D. septosporum* and *M. graminicola* bear a carbohydrate-binding domain (CBM20, putative starch binding domain). This type of laccase is only found in Dothideomycetes but the significance of this novel modular structure is unclear. Brown-rot saprophytes such as *Serpula lacrymans* have a reduced complement of ligninolytic genes compared to lignin-degrading white-rot fungi and are proposed to initially weaken lignocellulose complexes by non-enzymatic use of hydroxyl radicals prior to enzymatic assimilation of accessible carbohydrates [81]. It is likely that *D. septosporum* uses a similar strategy to breach the lignin-rich components of pine needles, as complete degradation of this polymer is not required to complete its life cycle.

### The secondary metabolite gene complement of *C. fulvum* and *D. septosporum*

Secondary metabolites (SMs) are important compounds for the colonization of specific ecological niches by fungi. In particular, plant-pathogenic fungi can produce non-specific and host-specific toxic SMs [100]. SMs also include mycotoxins that contaminate food and feed and are harmful to mammals [100]. The only currently known SMs produced by *C. fulvum* and *D. septosporum* are cladofulvin and dothistromin, respectively [101], [102]; both compounds are anthraquinone pigments. In fungi, SM biosynthetic pathways often involve enzymes encoded in gene clusters [103] and always require the activity of at least one of four key enzymes: polyketide synthase (PKS), non-ribosomal peptide synthetase (NRPS), terpene cyclase (TC) or dimethylallyl tryptophan synthase (DMATS) [104]. It has been suggested that loss of SM biosynthetic pathways is associated with biotrophy [44], thus we searched for SM gene pathways in both genomes. Surprisingly, the biotroph *C. fulvum* has twice the number of key SM genes (23 in total) compared to the hemibiotroph *D. septosporum* (11 in total) (Table 7), of which 14 and 9, respectively, are organized into gene clusters along with other SM-related genes. The numbers of key SM enzyme-encoding genes are comparable to those of *M. graminicola*, but are lower than those in most other sequenced Dothideomycetes [42]. Like all Ascomycetes [105], the majority of key SM enzymes in *C. fulvum* and *D. septosporum* are PKSs, NRPSs and hybrid PKS-NRPSs. Annotation of all key SM genes was manually checked and two truncated (*Pks4* and *Nps1*) and five pseudogenized (*Pks9*, *Hps2*, *Nps5*, *Nps7* and *Nps10*) genes were found in the *C. fulvum* genome, while all *D. septosporum* genes except *Pks4* (truncated) are predicted to encode functional enzymes. Overall, the number of predicted functional pathways suggests that *C. fulvum* and *D. septosporum* can produce at least 14 and 10 different SMs, respectively.

**Table 7:** Key secondary metabolism genes in Ascomycete genomes.

Fungal species	Lifestyle	PKS	NRPS	Hybrid	TC	DMATS	Total
*Cladosporium fulvum*	Biotroph	10	10	2	0	1	23
*Dothistroma septosporum*	Hemibiotroph	5	3	2	0	1	11
*Mycosphaerella graminicola*	Hemibiotroph	11	6	2	1	0	20
*Stagonospora nodorum*	Necrotroph	22	10	2	2	2	38
*Magnaporthe oryzae*	Hemibiotroph	22	8	10	3	3	46
*Fusarium graminearum*	Necrotroph	13	12	2	3	0	30
*Aspergillus nidulans*	Saprophyte	26	10	2	5	2	45
*Neurospora crassa*	Saprophyte	6	3	1	1	1	12

Numbers of predicted polyketide synthase (PKS), non-ribosomal peptide synthetase (NRPS), hybrid PKS-NRPS (Hybrid), terpene cyclase (TC) and dimethylallyl tryptophan synthase (DMATS) genes are shown. Data are from this study and from Collemare et al. 2008 [105].

Surprisingly, only three of the key SM genes are predicted to belong to biosynthetic pathways shared between the two species (Table S9) suggesting a diverse SM repertoire. This is much lower than expected given the overall level of similarity in gene content between the two genomes, and suggests that this SM repertoire is under strong selection. The three common genes are predicted to be involved in production of a pigment related to melanin (*Pks1*), a siderophore (*Nps2*) and dothistromin (*PksA*) based on similarities to other characterized genes. In *C. fulvum*, the three other functional non-reducing PKS enzymes are candidates for production of cladofulvin.

The genomic locations of the 11 biosynthetic SM genes in *D. septosporum* do not show any enrichment at sub-telomeric positions, as reported for *Aspergillus* spp. and *Fusarium graminearum* [106], [107], or near putative centromeres (Figure 3). However, 8 out of the 11 genes are located on chromosomes smaller than 2-Mb (chromosomes 8 to 12; Figure 3). The genomic regions immediately surrounding all 11 *D. septosporum* SM genes are conserved in the *C. fulvum* genome, although 8 of them lack the key SM gene itself and, sometimes, putative accessory genes also (Figure S5). Reciprocally, only 9 *C. fulvum* SM genomic regions out of 23 are conserved in *D. septosporum* with 6 of these lacking the key SM gene, suggesting either gain or loss of SM genes has occurred. For two of the regions where flanking genes are conserved but SM gene(s) are missing in *C. fulvum* (regions corresponding to those surrounding *Pks3* and *Nps3* in *D. septosporum*), the presence of repeats suggests that SM gene loss may have occurred in *C. fulvum* (Figure S5). The *C. fulvum*-specific SM loci *Pks5*, *Pks6*, *Nps5*/*Dma1* and *Nps9* include many transposable elements and genes that have similarity to genes scattered in the *D. septosporum* genome, often on the same chromosome, leading to the hypothesis that these SM loci were assembled by gene relocation as recently proposed for the fumonisin gene cluster in *F. verticillioides* [108].

### Dothistromin toxin genes are present in both genomes

**Figure 7:**
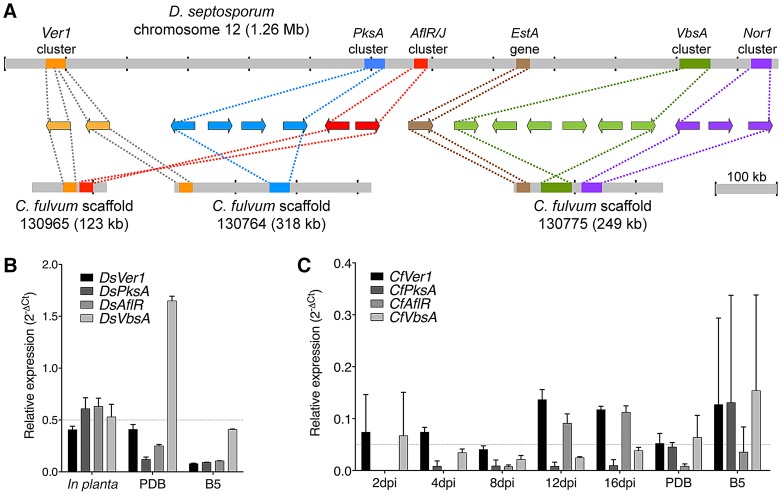
Arrangement of predicted dothistromin genes in *Dothistroma septosporum* and *Cladosporium fulvum*. A) Predicted dothistromin genes within the labeled clusters (left to right) are: *Ver1, DotC* (*Ver1* cluster); *PksA, CypX, AvfA, MoxY* (*PksA* cluster); *AflR, AflJ* (*AflR/J* cluster); *OrdB*, *AvnA, HexB, HexA, HypC, VbsA* (*VbsA* cluster); *Nor1, AdhA, VerB* (*Nor1* cluster). Positions of mini-clusters are approximate and they are not drawn to scale. Dothistromin genes within the published *D. septosporum PksA* and *VbsA* clusters [36], [38] and the newly discovered *AflR/J* and *Nor-1* clusters are found in the same order and orientation in *C. fulvum*. B) Expression of dothistromin biosynthetic genes (*Ver1, PksA, VbsA*) and regulatory gene (*AflR*) was determined in *D. septosporum* by quantitative PCR. Mean expression and standard deviations are shown for at least 3 biological replicates relative to β-tubulin expression. In *D. septosporum* all genes but *DsVbsA* are expressed more highly *in planta* (late-stage sporulating lesions from a forest sample) than in culture (PDB or B5 media) as highlighted by the dashed-grey line. C) Expression of *C. fulvum* genes is shown as for (B), revealing that expression is not higher during tomato infection than in culture (dashed-grey line). Note the different scales for expression, which reveal a much lower level of transcription both *in planta* and in PDB medium compared to *D. septosporum*.

Analysis of the *D. septosporum* 1.3-Mb chromosome 12 revealed that the three previously identified mini-clusters of dothistromin genes [38] are widely dispersed, confirming fragmentation of this gene cluster (Figure 3 and Figure 7). Candidates for additional dothistromin genes, previously predicted based on aflatoxin pathway genes [39], are also present. Although three of these genes (*OrdB, AvnA, HexB*) are located in the published *VbsA* mini-cluster, the others are dispersed over different regions of chromosome 12 as shown in Figure 7. The end of the *Nor1* gene cluster (*Nor1, AdhA, VerB*) is less than 10-kb from one predicted telomere, whilst *Ver1* (previously called *dotA* [35]) is only 81-kb from the other telomere. As expected, a gene similar to the aflatoxin *AflR* regulatory gene is present and, like in aflatoxin-producing species of *Aspergillus*, is divergently transcribed with an adjacent *AflJ* regulatory gene candidate. Functional analysis of these genes is in progress.

Although *C. fulvum* is not known to produce dothistromin, the complete set of predicted dothistromin genes is present in its genome, encoding proteins with amino acid identities ranging from 49% (AflJ) to 98% (Ver1) when compared with those of *D. septosporum* (Table S10). The arrangement of predicted dothistromin genes in *C. fulvum* reveals a high level of synteny with some rearrangements. With the exception of the *Ver1* gene cluster, the mini-clusters contain the same genes in the same orientations in the two species (Figure 7A). The three mini-clusters on *C. fulvum* scaffold 130775 are much closer together than in *D. septosporum*, but are still separated from each other by considerable distances (approximately 24-kb between *Est1* and the *VbsA* gene cluster, and 40-kb between the *VbsA* and *Nor1* gene clusters). A comparison of the relative locations of the mini-clusters in the two species suggests inversions (*AflR/J* and *VbsA* gene clusters) as well as rearrangements over relatively small (*VbsA-Nor1*) and large (*Ver1-AflR/J*) distances. This is consistent with the overall pattern of intrachromosomal rearrangements observed between these two genomes.

Given the presence of the dothistromin biosynthetic pathway genes, we tested whether dothistromin is produced by *C. fulvum*. However, no dothistromin was detected by HPLC analysis of extracts from *C. fulvum* PDB cultures, which is a condition favorable to dothistromin production by *D. septosporum*. Despite the lack of dothistromin production under these conditions, a strong evolutionary constraint on dothistromin biosynthetic genes was seen by analyzing the ratio of non-synonymous to synonymous mutations (Ka/Ks) between *C. fulvum* and *D. septosporum*. The low Ka/Ks ratios seen for dothistromin genes (range 0.018–0.169) are indicative of purifying selection [109] and did not differ from the distribution observed for four housekeeping genes (*Tub1, Eif3b, Pap1, Rps9*; range 0.003–0.073) (P = 0.561). Evidence for purifying selection was also shown for aflatoxin pathway genes in *Aspergillus flavus* and *A. nomius* [110]. On the basis of this we propose that *C. fulvum* might produce dothistromin, or a metabolite related to dothistromin, under certain environmental conditions when it is required.

### Regulation of secondary metabolite biosynthetic pathways suggests lifestyle adaptation at the transcriptome level

Many fungal SM biosynthetic pathways are cryptic, meaning that they are not expressed in wild-type strains under laboratory conditions. However, manipulation of genetic regulatory pathways or environmental conditions has shown that some of these cryptic pathways are functional [111], [112].

As seen for other gene families such as CAZyme genes, *C. fulvum* appears to be more economical in its expression of SM genes than *D. septosporum*, particularly *in planta*. In *C. fulvum*, EST support was obtained from *in vitro* conditions for all key SM genes except *Hps2*, *Nps7* and *Nps10*, which are pseudogenized. The two truncated genes (*Pks4* and *Nps1*) and the pseudogenized *Nps5* genes also have EST support but the resulting proteins are unlikely to be functional. However, no evidence for *in planta* expression could be obtained for any of the *C. fulvum* key SM genes from this EST library. In contrast, all *D. septosporum* key SM genes have EST support from both *in vitro* and *in planta* libraries, with the unique *DsPKS2* being one of the most highly expressed genes during pine needle infection.

Differences in dothistromin pathway regulation were confirmed by quantitative PCR. In *D. septosporum*, *Ver1*, *PksA*, *AflR* and *VbsA* show higher expression during pine infection than in controlled culture conditions used to induce dothistromin production (Figure 7B). In contrast, the same genes show a low expression level in *C. fulvum* during infection and *in vitro* (Figure 7C). Because no dothistromin could be detected *in vitro*, this low expression likely represents background transcription with no biological relevance. Such an expression pattern is significantly different from the upregulation of *Avr4* and *Avr9* genes during tomato infection (Figure S6).

SM production is associated with development in fungi and involves common regulators [113]. We searched the genomes of *C. fulvum* and *D. septosporum* for conserved regulators of development and SM production and, based on predicted protein sequences, found clear homologs for most of these genes in both fungi (Table S11). The two species appear to lack a PpoB oxygenase, but *PpoA* and *PpoC* are sufficient to produce all *psi* factors (oxylipins) identified in *Aspergillus* species [114]. In addition, *C. fulvum* lacks clear homologs of the G-protein regulators FlbA and RgsA, while possible homologs are found in *D. septosporum*. In *Aspergillus* species both proteins are negative regulators of G-protein signaling pathways. Neither *C. fulvum* nor *D. septosporum* have a homolog of *BrlA*, an essential regulator of conidiation in *Aspergillus* species [115], suggesting that they use another regulator for this role. Future studies analyzing expression of the SM genes, and the roles of regulatory genes, will help to determine fundamental differences in how *C. fulvum* and *D. septosporum* differentially regulate their SM gene expression.

### Conclusion

We embarked upon a comparative genomics analysis of *C. fulvum* and *D. septosporum* to test for differences that might explain their host specificity and lifestyles. The comparison revealed surprising similarities, such as the presence of dothistromin toxin genes in *C. fulvum* and functional *Avr4* and *Ecp2* effector genes in *D. septosporum*. However, the genome sizes of the two fungi are remarkably different, mainly due to a vast expansion of transposable elements in *C. fulvum*, and show several key differences in gene content. Adaptation of *C. fulvum* to its host plant tomato is exemplified by the specific presence of a gene encoding α-tomatinase, likely involved in degradation of tomatine. In contrast, the dothistromin gene cluster is present in both fungi, but while it is strongly expressed in *D. septosporum* at late stages of pine needle infection, it is lowly or not expressed in *C. fulvum* during infection of tomato leaves. Both fungi contain additional key SM genes, but the majority of these are not in common, contrasting with the high degree of homology between the two genomes. We suggest that this lack of conservation of key SM genes in the *C. fulvum* and *D. septosporum* genomes is a consequence of different evolutionary pressures that result from their different lifestyles, either as a pathogen inside their host or possibly as a saprophyte outside their host.

Another key difference between the two fungi during pathogenesis concerns their differential gene regulation. Gene expression in *C. fulvum* is strictly regulated *in planta*, with many SM, hydrophobin and CAZy genes not expressed, while expression in *D. septosporum* is more constitutive. This differential regulation of expression may be crucial in determining differentiation between these fungi despite very similar gene profiles. Furthermore, this expression pattern is consistent with a biotrophic lifestyle without gene loss. Finally we suggest that the higher repeat content of the *C. fulvum* genome, along with evidence for gene pseudogenization (van der Burgt A et al., unpublished data) has facilitated the evolution of different lifestyles between *C. fulvum* and its sister species *D. septosporum*. Overall, our comparison of the two genomes suggests that even closely related plant pathogens could adapt to very different hosts and lifestyles by differentiating gene content and regulation, whilst retaining genetic signatures of a common ancestral way of life.

## Materials and Methods

### Fungal strains and growth conditions

The fungal strains of *C. fulvum* (race 0WU; CBS131901) and *D. septosporum* (strain NZE10; CBS128990) were isolated from tomato growing in an allotment garden in Wageningen, The Netherlands, in 1997, and from a needle from an eight-year-old *Pinus radiata* tree on the West Coast of the South Island of New Zealand in 2005, respectively. Monospore cultures, whose identities were confirmed by ribosomal ITS sequencing, were used throughout. Unless specified otherwise, cultures of these fungi were maintained on potato dextrose agar (PDA) or potato dextrose broth (PDB) media (*C. fulvum*) or Dothistroma Medium (DM; 5% w/v malt extract, 2.8% w/v nutrient agar or nutrient broth) at 22°C prior to use. Growth conditions used for generation of EST libraries (Protocol S1) are shown in Tables S12 and S13. Cultures were maintained for long-term storage in closed vials at −80°C stocks in 20% glycerol.

### Tomato infections

Conidia of *C. fulvum* were harvested from two-week-old PDA plates with distilled water. The conidial suspension was filtered through Calbiochem Miracloth (EMD Millipore Chemicals, Philadelphia, PA) and washed once with water prior to calibration to 5×10^5^ conidia/mL. Five-week-old Heinz tomato plants were sprayed on the lower side of the leaves with the conidial suspension (10 mL per plant). The plants were kept at 100% relative humidity for 48 h. The plastic-covered cages were then opened to grow the plants under regular greenhouse conditions (70% relative humidity, 23–25°C during daytime and 19–21°C at night, light/dark regime of 16/8 h, and 100 W/m^2^ supplemental light when the sunlight influx intensity was less than 150 W/m^2^). The 4^th^ composite leaves of infected tomato plants were harvested at 2, 4, 8, 12 and 16 dpi, and immediately frozen in liquid nitrogen.

### Phylogenetic comparison of fungal species

To highlight the phylogenetic relationships of *C. fulvum* and *D. septosporum* with Dothideomycetes and other fungi relevant to this study, conserved protein families were predicted by use of the MCL Markov clustering program [116] with pairwise blastp protein similarities and an inflation factor of 4. From this multi-gene family set, 51 orthologous groups of genes were identified. Predicted protein sequences were concatenated, aligned using MAFFT 6.717b [117] and a species tree calculated using RAxML 7.2.8 [118]. We also determined protein homology data based on bidirectional best hits when comparing the proteomes of eleven Dothideomycete species (*Alternaria brassicicola*, *C. fulvum*, *Cochliobolus heterostrophus*, *D. septosporum*, *Hysterium pulicare*, *Mycosphaerella fijiensis*, *Mycosphaerella graminicola*, *Pyrenophora tritici-repentis*, *Rhytidhysteron rufulum*, *Septoria musiva* and *Stagonospora nodorum*), together with four out-group species (*Aspergillus nidulans*, *Fusarium graminearum*, *Neurospora crassa* and *Magnaporthe grisea*).

### Repetitive sequences and transposable elements

Repeat sequences in both genomes were identified using RECON [119]. To group repetitive elements together into different families the default RECON output was parsed to include families with 10 or more elements. The parsed RECON repeat library was used to determine the extent of the repetitive fraction in the *D. septosporum* and *C. fulvum* genomes using RepeatMasker [120] and to annotate repetitive families and identify structural features, such as Long Terminal Repeats (LTRs) and Terminal Inverted Repeats (TIRs), using BLAST.

### Repeat-induced point mutation (RIP)

Sequences that had undergone Repeat-Induced Point mutation (RIP) were identified according to the composite RIP index (CRI) method [121]. The CRI was calculated for each 500-nt sequence window, which was shifted at each 25-nt step. Sequences were identified as having been subjected to RIP when the RIP product, RIP substrate and composite RIP indices were at least 1.2, at most 0.8 and at least 1.0 respectively. As a final constraint, a series of overlapping sequence windows had to exceed 750 nt in length and the CRI value of any of the windows peaked to 1.5 in order to be scored as a RIP'd locus.

### Syntenic and non-syntenic regions

Syntenic regions shared between *C. fulvum* and *D. septosporum* were detected *ab initio* on their repeat-masked genome sequences using promer [122], blastp and a suite of custom made python scripts. A script called blastpmer obtained all translated ORFs above a threshold nucleotide length from both query and subject genomes, performed a blastp on these ORFs, and subsequently filtered on expected value and high-scoring segment pair (HSP) length. Protein matches (using *C. fulvum* as query and *D. septosporum* as subject) were obtained with promer (–maxmatch) and blastpmer (–ORF 500 nt –HSP 250 nt –expect 1e-9). Both genomes were masked for these protein matches before being subjected to a second round of searching for weaker and shorter protein similarities, again using promer (–b 50 –c 15 –l 5 –maxmatch) and blastpmer (–ORF 300 nt –HSP 110 nt –e 1e-7). These four searches yielded 57,270, 44,865, 1,864 and 2,367 matches, respectively, many of which were redundant and overlapping. This large set was reduced to 24,480 unique matches by removing all except the best alignment for each unique genomic locus. This step removed overlapping alignments with different phases or orientations, and excluded suboptimal alignments caused by paralogs and common protein domains. The product of amino acid similarity and match length was employed as a final alignment quality score. Matches were ordered by query scaffold position and joined into linked syntenic regions according to the following criteria: (i) adjacent matches were identical on the query and subject scaffolds; (ii) matches had the same strand orientation; and (iii) maximum and average nucleotide distance between adjacent matches on the query and subject scaffolds were <10-kb and <5-kb, respectively. This step resulted in a reduction to 1,875 collinear match regions, of which 1,277 were >5-kb. For comparison of protein-coding genes in syntenic versus non-syntenic areas, gene models were classified as syntenic if they overlapped with any of the 1,875 collinear syntenic areas. Thus, subsets of 9,890 syntenic and 4,237 non-syntenic genes were inferred for *C. fulvum*.

To investigate mesosynteny on a whole-genome scale, a refined synteny dataset was created with correction for inversions and rearrangements, and removal of spurious, small alignments. Match regions were compared and merged further if (i) adjacent groups had opposite orientations; or (ii) groups with identical query and subject scaffolds were separated by at least one (group of) matches on a conflicting subject scaffold, but maximum and average nucleotide distances between match regions were at most 20-kb or on average <10-kb apart; and finally (iii) match regions <5-kb were rejected. The final refined dataset contained 1,103 syntenic regions between 5 and 226-kb (average 22,194-bp), representing 22,700 matches from the original 24,480 unique matches.

### *C. fulvum* and *D. septosporum*-specific proteins

To identify potential *C. fulvum* and *D. septosporum*-specific proteins, the total protein sets from both fungi were used in comparative blastMatrix [123] searches against sequences from the nine additional members of the Dothideomycetes listed in the phylogenetics section.

### *C. fulvum* and *D. septosporum* secretome analysis

Initially, subcellular localizations for all *C. fulvum* and *D. septosporum* proteins were predicted using WoLF PSORT (wolfpsort.org; [124]). Only proteins containing a signal peptide and a signal peptide cleavage site, but lacking transmembrane (TM) domains or proteins containing a single TM that overlaps with the secretion signal, were selected. Signal peptides and cleavage sites were predicted using SignalP version 3.0 [125], where a final D-Score cut-off of 0.5 was used to increase specificity while retaining sensitivity. Subsequently, all proteins with signal peptides (1,886 and 1,591 for *C. fulvum* and *D. septosporum*, respectively) were analyzed for the presence of TM domains using the web servers Phobius [126] and TMHMM (version 2.0; [127]). The servers identified different, partially overlapping, sets of proteins with putative TM domains. On average Phobius detected 22% more TM domain proteins than did TMHMM, and about 75% of the predictions were shared between the servers. For further analyses, all proteins with putative TM domains as predicted by either of the two servers were removed from the dataset. Then, the proteins that contain a putative mitochondrial targeting signal as predicted by TargetP version 1.1 [128] were removed. Finally, proteins containing a potential GPI-anchor signal as predicted by the PredGPI web service were discarded [129].

### Functional analysis of *D. septosporum Avr4* by *A. tumefaciens*-mediated transient gene expression in *N. benthamiana*

A *C. fulvum Avr4* (*Cf-Avr4*) gene homolog was identified in the genome of *D. septosporum* (*Ds-Avr4*) by blastp, with an E-value of 1×10^−4^. To determine Cf-4-mediated HR-inducing ability of Ds-Avr4 of *D. septosporum*, the *Agrobacterium tumefaciens*-mediated transient gene expression assay (ATTA) was performed in *N. benthamiana* as described by Van der Hoorn et al. [130]. The *Cf-Avr4* and *Ds-Avr4* genes were each fused to a *PR-1A* signal peptide sequence [131] for secretion into the apoplast. Subsequently a Gateway cloning strategy was performed to clone them into a *pK2GW7* binary expression vector [132] containing the CaMV 35S promoter. *A. tumefaciens* (strain GV3101) was finally transformed with *pK2GW7* binary vectors containing *Cf-Avr4* or *Ds-Avr4* genes by electroporation. Agroinfiltration of *Cf-4* transgenic *N. benthamiana* leaves with *Cf-Avr4-* and *Ds-Avr4*-containing *A. tumefaciens* clones was performed as described by van der Hoorn et al. [130]. Photographs were taken at six days post inoculation.

### Heterologous expression of the *D. septosporum Ecp2* (*Ds-Ecp2-1*) gene in MM-Cf-Ecp2 tomato plants

Three *D. septosporum* homologs of *C. fulvum Ecp2* genes (*Ds-Ecp2-1, Ds-ecp2-2* and *Ds-Ecp2-3*) were identified as described for *Avr4*. A binary Potato Virus X (PVX)–based vector, *pSfinx*, was used for transient expression of the *Cf-Ecp2-1* ortholog, *Ds-Ecp2-1*, in MM-Cf-Ecp2 tomato lines based on methodology described by Hammond-Kosack et al. [131]. The recombinant viruses were obtained by cloning *Ds-Ecp2-1* (an intron-less gene), encoding the mature protein, downstream of the *PR-1A* signal sequence for secretion into the apoplast and under the control of the CaMV 35S promoter. Recombinant *pSfinx::Ecp2-1*, corresponding to the *C. fulvum Ecp2* (*Cf-Ecp2-1*), and *pSfinx::Empty* viruses were as published [33]. *A. tumefaciens* (GV3101) was transformed with the *pSfinx::Ds-Ecp2-1* construct by electroporation. *A. tumefaciens* strains containing the *pSfinx* constructs for the expression of Cf-Ecp2-1 and Ds-Ecp2-1 proteins were inoculated on MM-Cf*-Ecp2* tomato lines containing the cognate *R* gene, and MM-Cf-0 tomato lines that contain no *R* genes, mediating recognition of the Ecp2-1 effector. Photographs were taken four weeks post inoculation.

### Analyses of hydrophobin-encoding genes

All six previously reported hydrophobin genes from *C. fulvum* [133] were found in the automated gene predictions performed on the genome sequence. Five of the hydrophobins (Hcf-1 to Hcf-5) are predicted to contain an interpro motif common in fungal hydrophobins (IPR001338), while Hcf-6 has an interpro motif, which is restricted to Ascomycetes only (IPR010636). To identify putative hydrophobin-encoding genes in other genomes, all secreted gene models of *C. fulvum, D. septosporum* and *M. graminicola* were computationally annotated using Interpro scan and Gene Ontology terms. Then, gene models with IPR001338 and IPR010636 Interpro scan terms were identified as putative hydrophobin candidates. Also, a HMM profile search (which was built based on the conserved cysteine motifs in class I hydrophobins) was performed to identify hydrophobins missed by standard similarity searches. In this way five additional hydrophobin genes were identified in the *C. fulvum* genome. Hydrophobin sequences were aligned with ClustalW and edited in GeneDoc software. Then a consensus phylogenetic tree of predicted hydrophobin amino acid sequences was constructed using MEGA5 software [134] performing the minimum-evolution algorithm with default parameters and 1000 bootstrap replications.

### Analyses of carbohydrate-active (CAZy) enzymes

The carbohydrate-active enzyme catalogs of *C. fulvum* and *D. septosporum* were compared with the corresponding catalogs from other Dothideomycete fungi [42]. The boundaries of the carbohydrate-active modules and associated carbohydrate-binding modules of the proteins encoded by each fungus in the comparison were determined using the BLAST and HMM-based routines of the Carbohydrate-Active-EnZymes database ([74]; www.cazy.org). For determining the growth profiles on different carbohydrate substrates Aspergillus minimal medium [87] adjusted to pH 6.0 and containing 1.5% agar (Invitrogen, 30391–049) was used. Carbon sources were added at concentrations as indicated in the text and using standard methods as described at www.fung-growth.org. Duplicate plates were inoculated with 2 µL of a suspension containing 500 conidia/µL. Cultures were grown at 22–25°C for two weeks for *C. fulvum* and four weeks for *D. septosporum*, and representative plates were photographed.

### Secondary metabolite gene analysis

Genes encoding polyketide synthases (PKSs), non-ribosomal peptide synthases (NRPSs), hybrids of PKS and NRPS, terpene cyclases (TCs) and dimethylallyl tryptophan synthases (DMATSs) were sought in the two genomes using tblastn/blastp and several Ascomycete protein sequences as queries (Ace1 for PKS and hybrids; MGG_00022.7 protein for NRPS; tri5, cps/ks, all TCs from *B. cinerea* for TCs; Dma1 from *Claviceps purpurea* for DMATSs). For each tblastn/blastp hit, search for conserved domains (CDS at NCBI, InterproScan) and blastp analysis at NCBI and InterproScan confirmed the functional annotation. The locus of each key gene was analyzed for genes that could potentially be involved in a biosynthetic pathway. Functional annotation of downstream and upstream genes was confirmed using blastp at NCBI. In addition, homologs to genes that were shown to be involved in the regulation of fungal development and secondary metabolism were sought using tblastn/blastp with the sequences of the characterized proteins as queries.

Ka/Ks calculations were carried out to estimate evolutionary constraints on putative dothistromin genes (*PksA, VbsA, Ver1, HexA, AvfA, CypA* and *MoxA*) in comparison to four housekeeping genes (*Tub1* JGI PIDs Cf-186859 Ds-68998, *Eif3b* Cf-190521 Ds-75033, *Pap1* Cf-190301 Ds-180959 and *Rps9* Cf-196996 Ds-92035). DNA sequences from *D. septosporum* and *C. fulvum* were aligned with the codon-aware multiple sequence alignment software, RevTrans [135]. Sequence alignments were trimmed in codon units to remove missing data across both species with the sequence editor, Jalview [136]. The non-synonymous/synonymous amino acid ratio (Ka/Ks or ω) was obtained using the Ka/Ks Calculator [137] with the algorithm of Nei and Gojobori [138]. Statistical differences between Ka/Ks values for dothistromin and housekeeping genes were determined using Student's two-sided t test [139]. For determination of dothistromin production, previously published extraction and hplc methods were followed [140].

### Quantitative PCR

For quantification of dothistromin gene expression in *D. septosporum*, RNA was extracted from sporulating lesions on *Pinus radiata* needles collected from a forest in New Zealand (*in planta* sample) or grown in PDB or B5 [141] broths for 6 days as described previously [140]. cDNA synthesis and relative quantitative RT-PCR were carried out using primers and methods described earlier [140], with three biological replicates and two technical replicates. For *C. fulvum*, similar protocols were followed except that tomato infections, RNA extraction and cDNA synthesis followed the protocols of van Esse et al. [142] and four biological replicates were used. Oligonucleotides were designed with Primer3Plus [143] and are shown in Table S14. Their efficiency and specificity were tested on a genomic DNA dilution series. For both species, quantitative PCR was performed with the Applied Biosystems 7300 Real-Time PCR system (Applied Biosystems, USA) using the default parameters. Raw data were analyzed using the 2^−ΔCt^ method [144].

## Supporting Information

Figure S1 Amino acid similarity to *Dothistroma septosporum*. Genome-wide amino acid similarity of homologous proteins between *C. fulvum* and other sequenced fungal species. A pair of proteins is only reported as homologous when the predicted similarity (blastp) spans at least 70% of their lengths and their length difference is at most 20%. Axis indicates number of homologous proteins. Bar shading indicates similarity: red, 91–100%; orange, 81–90%; light green, 71–80%; medium green, 61–70%; turquoise, 51–60%; light blue, 41–50%; dark blue, 31–40%; and purple, 0–30%. Homologous proteins with high amino acid similarity are likely orthologs, whereas for those with lower similarity this relation cannot be inferred. Species abbreviations: Cf, *Cladosporium fulvum*; Sm, *Septoria musiva*; Mf, *Mycosphaerella fijiensis*; Mg, *Mycosphaerella graminicola*; Rr, *Rhytidhysteron rufulum*; Hp, *Hysterium pulicare*; Ab, *Alternaria brassicicola*; Pt, *Pyrenophora tritici-repentis*; Ch, *Cochliobolus heterostrophus*; Sn, *Stagonospora nodorum*; An, *Aspergillus nidulans*; Nc, *Neurospora crassa*; Mo, *Magnaporthe oryzae*; Fg, *Fusarium graminearum*.
(TIF)

Figure S2 Synteny of *Cladosporium fulvum* and *Dothistroma septosporum* genomes. A) Whole-genome synteny, computed using the JGI synteny browser (available at http://genome.jgi-psf.org/Dotse1/Dotse1.home.html) and shown as pairwise alignment blocks. The 14 main *D. septosporum* chromosomes are shown with colored blocks indicating regions of synteny with *C. fulvum*, computed with a 1-kb cut-off. The different colored blocks represent the different *C. fulvum* scaffolds; colour keys were generated independently for the 14 chromosomes and are as shown on the JGI synteny browser. B) Synteny between two examples of large scaffolds of *C. fulvum* (top, Cf2, 526-kb; bottom, Cf5, 315-kb) with corresponding regions of *D. septosporum* chromosomes 2 (3.3-Mb) and 4 (2.6-Mb). In each case the top line (e.g. Cf2) shows blocks indicating regions of synteny with *D. septosporum*, computed with a 50-bp cut-off. Different colors represent matches to different chromosomes, indicating that each *C. fulvum* scaffold predominantly aligns with just one *D. septosporum* chromosome. Slanting lines connecting Cf and Ds scaffolds show matching regions between the two, illustrating that the syntenic blocks are not collinear but show fragmentation and different degrees of dispersal, consistent with intrachromosomal rearrangements.
(PDF)

Figure S3 Hydrophobin genes in Dothideomycete species. Consensus phylogenetic tree of predicted class I and class II hydrophobins from *Cladosporium fulvum* (Cf), *Dothistroma septosporum* (Ds), and *Mycosphaerella graminicola* (Mg). Amino acid sequences of hydrophobins were aligned using ClustalW2 and the phylogenetic tree was constructed using the minimum-evolution method of MEGA5 with 1000 bootstraps. Bootstrap values less than 90% are not shown. Scale bar shows the genetic distance (substitutions per site). The labels (c) and (p) indicate whether expression was detected in culture or *in planta*, respectively, in EST data. Asterisks indicate hydrophobin proteins for which no hydrophobin conserved domain could be identified using InterproScan. However, the phylogenetic tree clearly shows that Ds67650 belongs to class I, and Mg965336 and Cf183780 belong to class II hydrophobins.
(TIF)

Figure S4 Growth profile assays for *Cladosporium fulvum* and *Dothistroma septosporum*. The fungi were grown on 32 solid agar media containing well-defined or complex carbohydrate substrates as detailed at www.fung-growth.org. A) *C. fulvum* grown for 2 weeks and B) *D. septosporum* grown for 4 weeks, both in the dark at 22–25°C.
(TIF)

Figure S5 Synteny of secondary metabolism loci between *Dothistroma septosporum* and *Cladosporium fulvum*. Gene clusters of A) *D. septosporum*-specific and B) *C. fulvum*-specific key genes were predicted based on functional annotations. Synteny of the borders of each gene cluster was checked in the other species. All *D. septosporum*-specific gene clusters are located at conserved loci in *C. fulvum*. Conversely, only 6 *C. fulvum*-specific gene clusters are located at conserved loci in *D. septosporum*. For *CfPks5*, *CfPks6* and *CfNps5*, the border genes are scattered over one or more chromosomes in *D. septosporum*. Black arrows represent key genes; dark grey arrows represent putative accessory genes; light grey arrows represent putative border genes; outlined arrows indicate genes present in only one species; black triangles represent transposable elements. The *C. fulvum* scaffold numbers and *D. septosporum* chromosome numbers are indicated for syntenic loci. Loci are not drawn to scale.
(PDF)

Figure S6 Expression of *Cladosporium fulvum* effector genes *Avr4* and *Avr9* during infection of tomato. Expression of *Avr4* and *Avr9* was measured by quantitative PCR during tomato infection and in two *in vitro* conditions (PDB and B5 media). Expression was calibrated using the tubulin gene according to the 2^−ΔCt^ method [136]. Expression was not detected *in vitro* for *Avr9* and weakly in B5 medium only for *Avr4*. Induction of the expression of both effectors during tomato infection is highlighted by the grey-dashed curve. Relative expression of actin is shown as the control for calibration.
(TIF)

Protocol S1 Genome sequencing, assembly and annotation methods.
(DOC)

Table S1 *Cladosporium fulvum* and *Dothistroma septosporum* sequence statistics.
(DOC)

Table S2 *Dothistroma septosporum* genome scaffolds.
(DOC)

Table S3 Overview of Repeat-Induced Point Mutations (RIP) in *Cladosporium fulvum, Dothistroma septosporum* and other related Dothideomycete fungi. *Neurospora crassa* is used as a reference.
(DOC)

Table S4 Repetitive regions flanking known effectors of *Cladosporium fulvum* and *Dothistroma septosporum*.
(DOC)

Table S5 Comparison of CAZy gene numbers in *Cladosporium fulvum* and *Dothistroma septosporum*.
(DOC)

Table S6 Growth diameters of *Cladosporium fulvum, Dothistroma septosporum* and other fungi on various carbon sources.
(DOC)

Table S7 Putative monoterpene-degrading genes.
(XLS)

Table S8 Comparison of oxidoreductase gene numbers in *Cladosporium fulvum*, *Dothistroma septosporum*, *Mycosphaerella graminicola* and *Stagonospora nodorum*.
(DOC)

Table S9 Key secondary metabolism enzyme identifiers in *Cladosporium fulvum* and *Dothistroma septosporum*.
(DOC)

Table S10 Putative dothistromin genes in *Cladosporium fulvum* and *Dothistroma septosporum*.
(DOC)

Table S11 Regulatory genes involved in development and secondary metabolism of *Cladosporium fulvum* and *Dothistroma septosporum*.
(DOC)

Table S12 Conditions for *Cladosporium fulvum* EST libraries.
(DOC)

Table S13 Conditions for *Dothistroma septosporum* EST libraries.
(DOC)

Table S14 Primers used for reverse transcription quantitative PCR.
(XLS)
