# Correction: The Genomes of the Fungal Plant Pathogens *Cladosporium fulvum* and *Dothistroma septosporum* Reveal Adaptation to Different Hosts and Lifestyles But Also Signatures of Common Ancestry

**DOI:** 10.1371/journal.pgen.1005775

**Published:** 2015-12-22

**Authors:** Pierre J. G. M. de Wit, Ate van der Burgt, Bilal Ökmen, Ioannis Stergiopoulos, Kamel A. Abd-Elsalam, Andrea L. Aerts, Ali H. Bahkali, Henriek G. Beenen, Pranav Chettri, Murray P. Cox, Erwin Datema, Ronald P. de Vries, Braham Dhillon, Austen R. Ganley, Scott A. Griffiths, Yanan Guo, Richard C. Hamelin, Bernard Henrissat, M. Shahjahan Kabir, Mansoor Karimi Jashni, Gert Kema, Sylvia Klaubauf, Alla Lapidus, Anthony Levasseur, Erika Lindquist, Rahim Mehrabi, Robin A. Ohm, Timothy J. Owen, Asaf Salamov, Arne Schwelm, Elio Schijlen, Hui Sun, Harrold A. van den Burg, Roeland C. H. J. van Ham, Shuguang Zhang, Stephen B. Goodwin, Igor V. Grigoriev, Jérôme Collemare, Rosie E. Bradshaw

In S4 Fig, panel B is a duplicate of panel A. Please view the correct S4 Fig here.

## Supporting Information

S4 Fig Growth profile assays for *Cladosporium fulvum* and *Dothistroma septosporum*. The fungi were grown on 32 solid agar media containing well-defined or complex carbohydrate substrates as detailed at www.fung-growth.org. A) *C*. *fulvum* grown for 2 weeks and B) *D*. *septosporum* grown for 4 weeks, both in the dark at 22–25°C.
(PDF)
