# Supplementary figures and images for: The Genomes of the Fungal Plant Pathogens Cladosporium fulvum and Dothistroma septosporum Reveal Adaptation to Different Hosts and Lifestyles But Also Signatures of Common Ancestry
Source: PLoS Genet. 2012 Nov 29;8(11):e1003088. doi: 10.1371/journal.pgen.1003088 (PMC3510045; doi:10.1371/journal.pgen.1003088)

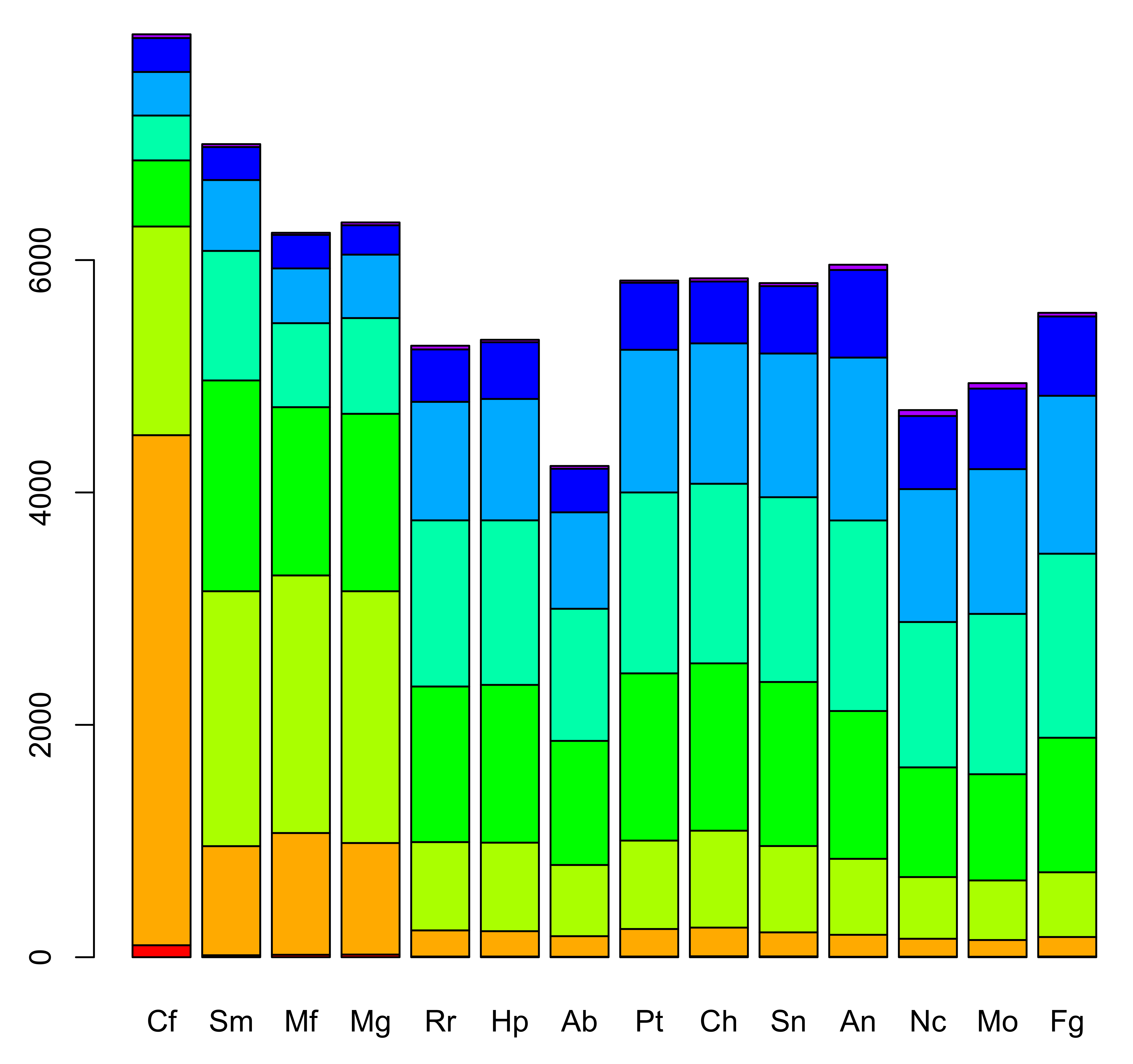

Supplement: Figure S1 — Amino acid similarity to Dothistroma septosporum. Genome-wide amino acid similarity of homologous proteins between C. fulvum and other sequenced fungal species. A pair of proteins is only reported as homologous when the predicted similarity (blastp) spans at least 70% of their lengths and their length difference is at most 20%. Axis indicates number of homologous proteins. Bar shading indicates similarity: red, 91–100%; orange, 81–90%; light green, 71–80%; medium green, 61–70%; turquoise, 51–60%; light blue, 41–50%; dark blue, 31–40%; and purple, 0–30%. Homologous proteins with high amino acid similarity are likely orthologs, whereas for those with lower similarity this relation cannot be inferred. Species abbreviations: Cf, Cladosporium fulvum; Sm, Septoria musiva; Mf, Mycosphaerella fijiensis; Mg, Mycosphaerella graminicola; Rr, Rhytidhysteron rufulum; Hp, Hysterium pulicare; Ab, Alternaria brassicicola; Pt, Pyrenophora tritici-repentis; Ch, Cochliobolus heterostrophus; Sn, Stagonospora nodorum; An, Aspergillus nidulans; Nc, Neurospora crassa; Mo, Magnaporthe oryzae; Fg, Fusarium graminearum. (TIF) [file pgen.1003088.s001.tif]

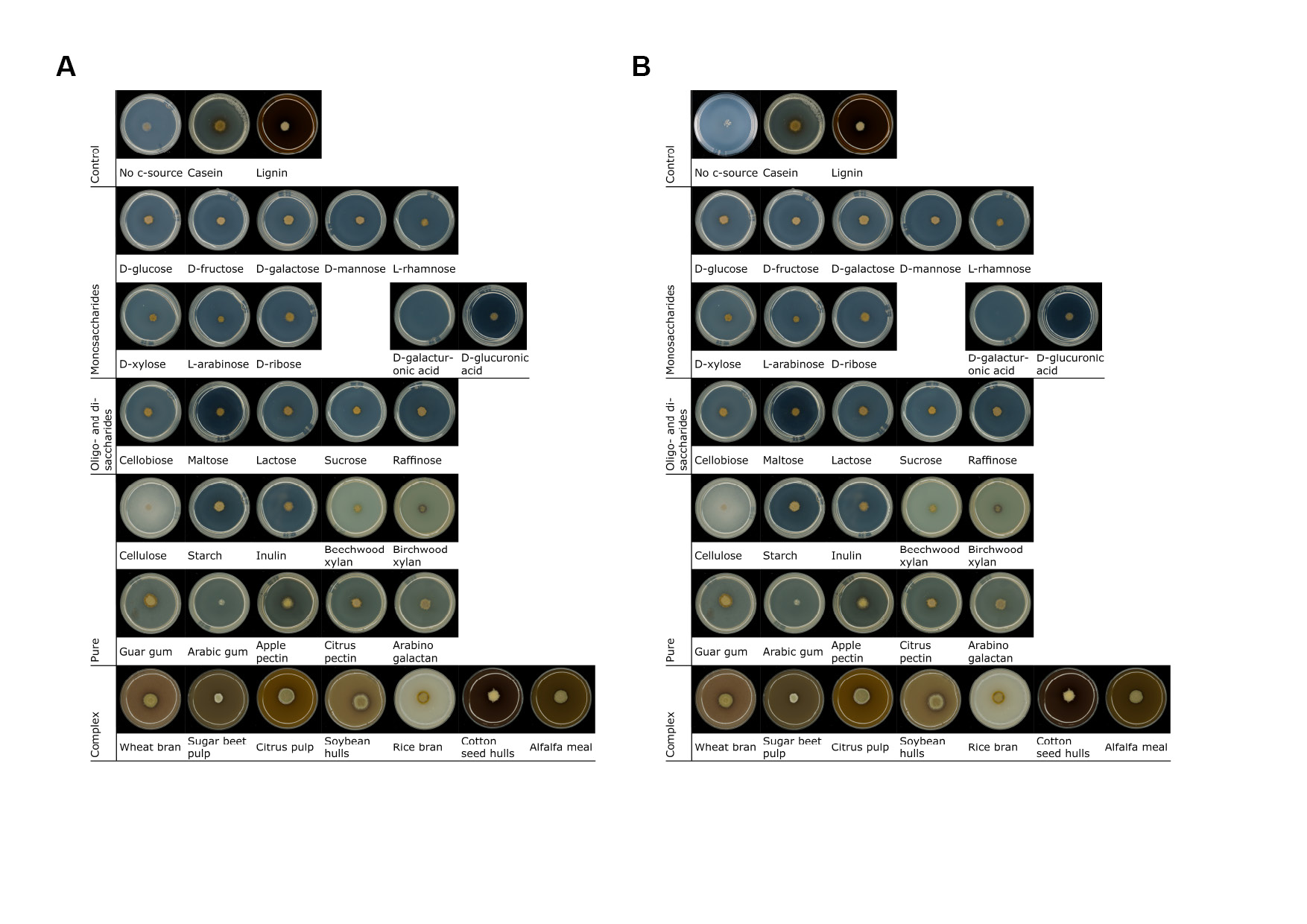

Supplement: Figure S4 — Growth profile assays for Cladosporium fulvum and Dothistroma septosporum. The fungi were grown on 32 solid agar media containing well-defined or complex carbohydrate substrates as detailed at www.fung-growth.org. A) C. fulvum grown for 2 weeks and B) D. septosporum grown for 4 weeks, both in the dark at 22–25°C. (TIF) [file pgen.1003088.s004.tif]

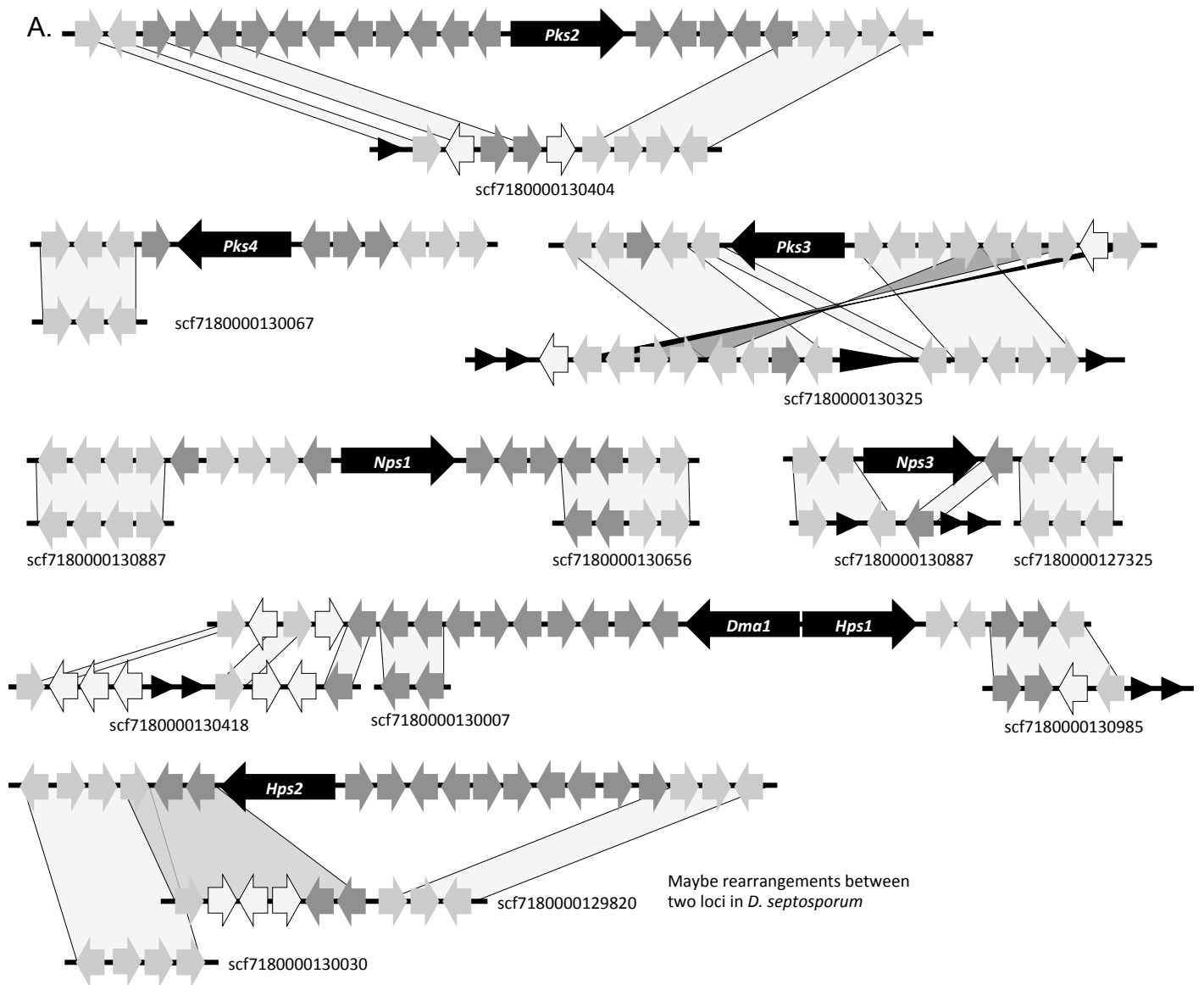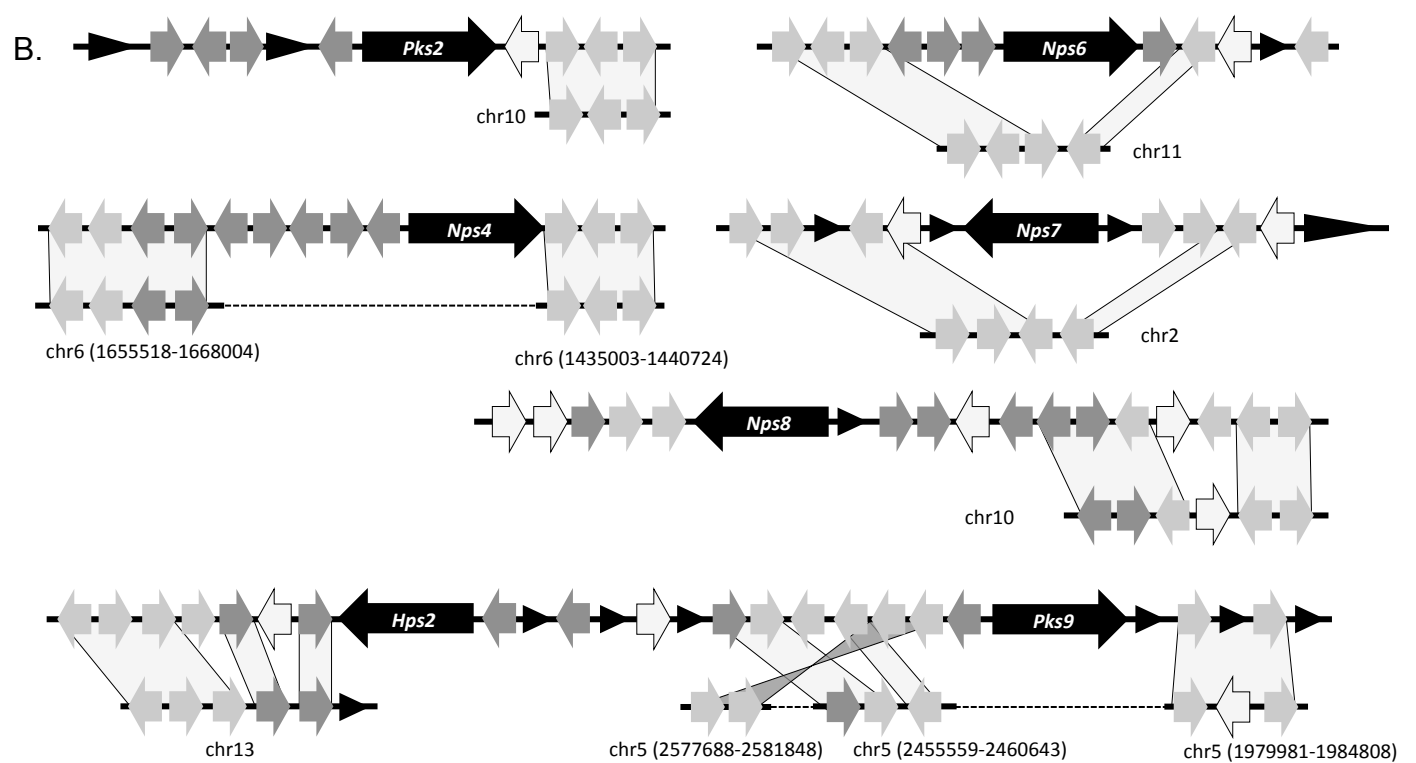

Supplement: Figure S5 — Synteny of secondary metabolism loci between Dothistroma septosporum and Cladosporium fulvum. Gene clusters of A) D. septosporum-specific and B) C. fulvum-specific key genes were predicted based on functional annotations. Synteny of the borders of each gene cluster was checked in the other species. All D. septosporum-specific gene clusters are located at conserved loci in C. fulvum. Conversely, only 6 C. fulvum-specific gene clusters are located at conserved loci in D. septosporum. For CfPks5, CfPks6 and CfNps5, the border genes are scattered over one or more chromosomes in D. septosporum. Black arrows represent key genes; dark grey arrows represent putative accessory genes; light grey arrows represent putative border genes; outlined arrows indicate genes present in only one species; black triangles represent transposable elements. The C. fulvum scaffold numbers and D. septosporum chromosome numbers are indicated for syntenic loci. Loci are not drawn to scale. (PDF) [file pgen.1003088.s005.pdf]

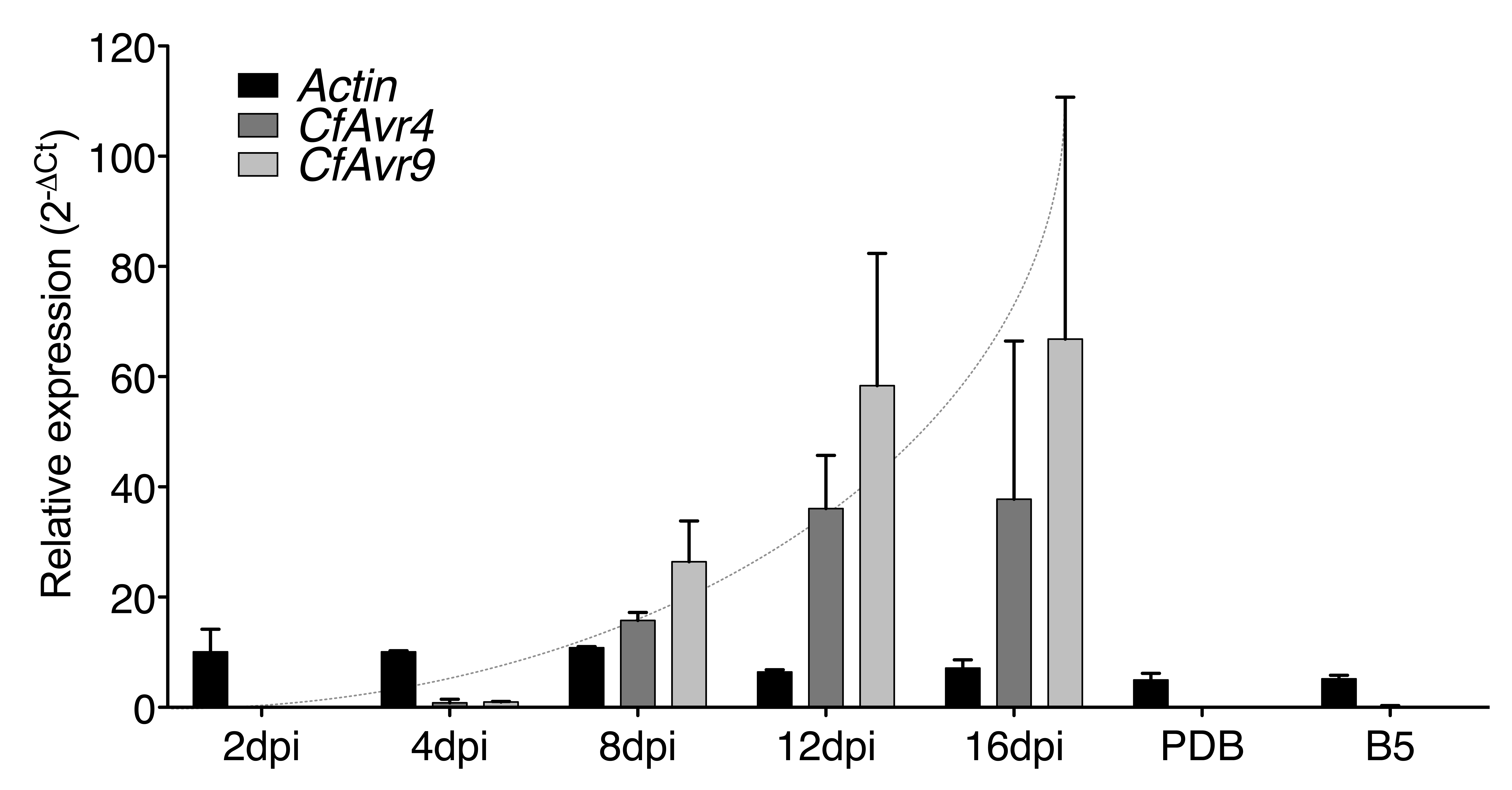

Supplement: Figure S6 — Expression of Cladosporium fulvum effector genes Avr4 and Avr9 during infection of tomato. Expression of Avr4 and Avr9 was measured by quantitative PCR during tomato infection and in two in vitro conditions (PDB and B5 media). Expression was calibrated using the tubulin gene according to the 2−ΔCt method [136]. Expression was not detected in vitro for Avr9 and weakly in B5 medium only for Avr4. Induction of the expression of both effectors during tomato infection is highlighted by the grey-dashed curve. Relative expression of actin is shown as the control for calibration. (TIF) [file pgen.1003088.s006.tif]
