# Supplementary material for: The Genomes of the Fungal Plant Pathogens Cladosporium fulvum and Dothistroma septosporum Reveal Adaptation to Different Hosts and Lifestyles But Also Signatures of Common Ancestry
Source: PLoS Genet. 2012 Nov 29;8(11):e1003088. doi: 10.1371/journal.pgen.1003088 (PMC3510045; doi:10.1371/journal.pgen.1003088)

Synteny of *Cladosporium fulvum* and *Dothistroma septosporum* genomes

A Whole-genome synteny alignment of *C. fulvum* scaffolds with *D. septosporum* chromosomes 1-14

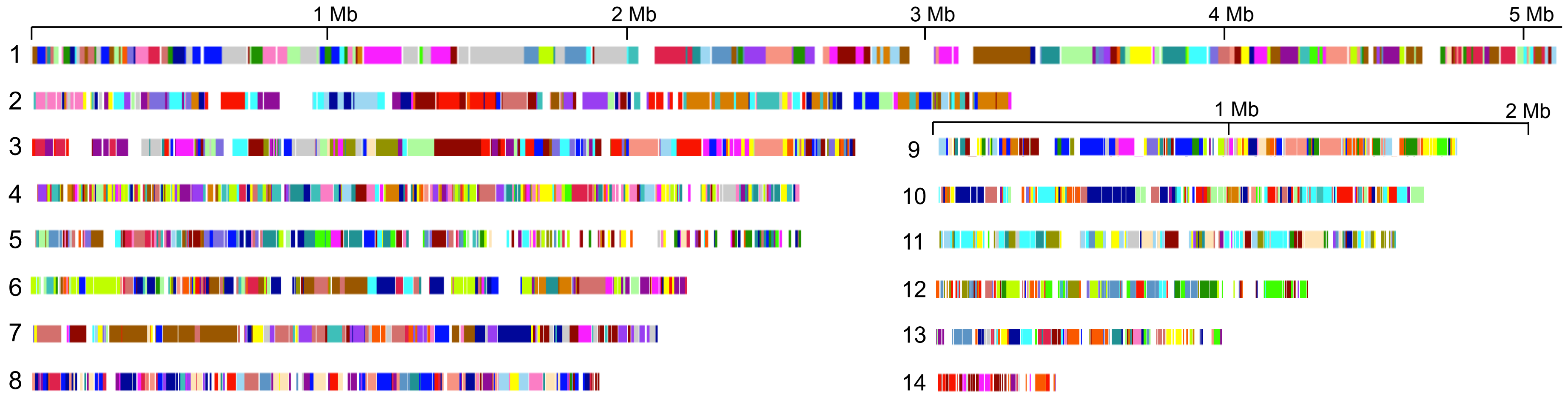

B Synteny between two examples of large scaffolds of *C. fulvum* with corresponding regions of *D. septosporum*

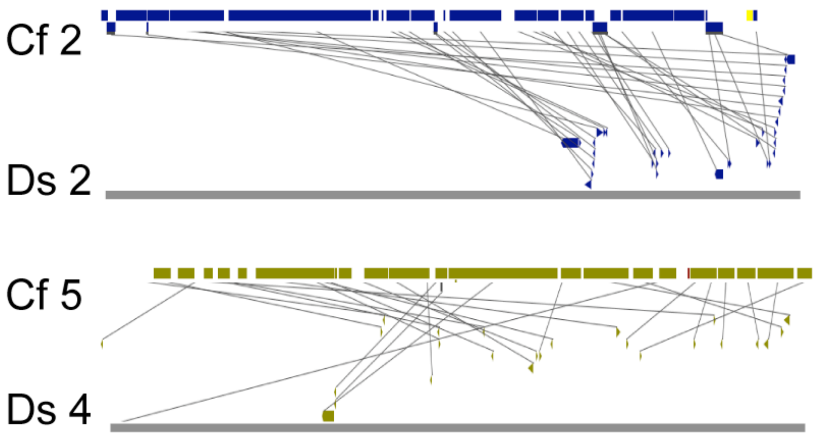

Supplement: Figure S2 — Synteny of Cladosporium fulvum and Dothistroma septosporum genomes. A) Whole-genome synteny, computed using the JGI synteny browser (available at http://genome.jgi-psf.org/Dotse1/Dotse1.home.html) and shown as pairwise alignment blocks. The 14 main D. septosporum chromosomes are shown with colored blocks indicating regions of synteny with C. fulvum, computed with a 1-kb cut-off. The different colored blocks represent the different C. fulvum scaffolds; colour keys were generated independently for the 14 chromosomes and are as shown on the JGI synteny browser. B) Synteny between two examples of large scaffolds of C. fulvum (top, Cf2, 526-kb; bottom, Cf5, 315-kb) with corresponding regions of D. septosporum chromosomes 2 (3.3-Mb) and 4 (2.6-Mb). In each case the top line (e.g. Cf2) shows blocks indicating regions of synteny with D. septosporum, computed with a 50-bp cut-off. Different colors represent matches to different chromosomes, indicating that each C. fulvum scaffold predominantly aligns with just one D. septosporum chromosome. Slanting lines connecting Cf and Ds scaffolds show matching regions between the two, illustrating that the syntenic blocks are not collinear but show fragmentation and different degrees of dispersal, consistent with intrachromosomal rearrangements. (PDF) [file pgen.1003088.s002.pdf]
