# Supplementary material for: The Genomes of the Fungal Plant Pathogens Cladosporium fulvum and Dothistroma septosporum Reveal Adaptation to Different Hosts and Lifestyles But Also Signatures of Common Ancestry
Source: PLoS Genet. 2012 Nov 29;8(11):e1003088. doi: 10.1371/journal.pgen.1003088 (PMC3510045; doi:10.1371/journal.pgen.1003088)
Supplement: Protocol S1 — Genome sequencing, assembly and annotation methods. (DOC) [file pgen.1003088.s007.doc]

**Genome sequencing, assembly and annotation methods**

Fungal DNA and EST library preparation

DNA was isolated from *C. fulvum* mycelia that were scraped from PDA plates using a DNeasy plant mini kit (Qiagen Benelux bv, Venlo, The Netherlands) according to the manufacturer’s instructions and from *D. septosporum* grown in liquid DM (Dothistroma medium) using a modification of the method of Raeder and Broda with Phase Lock Gel tubes (5 PRIME, Hamburg, Germany).

Three EST libraries were produced from *C. fulvum* (Table S12). *C. fulvum* race 0WU was grown under various different conditions that were intended to trigger the expression of as many genes as possible. For the induction EST library of *C. fulvum* (Library 1), the fungus was pre-cultured in 100 ml liquid B5 medium (10^5^ conidia/ml) including vitamins and supplemented with sucrose (2 g/100 ml), at 22 °C and grown in an incubator at 200 rpm for 6 days in order to get sufficient fungal starting material. After 6 days the culture was filtered and transferred to 20 different induction media (Table S12), such as nitrogen limited medium, medium without carbon source, in the presence of H_2_O_2_ or saponins and alkaloids, or at different pH (pH 4, 7 or 9). Progressive starvation EST library (Library 2) was prepared from *C. fulvum* grown in liquid B5 medium and mycelium harvested at 5, 8, 12, 16 and 22 days after incubation at 180 rpm at RT. *In planta* EST library (Library 3) was prepared from tomato Heinz 1706 plants at 4, 6, 9, 12 and 15 days post inoculation with race 0WU. Total RNA was extracted with RNeasy® Plant Mini Kit (Qiagen Benelux bv, Venlo, The Netherlands) according to the manufacturer’s instructions following DNase treatment on the RNA purification column. The cDNAs were prepared using a SMART cDNA Library Construction Kit (Clontech), normalized using a Trimmer kit (Evrogen) according to the manufacturer's instructions, and cloned into pCR2.1 (Invitrogen).

Three EST libraries were also made from *D. septosporum* (Table S13). For the two *in vitro* libraries, RNA was extracted from mycelium grown in either water agar or pine agar/broth (minimal medium prepared with pine-needle soaked water then combined after RNA extraction (CHAA, ‘pine’ library) or in nutrient-rich media including PDA, PDB, V8 agar, DM agar and DM broth (combined as CHAB, ‘rich’ library). The *in planta* EST library was prepared from *Pinus radiata* needles showing late-stage Dothistroma needle blight symptoms (red bands and fruiting bodies) collected from a forest near Rotorua, New Zealand. Total RNA was extracted from *D. septosporum* using TRIZOL (Invitrogen) and Phase Lock Gel tubes (5PRIME) followed by TURBO (Ambion) DNase treatment and precipitation with 2.5 M lithium chloride.

*C. fulvum* genome sequencing and assembly

GS FLX and GS FLX Titanium single stranded shotgun libraries were prepared using 5 μg gDNA fragmented by nebulization to average sizes of 450 bp and 850 bp, respectively, according to manufacturer’s protocols (Roche). A paired-end 454 single stranded DNA library was made using 8.5 μg *C. fulvum* gDNA fragmented to an average length of 3900 bp using a hydroshear (GeneMachines) device. Library construction was performed according to manufacturer’s protocol. HPLC-purified oligonucleotides were annealed in a thermal cycler (Applied Biosystems 9700) to create paired-end adaptors. After purification using ampure beads (Agencourt) *C. fulvum* gDNA fragments were *Eco*RI methylated, end-polished, ligated with hairpin adaptors, cleaved with exonuclease and restriction-digested with *Eco*RI. The resulting fragments were column-purified and yield was measured by fluorescence (PicoGreen, Invitrogen) using a plate reader (Tecan, Fluor scan). Fragments with two *Eco*RI sticky ends were circularized by self-ligation. Double-stranded *C. fulvum* gDNA fragments (4 to 5 kb in size) were used as carrier DNA prior to purification and fragmentation by nebulization. From this step on the library preparation was comparable to the shotgun library preparation including end-polishing, library immobilization using Dynal M-270 streptavidin coated magnetic beads and adaptor ligation. After fill-in of the ligated adaptors, the obtained fragment library was amplified using 15 PCR cycles. This finally resulted in a paired-end 454 single stranded DNA library.

Based on titration sequencing runs clonal amplifications were carried out with ratios of 0.4 and 1.0 DNA fragments to beads for shotgun and paired-end libraries, respectively. After emulsion PCR, amplification beads were recovered and enriched for DNA-carrying beads. Sequencing was performed after loading 900,000 or 2,000,000 beads per region, respectively, on large (70X75) GS FLX or GS FLX Titanium pico titer plates, with all plates divided in two regions. Pyro-sequencing was performed on a 454 Genome Sequencer FLX using 100 flow cycles for GS FLX shotgun and GS FLX paired-end runs whereas 200 flow cycles were used for GS FLX Titanium shotgun sequencing. Initially, six GS FLX shotgun runs and four GS-FLX paired-end runs were carried out. This resulted in 4,639,208 high quality reads with an average read length of 238 bases. Of these, 750,341 reads contained paired-end information derived from 3 kb fragments. Two additional GS-FLX Titanium shotgun runs were carried out resulting in 2,209,539 reads passing all quality filters showing an average read length of 439 bases. Prior to assembly, the 454 reads were divided per chemistry (FLX and Titanium) and subsequently divided into shotgun and matepair reads. The reads were processed as follows, per type without removal of the matepair linker sequence, if present. Duplicate reads, which most likely correspond to (emulsion) PCR duplicates created during library construction, were identified and for each set of duplicates only the longest read was retained. Reads containing more than one ambiguous basecall (N) were discarded, as well as extremely short (<25 flows for FLX chemistry, <50 for Titanium) and extremely long (>250 flows for FLX chemistry, >450 for Titanium) reads. All filtering was performed using custom scripts implemented in Python version 2.6. In total, 20% of the read data were discarded after filtering (Table S1), the majority of which were clonal duplicates.

Reads were assembled with the Celera Assembler version 5.4 using default parameters (Table S1). The assembly resulted in 5,715 contigs that were ordered into 4,865 scaffolds spanning 61.2 Mb, and an additional 26 Mb in 50,572 degenerate contigs that represent unresolved repeat copies with a GC content that deviated somewhat from the remainder of the assembly. In total, 95% of the assembled genome sequence was present in 2,565 scaffolds of 2 kb or larger.

*C. fulvum* EST sequencing and assembly

Twenty five μg total RNA from the three different *C. fulvum* growth conditions; induction, starvation, and *in planta*, were used for normalized, random primed cDNA library preparation suitable for 454 sequencing using sample-specific 6-base-long tag sequences (Vertis Biotechnology AG). The three cDNA libraries consisting of 500 to 700 bp fragments were pooled in equimolar ratio, diluted and used for clonal amplification and subsequent GS FLX Titanium 454 sequencing on one pico titer plate as described above. 1,147,762 high quality reads covering in total 430,029,692 bases were obtained by 454 transcriptome analysis and reads split according to their sample-specific tags then assembled into cDNA contigs and unigenes using Newbler software version 2.5 (*in vitro* induction: 328,374 reads, 12843 unigenes representing 14.9 Mb; *in vitro* starvation: 473,359 reads, 13951 unigenes representing 18.8 Mb; *in planta*: 345,737 reads, 3221 unigenes representing 3.1 Mb).

*C. fulvum* genome annotation and availability

The assembled scaffolds were annotated using the Cyrille2 workflow management system . To reduce the false-positive rate during gene prediction, a *C. fulvum* specific repeat set was generated using the RepeatScout program [44] with default parameters. The identified repeats were annotated using HMMER , and BLASTp searches and subsequently manually curated to remove false positives. Repeats in the scaffold sequences were masked using RepeatMasker with the aforementioned *C. fulvum* repeat set and the RepBase repeat database and RepeatScout . Genes were subsequently predicted on the masked sequences using GeneMark-ES , Augustus and SNAP . Upon comparison of the predicted gene models to the aligned protein sequences of *M. fijiensis* and *M. graminicola*, the GeneMark-ES predictions were selected to represent the *C. fulvum* proteome.

All predicted gene models were functionally annotated using SignalP , TMHMM , InterProScan , BLASTp against GenBank nr, and hardware-accelerated double-affine Smith-Waterman alignments (deCypherSW; www.timelogic.com/decypher_sw.html) against SwissProt (www.expasy.org/sprot), KEGG , and KOG . KEGG hits were used to assign EC numbers (www.expasy.org/enzyme), and Interpro and SwissProt hits were used to map GO terms (www.geneontology.org). Multigene families were predicted with the Markov clustering algorithm MCL to cluster the proteins, using BLASTp alignment scores between proteins as a similarity metric. Functional annotations can be found at the JGI website (www.jgi.doe.gov). This Whole Genome Shotgun project has been deposited at DDBJ/EMBL/GenBank under the accession AMRR00000000. The version described in this paper is the first version, AMRR01000000.

*D. septosporum* genome sequencing and assembly

The *D. septosporum* genome was sequenced using a combination of 454 (Titanium unpaired), Illumina (2x76 bp, 0.3 kb insert paired-end) and Sanger (41 kb insert fosmid) sequencing platforms (Table S1). All general aspects of library construction and sequencing can be found at the JGI website (www.jgi.doe.gov). Two lanes of the Illumina data were assembled with the Velvet assembler (version 0.7.55; with a hash length of 61 and the following options -fastq -cov_cutoff 2 -min_contig_length 100, to produce an assembly with a final graph with 9746 nodes and n50 of 90611, max 423460, total 29985518. Contigs greater than or equal to 800 bp in length were shredded into 1000 bp chunks, if possible, with 800 bp overlap to be used for Newbler assembly. Reverse complemented shreds were also created at contig ends. After eliminating possible contaminant data, the combined set of velvet shredded fragments and 454 reads was assembled with Newbler (version 2.5-internal-10Apr08-1) with the following options; -fe reads2remove.MPA -nrm -info -consed -finish –rip -a 50 -l 350 -g -ml 20 -mi 99 -e 30. This resulted in 20 scaffolds with an N/L50 of 5/2.6 Mb, and 199 contigs with an N/L50 of 31/0.4 Mb and estimated assembled coverage of 34X (Table S1).

*D. septosporum* EST sequencing and assembly

For *in vitro* samples, polyA+ RNA was isolated from total RNA using the absolutely mRNA Purification kit and manufacturer’s instructions (Stratagene, La Jolla, CA). Subsequently, the mRNA was used to construct cDNA libraries using the cDNA Rapid Library Preparation Method (Roche 454 Life Sciences, Branford, CT, USA), followed by sequencing on the 454 GS-FLX platform. Sequencing of libraries CHAA and CHAB resulted in 1,637,800 and 1,414,120 ESTs respectively. Ribosomal RNA, low quality and low complexity reads were filtered out from each set and the 1,083,610 and 509,385 remaining reads were assembled using Newbler (version 2.3-PreRelease-6/30/2009) with default parameters, resulting in 13,184 isotigs (>50 bp long). For *in planta* samples, polyA+ RNA was isolated using magnetic oligo dT beads and cDNA library prepared according to the 2009 Illumina mRNA Sequencing Sample Preparation Guide, followed by sequencing on an Illumina GAIIx. 38,212,684 75-base single-end reads were obtained, of which approximately 11.6% mapped uniquely to the *D. septosporum* genome.

*D. septosporum* genome annotation and availability

The *D. septosporum* genome was annotated using the Joint Genome Institute annotation pipeline (http://www.jgi.doe.gov/). This Whole Genome Shotgun project has been deposited at DDBJ/EMBL/GenBank under the accession AIEN00000000. The version described in this paper is the first version, AIEN01000000.

**References**

1. Raeder U, Broda P (1985) Rapid preparation of DNA from filamentous fungi. Lett Appl Microbiol 1: 17-20.

2. Carsolio C (1994) Characterization of Ech-42, a *Trichoderma harzianum* endochitinase gene expressed during mycoparasitism. Proc Natl Acad Sci U S A 91: 10903-10907.

3. Schwelm A, Barron NJ, Zhang S, Bradshaw RE (2008) Early expression of aflatoxin-like dothistromin genes in the forest pathogen *Dothistroma septosporum*. Mycol Res 112: 138-146.

4. Myers EW, Sutton GG, Delcher AL, Dew IM, Fasulo DP, et al. (2000) A whole-genome assembly of *Drosophila*. Science 287: 2196-2204.

5. Fiers MWEJ, van der Burgt A, Datema E, de Groot JCW, van Ham RCHJ (2008) High-throughput bioinformatics with the Cyrille2 pipeline system. BMC Bioinformatics 9.

6. Melen K, Krogh A, von Heijne G (2003) Reliability measures for membrane protein topology prediction algorithms. J Mol Biol 327: 735-744.

7. Altschul SF, Gish W, Miller W, Myers EW, Lipman DJ (1990) BASIC LOCAL ALIGNMENT SEARCH TOOL. J Mol Biol 215: 403-410.

8. Smit AFA, Hubley R, Green P (1996-2010) RepeatMasker Open-3.0.

9. Jurka J, Kapitonov VV, Pavlicek A, Klonowski P, Kohany O, et al. (2005) Repbase Update, a database of eukaryotic repetitive elements. Cytogenet Genome Res 110: 462–467.

10. Price AL, Jones NC, Pevzner PA (2005) De novo identification of repeat families in large genomes. Bioinformatics 21 Suppl 1: 351-358.

11. Ter-Hovhannisyan V, Lomsadze A, Chernoff YO, Borodovsky M (2008) Gene prediction in novel fungal genomes using an *ab initio* algorithm with unsupervised training. Genome Res 18: 1979-1990.

12. Stanke M, Waack S (2003) Gene prediction with a hidden Markov model and a new intron submodel. Bioinformatics 19: II215-II225.

13. Korf I (2004) Gene finding in novel genomes. BMC Bioinformatics 5.

14. Nielsen H, Engelbrecht J, Brunak S, von Heijne G (1997) Identification of prokaryotic and eukaryotic signal peptides and prediction of their cleavage sites. Protein Eng 10: 1-6.

15. Zdobnov EM, Apweiler R (2001) InterProScan - an integration platform for the signature-recognition methods in InterPro. Bioinformatics 17: 847-848.

16. Kanehisa M, Hattori M, Aoki-Kinoshita KF, Itoh M, Kawashima S, et al. (2006) From genomics to chemical genomics: new developments in KEGG. Genome Biol 5: R7.

17. Koonin EV, Fedorova ND, Jackson JD, Jacobs AR, Krylov DM, et al. (2004) A comprehensive evolutionary classification of proteins encoded in complete eukaryotic genomes. Genome Biol 5: R7.

18. Enright AJ, Van Dongen S, Ouzounis CA (2002) An efficient algorithm for large-scale detection of protein families. Nucleic Acids Research 30: 1575-1584.

19. Zerbino DR, Briney E (2008) Velvet: algorithms for de novo short read assembly using de Bruijn graphs. Genome Res 18: 821-829.

20. Salamov AA, Solovyev VV (2000) Ab initio gene finding in Drosophila genomic DNA. Genome Res 10: 516-522.

21. Birney E, Clamp M, Durbin R (2004) GeneWise and Genomewise. Genome Res 14: 988-995.

22. Kent WJ (2002) BLAT - the BLAST-like alignment tool. Genome Res 12: 656-664.
