# Supplementary material for: The Genomes of the Fungal Plant Pathogens Cladosporium fulvum and Dothistroma septosporum Reveal Adaptation to Different Hosts and Lifestyles But Also Signatures of Common Ancestry
Source: PLoS Genet. 2012 Nov 29;8(11):e1003088. doi: 10.1371/journal.pgen.1003088 (PMC3510045; doi:10.1371/journal.pgen.1003088)
Supplement: Table S1 — Cladosporium fulvum and Dothistroma septosporum sequence statistics. (DOC) [file pgen.1003088.s008.doc]

**Table S1. *Cladosporium fulvum* and *Dothistroma septosporum* sequence statistics**

|  | **Library** | **Library Type** | **Raw Reads** | **Raw Bases** | **Trimmed Bases** | **Assembled Reads** | **Assembled Bases** | **Coverage** | **Insert size, bp** |
| --- | --- | --- | --- | --- | --- | --- | --- | --- | --- |
| ***Cladosporium fulvum*** | A | 454FLX | 3,053,288 | 717,865,558 | 545,297,631 | 1,855,025 | 441,718,496 | 7.27x |  |
|  | B | 454TIT | 2,192,601 | 963,755,636 | 792,642,814 | 1,474,485 | 648,566,068 | 10.67x |  |
|  | C | 454FLX | 1,571,588 | 382,601,325 | 308,506,129 | 1,144,907 | 192,973,089 | 3.17x | 2kb |
|  | **Total** |  | **6,817,477** | **2,064,222,519** | **1,646,446,574** | **4,474,417** | **1,283,257,653** | **21.11x** |  |
| ***Dothistroma septosporum*** | GPOS | Sanger | 69,316 | 62,858,400 | 53,631,693 | 66,118 | 50,704,082 | 1.69x | 41kb |
|  | GSAP | 454 | 1,075,566 | 485,029,662 | 484,833,109 | 1,057,754 | 475,696,807 | 15.82x |  |
|  | GSWB | 454 | 978,564 | 371,069,611 | 370,824,521 | 952,938 | 358,058,185 | 11.91x |  |
|  | NODE | Illumina | 148,633 | 146,488,764 | 146,488,764 | 148,532 | 146,440,524 | 4.87x |  |
|  | **Total** |  | **2,272,079** | **1,065,446,437** | **1,055,778,087** | **2,225,342** | **1,030,899,598** | **34.28x** |  |
