# Supplementary material for: The Genomes of the Fungal Plant Pathogens Cladosporium fulvum and Dothistroma septosporum Reveal Adaptation to Different Hosts and Lifestyles But Also Signatures of Common Ancestry
Source: PLoS Genet. 2012 Nov 29;8(11):e1003088. doi: 10.1371/journal.pgen.1003088 (PMC3510045; doi:10.1371/journal.pgen.1003088)
Supplement: Table S2 — Dothistroma septosporum genome scaffolds. (DOC) [file pgen.1003088.s009.doc]

**Table S2.  *Dothistroma septosporum* genome scaffolds**

| **Scaffold number** | **Length (bp)** | **Predicted chromosome size (Mb)^a^** | **Telomere sequence each end (Yes/no)** | **% in gaps** | **Number of annotated genes** | **Number of genes in *Cf*-syntenic blocks**^b^ | **Ratio *Cf*-syntenic genes to annotated genes** | **Number of syntenic blocks** | **Largest syntenic block (kb)** | **% Syntenic** | **% repetitive sequence** |
| --- | --- | --- | --- | --- | --- | --- | --- | --- | --- | --- | --- |
| 1 | 5111597 | 5.0 | Y  Y | 0.51 | 2148 | 1359 | 0.63 | 160 | 210 | 87.4 | 1.42 |
| 2 | 3306877 | 3.2 | Y  n | 0.61 | 1382 | 829 | 0.60 | 112 | 81 | 81.9 | 1.26 |
| 3 | 2752214 | 2.6 | Y  Y | 0.32 | 1079 | 547 | 0.51 | 98 | 103 | 78.0 | 4.68 |
| 4 | 2620707 | 2.5 | Y  Y | 0.37 | 1060 | 306 | 0.29 | 119 | 53 | 64.2 | 3.16 |
| 5 | 2595548 | 2.5 | Y  Y | 0.39 | 1142 | 250 | 0.22 | 93 | 46 | 48.8 | 2.48 |
| 6 | 2187679 | 2.1 | Y  Y | 0.40 | 925 | 402 | 0.43 | 82 | 87 | 72.7 | 5.35 |
| 7 | 2106062 | 2.1 | Y  Y | 0.50 | 846 | 429 | 0.51 | 75 | 76 | 77.6 | 4.82 |
| 8 | 1927254 | 2.0 | Y  Y | 0.32 | 745 | 288 | 0.39 | 75 | 46 | 71.1 | 1.97 |
| 9 | 1757826 | 1.8 | Y  Y | 0.39 | 861 | 338 | 0.39 | 66 | 75 | 70.2 | 3.94 |
| 10 | 1635767 | 1.6 | Y  Y | 0.47 | 681 | 264 | 0.39 | 65 | 35 | 68.1 | 3.93 |
| 11 | 1558309 | 1.5 | Y  n | 0.76 | 679 | 251 | 0.37 | 57 | 40 | 67.8 | 4.71 |
| 12 | 1256034 | 1.3 | Y  Y | 0.31 | 509 | 196 | 0.39 | 45 | 47 | 61.1 | 5.45 |
| 13 | 962570 | 1.0 | Y  Y | 0.49 | 418 | 157 | 0.38 | 37 | 38 | 64.3 | 2.90 |
| 14 | 407968 | ND^C^ | Y  n | 0.14 | 164 | 52 | 0.32 | 18 | 24 | 60.9 | 1.79 |

^a^Sizes predicted from pulsed-field gel electrophoresis .

^b^Syntenic blocks are defined as outlined in Material and Methods.

^c^Not Determined (due to lack of resolution).

1. Bradshaw RE, Jin HP, Morgan BS, Schwelm A, Teddy OR, et al. (2006) A polyketide synthase gene required for biosynthesis of the aflatoxin-like toxin, dothistromin. Mycopathologia 161: 283-294.
