# Supplementary material for: The Genomes of the Fungal Plant Pathogens Cladosporium fulvum and Dothistroma septosporum Reveal Adaptation to Different Hosts and Lifestyles But Also Signatures of Common Ancestry
Source: PLoS Genet. 2012 Nov 29;8(11):e1003088. doi: 10.1371/journal.pgen.1003088 (PMC3510045; doi:10.1371/journal.pgen.1003088)
Supplement: Table S3 — Overview of Repeat-Induced Point Mutations (RIP) in Cladosporium fulvum, Dothistroma septosporum and other related Dothideomycete fungi. Neurospora crassa is used as a reference. (DOC) [file pgen.1003088.s010.doc]

**Table S3. Overview of Repeat-Induced Point Mutations (RIP) in *Cladosporium fulvum, Dothistroma septosporum* and other related Dothideomycete fungi. *Neurospora crassa* and *Leptosphaeria maculans* are used as a reference.**

|  | ***C. fulvum*** | | ***D. septosporum*** | | ***M. graminicola*** | | ***S. nodorum*** | | ***N. crassa*** | | ***L. maculans*** | |
| --- | --- | --- | --- | --- | --- | --- | --- | --- | --- | --- | --- | --- |
| Repeat sequence ≥500nt | 27,170 kb | 44.5%^a^ | 798 kb | 2.6%^a^ | 6,121 kb | 15.4%^a^ | 2,430 kb | 6.5%^a^ | 2,004 kb | 5.1%^a^ | 15,219 kb | 33.9%^a^ |
| Repeat loci ≥500nt | 7101 |  | 133 |  | 967 |  | 405 |  | 653 |  | 1230 |  |
| RIP’d sequence | 25,882 kb | 42.4%^a^ | 1,114 kb | 3.7%^a^ | 7,236 kb | 18.2%^a^ | 2,504 kb | 6.7%^a^ | 2,361 kb | 6.0%^a^ | 15,628 kb | 34.8%^a^ |
| RIP’d loci | 5447 |  | 65 |  | 1331 |  | 421 |  | 596 |  | 1182 |  |
| Repeated sequence ≥500nt with RIP signature | 26,397 kb | 97.2%^b^ | 782 kb | 98.0%^b^ | 5,991 kb | 97.9%^b^ | 2,360 kb | 97.2%^b^ | 1,402 kb | 69.9%^b^ | 15,194 kb | 99.8%^b^ |
| Repeat loci ≥500nt with RIP signature | 6506 | 91.6%^c^ | 114 | 85.7%^c^ | 890 | 92.0%^c^ | 381 | 94.1%^c^ | 358 | 54.8%^c^ | 1198 | 97.4%^c^ |
| Repeat loci ≥500nt without RIP signature | 595 | 8.4%^c^ | 19 | 14.3%^c^ | 77 | 8.0%^c^ | 24 | 5.9%^c^ | 295 | 45.2%^c^ | 32 | 2.6%^c^ |
| RIP’d loci not assigned as repeat | 27 | 0.5%^d^ | 11 | 16.9%^d^ | 173 | 13.0%^d^ | 26 | 6.2%^d^ | 210 | 35.2%^d^ | 61 | 5.2%^d^ |

^a^ percentage of the genome; ^b^ percentage of repeated sequence ≥500nt; ^c^ percentage of repeat loci ≥500nt; ^d^ percentage of RIP’d loci
