# Supplementary material for: The Genomes of the Fungal Plant Pathogens Cladosporium fulvum and Dothistroma septosporum Reveal Adaptation to Different Hosts and Lifestyles But Also Signatures of Common Ancestry
Source: PLoS Genet. 2012 Nov 29;8(11):e1003088. doi: 10.1371/journal.pgen.1003088 (PMC3510045; doi:10.1371/journal.pgen.1003088)
Supplement: Table S4 — Repetitive regions flanking known effectors of Cladosporium fulvum and Dothistroma septosporum. (DOC) [file pgen.1003088.s011.doc]

**Table S4. Repetitive regions flanking known effectors of *Cladosporium fulvum* (*Cf*) and *Dothistroma septosporum* (*Ds*)**

| **Gene name and JGI protein ID** | **Scaffold number** | **Gene position** | **Direction fwd/rev** | **Repeat position** | **Repeat size** | **Repeat type** |
| --- | --- | --- | --- | --- | --- | --- |
| CfAvr2 | scf7180000130334 | 174089..174376 | fwd | [172831..173186](http://www.ab.wur.nl/cgi-bin/gbrowse/clado_12_cabog?name=scf7180000130334:172831..173186) | 355 | Retrotransposon00021 |
| No gene |  |  |  | [173187..173788](http://www.ab.wur.nl/cgi-bin/gbrowse/clado_12_cabog?name=scf7180000130334:173187..173788) | 601 | Unknown00134 |
| annotation |  |  |  | [173788..174020](http://www.ab.wur.nl/cgi-bin/gbrowse/clado_12_cabog?name=scf7180000130334:173788..174020) | 232 | Retrotransposon00021 |
| CfAvr4 | scf7180000130296 | 14866..14459 | rev | [20351..21488](http://www.ab.wur.nl/cgi-bin/gbrowse/clado_12_cabog?name=scf7180000130296:20351..21488) | 1137 | Unknown00048 |
| 189855 |  |  |  | [21554..22407](http://www.ab.wur.nl/cgi-bin/gbrowse/clado_12_cabog?name=scf7180000130296:21554..22407) | 853 | Copia00004 |
|  |  |  |  | [22441..26579](http://www.ab.wur.nl/cgi-bin/gbrowse/clado_12_cabog?name=scf7180000130296:22441..26579) | 4138 | Retrotransposon00021 |
|  |  |  |  | [26675..27046](http://www.ab.wur.nl/cgi-bin/gbrowse/clado_12_cabog?name=scf7180000130296:26675..27046) | 371 | Retrotransposon00021 |
|  |  |  |  | [27050..27932](http://www.ab.wur.nl/cgi-bin/gbrowse/clado_12_cabog?name=scf7180000130296:27050..27932) | 882 | Unknown00048 |
|  |  |  |  | [27931..31267](http://www.ab.wur.nl/cgi-bin/gbrowse/clado_12_cabog?name=scf7180000130296:27931..31267) | 3336 | Copia |
|  |  |  |  | [31238..34286](http://www.ab.wur.nl/cgi-bin/gbrowse/clado_12_cabog?name=scf7180000130296:31238..34286) | 3048 | Retrotransposon00028 |
|  |  |  |  | [34286..35268](http://www.ab.wur.nl/cgi-bin/gbrowse/clado_12_cabog?name=scf7180000130296:34286..35268) | 982 | Unknown00052 |
|  |  |  |  | [35268..36214](http://www.ab.wur.nl/cgi-bin/gbrowse/clado_12_cabog?name=scf7180000130296:35268..36214) | 946 | Retrotransposon00020 |
|  |  |  |  | [36210..36854](http://www.ab.wur.nl/cgi-bin/gbrowse/clado_12_cabog?name=scf7180000130296:36210..36854) | 644 | Copia00005 |
| CfAvr4E | scf7180000126296 | 5317..5682 | fwd | [7429..8224](http://www.ab.wur.nl/cgi-bin/gbrowse/clado_12_cabog?name=scf7180000126296:7429..8224) | 795 | Unknown00048 |
| 183814 |  |  |  | [6225..7360](http://www.ab.wur.nl/cgi-bin/gbrowse/clado_12_cabog?name=scf7180000126296:6225..7360) | 1135 | Unknown00048 |
|  |  |  |  | 300..4000 | 3700 | Retrotransposon00019 |
|  |  |  |  | 1..299 | 298 | Unknown00020 |
| CfAvr9 | scf7180000130851 | 12970..12720 | rev | [6900..11782](http://www.ab.wur.nl/cgi-bin/gbrowse/clado_12_cabog?name=scf7180000130851:6900..11782) | 4882 | Copia00002 |
| No gene |  |  |  | [6078..6901](http://www.ab.wur.nl/cgi-bin/gbrowse/clado_12_cabog?name=scf7180000130851:6078..6901) | 823 | Unknown00111 |
| annotation |  |  |  | 1500..6700 | 5200 | Copia00004 |
|  |  |  |  | 570..1270 | 700 | Unknown00052 |
|  |  |  |  | 1..445 | 444 | Retrotransposon00001 |
| CfEcp1 | scf7180000126735 | 11769..12213 | fwd | [12844..13385](http://www.ab.wur.nl/cgi-bin/gbrowse/clado_12_cabog?name=scf7180000126735:12844..13385) | 541 | Unknown00048 |
| 184167 |  |  |  | [9333..10722](http://www.ab.wur.nl/cgi-bin/gbrowse/clado_12_cabog?name=scf7180000126735:9333..10722) | 1389 | Retrotransposon00038 |
|  |  |  |  | [7981..9336](http://www.ab.wur.nl/cgi-bin/gbrowse/clado_12_cabog?name=scf7180000126735:7981..9336) | 1355 | Unknown00032 |
|  |  |  |  | [3665..7148](http://www.ab.wur.nl/cgi-bin/gbrowse/clado_12_cabog?name=scf7180000126735:3665..7148) | 3483 | Retrotransposon00021 |
|  |  |  |  | [2689..3542](http://www.ab.wur.nl/cgi-bin/gbrowse/clado_12_cabog?name=scf7180000126735:2689..3542) | 853 | Unknown00020 |
|  |  |  |  | 503..1445 | 942 | Retrotransposon00008 |
|  |  |  |  | 2..502 | 500 | Unknown00052 |
| CfEcp2-1 | scf7180000130979 | 8402..7849 | rev | [47850..48263](http://www.ab.wur.nl/cgi-bin/gbrowse/clado_12_cabog?name=scf7180000130979:47850..48263) | 413 | Retrotransposon00025 |
| 197200 |  |  |  | [42691..47438](http://www.ab.wur.nl/cgi-bin/gbrowse/clado_12_cabog?name=scf7180000130979:42691..47438) | 4747 | Retrotransposon00007 |
|  |  |  |  | [41585..42708](http://www.ab.wur.nl/cgi-bin/gbrowse/clado_12_cabog?name=scf7180000130979:41585..42708) | 1123 | Retrotransposon00030 |
|  |  |  |  | [35740..41359](http://www.ab.wur.nl/cgi-bin/gbrowse/clado_12_cabog?name=scf7180000130979:35740..41359) | 5619 | Retrotransposon00005 |
|  |  |  |  | [29062..35753](http://www.ab.wur.nl/cgi-bin/gbrowse/clado_12_cabog?name=scf7180000130979:29062..35753) | 6691 | Gypsy00006 |
|  |  |  |  | [28578..29095](http://www.ab.wur.nl/cgi-bin/gbrowse/clado_12_cabog?name=scf7180000130979:28578..29095) | 517 | Gypsy00004 |
|  |  |  |  | [27628..28578](http://www.ab.wur.nl/cgi-bin/gbrowse/clado_12_cabog?name=scf7180000130979:27628..28578) | 950 | Retrotransposon00005 |
|  |  |  |  | 26426..27630 | 1204 | Retrotransposon00021 |
|  |  |  |  | [2081..2821](http://www.ab.wur.nl/cgi-bin/gbrowse/clado_12_cabog?name=scf7180000130979:2081..2821) | 740 | Unknown00021 |
|  |  |  |  | [1642..2085](http://www.ab.wur.nl/cgi-bin/gbrowse/clado_12_cabog?name=scf7180000130979:1642..2085) | 443 | Retrotransposon00006 |
|  |  |  |  | 1..1640 | 1639 | Copia00001 |
| CfEcp2-2 | scf180000130842 | 19702..20877 | fwd | 1..412 | 411 | Retrotransposon00025 |
| 195482 |  |  |  | 406..1860 | 1454 | Unknown00020 |
|  |  |  |  | 1854..2886 | 1032 | Retrotransposon00030 |
|  |  |  |  | 116937..117679 | 742 | LINE00003 |
| CfEcp2-3 | scf7180000130692 | 74368.. 74848 | fwd | 1..2961 | 2960 | Copia00001 |
| 193474 |  |  |  | 2959..3369 | 410 | LINE00003 |
|  |  |  |  | 3365..3869 | 504 | Unknown00134 |
|  |  |  |  | 94099..94465 | 366 | Unknown00174 |
|  |  |  |  | 127600..135000 | 7400 | Gypsy0004/1 |
|  |  |  |  | 135000..138350 | 3350 | Unknown |
| CfEcp4 | scf718000012883 | 41393..40977 | rev | 3555[..5087](http://www.ab.wur.nl/cgi-bin/gbrowse/clado_12_cabog?name=scf7180000128839:4440..5087) | 1532 | Unknown00018 |
| 186834 |  |  |  | [1681..2697](http://www.ab.wur.nl/cgi-bin/gbrowse/clado_12_cabog?name=scf7180000128839:1681..2697) | 1016 | LINE00001 |
|  |  |  |  | 1..1687 | 1686 | Retrotransposon00030 |
| CfEcp5 | scf7180000130562 | 4403..5148 | fwd | [7313..7930](http://www.ab.wur.nl/cgi-bin/gbrowse/clado_12_cabog?name=scf7180000130562:7313..7930) | 617 | Retrotransposon00021 |
| No gene annotation |  |  |  | [2229..4130](http://www.ab.wur.nl/cgi-bin/gbrowse/clado_12_cabog?name=scf7180000130562:2229..4130) | 1901 | Unknown00018 |
|  |  |  |  | 1..2010 | 2009 | Retrotransposon00014 |
| CfEcp6 | scf7180000130247 | 40667.. 39820 | rev | 1..1327 | 1326 | Retrotransposon00021 |
| 189398 |  |  |  | [1324..8223](http://www.ab.wur.nl/cgi-bin/gbrowse/clado_12_cabog?name=scf7180000130247:5229..8223) | 6899 | Gypsy00001 |
|  |  |  |  | [8313..9076](http://www.ab.wur.nl/cgi-bin/gbrowse/clado_12_cabog?name=scf7180000130247:8313..9076) | 763 | Unknown00016 |
|  |  |  |  | [77361..80312](http://www.ab.wur.nl/cgi-bin/gbrowse/clado_12_cabog?name=scf7180000130247:78212..80312) | 2951 | Unknown00015/17 |
| CfEcp7 | scf7180000130885 | 72088..71738 | rev | 1..5678 | 5677 | Retrotransposon00030/7 |
| No gene |  |  |  | [5767..6903](http://www.ab.wur.nl/cgi-bin/gbrowse/clado_12_cabog?name=scf7180000130885:5767..6903) | 1136 | Unknown00048 |
| annotation |  |  |  | [6902..11762](http://www.ab.wur.nl/cgi-bin/gbrowse/clado_12_cabog?name=scf7180000130885:6902..11762) | 4860 | Copia00004 |
|  |  |  |  | [11756..14450](http://www.ab.wur.nl/cgi-bin/gbrowse/clado_12_cabog?name=scf7180000130885:11756..14450) | 2694 | Retrotransposon00017 |
|  |  |  |  | [14450..15542](http://www.ab.wur.nl/cgi-bin/gbrowse/clado_12_cabog?name=scf7180000130885:14450..15542) | 1092 | Unknown00052 |
|  |  |  |  | [15529..16141](http://www.ab.wur.nl/cgi-bin/gbrowse/clado_12_cabog?name=scf7180000130885:15529..16141) | 612 | Copia00004 |
|  |  |  |  | [16142..16682](http://www.ab.wur.nl/cgi-bin/gbrowse/clado_12_cabog?name=scf7180000130885:16142..16682) | 540 | Unknown00038 |
|  |  |  |  | [30955..32971](http://www.ab.wur.nl/cgi-bin/gbrowse/clado_12_cabog?name=scf7180000130885:30955..32089) | 2016 | Unknown00021 |
|  |  |  |  | [75708..76845](http://www.ab.wur.nl/cgi-bin/gbrowse/clado_12_cabog?name=scf7180000130885:75708..76845) | 1137 | Unknown00048 |
|  |  |  |  | [77726..81906](http://www.ab.wur.nl/cgi-bin/gbrowse/clado_12_cabog?name=scf7180000130885:77726..81906) | 4180 | Copia00003 |
|  |  |  |  | [81902..82349](http://www.ab.wur.nl/cgi-bin/gbrowse/clado_12_cabog?name=scf7180000130885:81902..82349) | 447 | Copia00002 |

| **Gene name and JGI protein ID** | **Scaffold number** | **Gene position** | **Direction fwd/rev** | **Repeat position** | **Repeat size** | **Repeat type** |
| --- | --- | --- | --- | --- | --- | --- |
| DsAvr4 | sc_8 | 384698..385195 | fwd | 265709..265982 | 273 | Unknown |
| 36707 |  |  |  | 266619..266844 | 225 | Unknown |
|  |  |  |  | 268341..269589 | 1248 | LTR retrotransposon |
|  |  |  |  | 269598..270121 | 523 | Unknown |
|  |  |  |  | 270135..270476 | 341 | Unknown |
|  |  |  |  | 270487..271265 | 778 | Unknown |
|  |  |  |  | 271266..272646 | 1380 | LTR retrotransposon |
|  |  |  |  | 272649..273859 | 1210 | LTR retrotransposon |
|  |  |  |  | 273864..274387 | 523 | Solo-LTR |
|  |  |  |  | 274397..275182 | 785 | Unknown |
|  |  |  |  | 275605..285125 | 9520 | Ty3-gypsy |
|  |  |  |  | 283126..287511 | 4385 | Ty3-gypsy |
|  |  |  |  | 287499..298272 | 10773 | Ty3-gypsy |
|  |  |  |  | 298362..299329 | 967 | Unknown |
| DsEcp2-1 | sc_10 | 215524..216247 | rev | 200681..200999 | 318 | MITE |
| 158381 |  |  |  | 255020..255539 | 519 | Solo-LTR |
|  |  |  |  | 255543..261770 | 6227 | Ty1-copia |
|  |  |  |  | 261775..262294 | 519 | Solo-LTR |
|  |  |  |  | 263025..266791 | 3766 | Ty3-gypsy |
|  |  |  |  | 266794..267805 | 1011 | Unknown |
|  |  |  |  | 267811..269773 | 1962 | Ty3-gypsy |
|  |  |  |  | 269772..283070 | 13298 | LTR retrotransposon |
|  |  |  |  | 282714..283118 | 404 | LTR retrotransposon |
|  |  |  |  | 283168..283384 | 216 | Ty3-gypsy |
|  |  |  |  | 283384..283589 | 205 | Ty1-copia |
|  |  |  |  | 283574..284204 | 630 | Ty1-copia |
|  |  |  |  | 283580..284136 | 556 | Ty1-copia |
|  |  |  |  | 284205..284418 | 213 | Unknown |
|  |  |  |  | 284670..286915 | 2245 | Ty3-gypsy |
|  |  |  |  | 286946..287470 | 524 | Solo-LTR |
|  |  |  |  | 287471..288454 | 983 | Ty3-gypsy |
| DsEcp2-2 | sc_4 | 2241857..2242851 | rev | 2173799..2197290 | 23491 | Helitron-like |
| 127671 |  |  |  | 2198517..2199631 | 1114 | Ty1-copia |
|  |  |  |  | 2198677..2199631 | 954 | Ty1-copia |
|  |  |  |  | 2199864..2200107 | 243 | Ty3-gypsy |
|  |  |  |  | 2200409..2201259 | 850 | Ty3-gypsy |
|  |  |  |  | 2200415..2200943 | 528 | Ty3-gypsy |
|  |  |  |  | 2201257..2201660 | 403 | LTR retrotransposon |
|  |  |  |  | 2201301..2208429 | 7128 | LTR retrotransposon |
|  |  |  |  | 2208417..2208939 | 522 | Solo-LTR |
|  |  |  |  | 2208943..2213523 | 4580 | Ty1-copia |
|  |  |  |  | 2213527..2214497 | 970 | Ty1-copia |
|  |  |  |  | 2214499..2215020 | 521 | Solo-LTR |
|  |  |  |  | 2215020..2221252 | 6232 | LTR retrotransposon |
|  |  |  |  | 2221251..2222417 | 1166 | Ty3-gypsy |
|  |  |  |  | 2221288..2223284 | 1996 | Ty3-gypsy |
|  |  |  |  | 2223283..2236548 | 13265 | LTR retrotransposon |
|  |  |  |  | 2236195..2236673 | 478 | LTR retrotransposon |
|  |  |  |  | 2236195..2236839 | 644 | LTR retrotransposon |
| DsEcp2-3 | sc_4 | 1398479..1400737 | fwd | 1253254..1253612 | 358 | Ty3-gypsy |
| 23431 |  |  |  | 1253494..1263757 | 10263 | Ty3-gypsy |
|  |  |  |  | 1263934..1265861 | 1927 | Ty3-gypsy |
|  |  |  |  | 1263943..1265469 | 1526 | Ty3-gypsy |
|  |  |  |  | 1265833..1266042 | 209 | Ty3-gypsy |
|  |  |  |  | 1265890..1266094 | 204 | Ty3-gypsy |
|  |  |  |  | 1265926..1266211 | 285 | Ty3-gypsy |
|  |  |  |  | 1595341..1595542 | 201 | Unknown |
| DsEcp4 | sc_1 | 4798997..4799409 | fwd | 4711941..4712363 | 422 | LTR retrotransposon |
| 192200 |  |  |  | 4712006..4712363 | 357 | LTR retrotransposon |
|  |  |  |  | 4712363..4719052 | 6689 | Helitron-like |
|  |  |  |  | 4718786..4719110 | 324 | Helitron-like |
|  |  |  |  | 4720643..4720948 | 305 | Unknown |
|  |  |  |  | 4723716..4724517 | 801 | Unknown |
|  |  |  |  | 4723719..4724435 | 716 | Unknown |
|  |  |  |  | 4724518..4724845 | 327 | Ty1-copia |
|  |  |  |  | 4724970..4725491 | 521 | Solo-LTR |
|  |  |  |  | 4725269..4725892 | 623 | Unknown |
|  |  |  |  | 4725494..4726164 | 670 | Unknown |
|  |  |  |  | 4726394..4726959 | 565 | Ty1-copia |
|  |  |  |  | 4726960..4727974 | 1014 | Unknown |
|  |  |  |  | 4729166..4729432 | 266 | Ty1-copia |
|  |  |  |  | 4731871..4732112 | 241 | Unknown |
|  |  |  |  | 4732137..4732616 | 479 | Unknown |
|  |  |  |  | 4732620..4733059 | 439 | Unknown |
| DsEcp5  (pseudogene) | sc_2 | 1483406..1483849 | fwd |  |  |  |
| DsEcp6 | sc_8 | 112019..113076 | fwd | 233792..234362 | 570 | Unknown |
| 46236 |  |  |  | 265709..265982 | 273 | Unknown |
|  |  |  |  | 266619..266844 | 225 | Unknown |
|  |  |  |  | 268341..269589 | 1248 | LTR retrotransposon |
|  |  |  |  | 269598..270121 | 523 | Unknown |
|  |  |  |  | 270135..270476 | 341 | Unknown |
