# Supplementary material for: The Genomes of the Fungal Plant Pathogens Cladosporium fulvum and Dothistroma septosporum Reveal Adaptation to Different Hosts and Lifestyles But Also Signatures of Common Ancestry
Source: PLoS Genet. 2012 Nov 29;8(11):e1003088. doi: 10.1371/journal.pgen.1003088 (PMC3510045; doi:10.1371/journal.pgen.1003088)
Supplement: Table S5 — Comparison of CAZy gene numbers in Cladosporium fulvum and Dothistroma septosporum. (DOC) [file pgen.1003088.s012.doc]

**Table S5. Comparison of CAZy gene numbers in *C. fulvum* (*Cf*) and *D. septosporum* (*Ds*)**

| **Predicted function^a^** | **CAZy family^b^** | ***Cf*** | ***Ds*** | **Predicted function** | **CAZy family^a^** | ***Cf*** | ***Ds*** |
| --- | --- | --- | --- | --- | --- | --- | --- |
| PCW-C | GH6 | 0 | 0 | PCW | CBM1 | 0 | 1 |
| PCW-C | GH7 | 2 | 1 | PCW | CBM6 | 0 | 0 |
| PCW-C | GH12 | 4 | 4 | PCW | CBM13 | 1 | 0 |
| PCW-C | GH45 | 1 | 1 | PCW | CBM35 | 2 | 1 |
| PCW-C | GH61 | 2 | 2 | PCW | CBM42 | 1 | 1 |
| PCW-C | GH74 | 0 | 0 | PCW | CBM63 | 1 | 1 |
| PCW-C | GH94 | 0 | 0 | FCW | CBM14 | 1 | 1 |
| PCW-H | GH10 | 2 | 1 | FCW | CBM18 | 7 | 6 |
| PCW-H | GH11 | 2 | 2 | FCW | CBM24 |  |  |
| PCW-H | GH26 | 0 | 0 | FCW | CBM43 | 1 | 1 |
| PCW-H | GH27 | 1 | 2 | FCW | CBM52 | 1 | 0 |
| PCW-H | GH29 | 1 | 1 | energy | CBM20 | 4 | 4 |
| PCW-H | GH31 | 15 | 10 | energy | CBM21 | 1 | 1 |
| PCW-H | GH35 | 6 | 3 | energy | CBM48 | 3 | 3 |
| PCW-H | GH36 | 2 | 2 |  | CBM32 | 1 | 1 |
| PCW-H | GH39 | 2 | 0 |  | CBM50 | 4 | 3 |
| PCW-H | GH67 | 1 | 1 |  | **Total CBM** | **28** | **24** |
| PCW-HP | GH43 | 22 | 11 | PCW-H | CE1 | 5 | 2 |
| PCW-HP | GH51 | 2 | 1 | PCW-H | CE2 | 0 | 0 |
| PCW-HP | GH53 | 2 | 1 | PCW-H | CE3 | 3 | 2 |
| PCW-HP | GH54 | 1 | 1 | PCW-H | CE5 | 11 | 4 |
| PCW-HP | GH62 | 0 | 1 | PCW-H | CE15 | 0 | 0 |
| PCW-HP | GH93 | 2 | 1 | PCW-H | CE16 | 7 | 5 |
| PCW-pectin | GH28 | 15 | 4 | PCW-HP | CE12 | 2 | 2 |
| PCW-pectin | GH78 | 6 | 1 | PCW-pectin | CE8 | 2 | 3 |
| PCW-pectin | GH88 | 2 | 0 | FCW | CE4 | 3 | 4 |
| PCW-pectin | GH95 | 2 | 0 | Other | CE9 | 2 | 1 |
| PCW-pectin | GH105 | 4 | 3 |  | **Total CE** | **35** | **23** |
| PCW-pectin | GH115 | 1 | 1 |  | GT1 | 6 | 5 |
| PCW/FCW | GH1 | 3 | 2 |  | GT2 | 18 | 15 |
| PCW/FCW | GH2 | 5 | 4 |  | GT3 | 1 | 1 |
| PCW/FCW | GH3 | 19 | 12 |  | GT4 | 5 | 5 |
| PCW/FCW | GH5 | 16 | 12 |  | GT5 | 2 | 2 |
| FCW | GH16 | 16 | 16 |  | GT8 | 10 | 11 |
| FCW | GH17 | 8 | 8 |  | GT15 | 3 | 4 |
| FCW | GH18 | 13 | 10 |  | GT17 | 3 | 3 |
| FCW | GH20 | 2 | 2 |  | GT20 | 3 | 3 |
| FCW | GH55 | 5 | 4 |  | GT21 | 1 | 1 |
| FCW | GH64 | 5 | 6 |  | GT22 | 4 | 4 |
| FCW | GH71 | 2 | 1 |  | GT24 | 1 | 1 |
| FCW | GH72 | 8 | 7 |  | GT25 | 2 | 6 |
| FCW | GH75 | 1 | 1 |  | GT31 | 9 | 6 |
| FCW | GH76 | 8 | 8 |  | GT32 | 5 | 7 |
| FCW | GH81 | 1 | 1 |  | GT33 | 1 | 1 |
| FCW | GH92 | 7 | 8 |  | GT34 | 6 | 5 |
| Energy | GH13 | 15 | 13 |  | GT35 | 1 | 1 |
| Energy | GH15 | 2 | 2 |  | GT39 | 3 | 3 |
| Energy | GH32 | 4 | 2 |  | GT41 | 1 | 1 |
| Energy | GH37 | 2 | 1 |  | GT48 | 1 | 1 |
| Energy | GH65 | 1 | 1 |  | GT50 | 1 | 1 |
|  | GH24 | 2 | 1 |  | GT57 | 2 | 2 |
|  | GH25 | 0 | 1 |  | GT58 | 1 | 1 |
|  | GH30 | 2 | 2 |  | GT59 | 0 | 1 |
|  | GH38 | 1 | 1 |  | GT62 | 3 | 3 |
|  | GH42 | 1 | 1 |  | GT66 | 1 | 1 |
|  | GH47 | 8 | 8 |  | GT69 | 2 | 2 |
|  | GH63 | 1 | 1 |  | GT71 | 4 | 4 |
|  | GH79 | 6 | 4 |  | GT76 | 0 | 1 |
|  | GH114 | 1 | 1 |  | GT90 | 5 | 7 |
|  | GH125 | 3 | 2 |  | **Total GT** | **105** | **110** |
|  | **GH127** | **1** | **0** | PCW-pectin | PL1 | 3 | 1 |
|  | **GH128** | **5** | **3** | PCW-pectin | PL3 | 3 | 0 |
|  | **Total GH** | **268** | **198** | PCW-pectin | PL4 | 2 | 2 |
| **^a^**Predicted family functions assigned according to Amselem J et al. (2011) Genomic analysis of the necrotrophic fungal pathogens *Sclerotinia sclerotiorum* and *Botrytis cinerea*. PLoS Genetics 7:e1002230. | | | | PCW-pectin | PL9 | 0 | 0 |
|  |  |  |  | PCW-pectin | PL11 | 0 | 0 |
|  |  |  |  |  | PL7 | 1 | 0 |
|  |  |  |  |  | PL14 | 0 | 1 |
|  |  |  |  |  | **Total PL** | **9** | **3** |

^b^Families defined in the Carbohydrate-active enzymes database [[www.cazy.org](http://www.cazy.org/); Cantarel BL et al. (2009) The Carbohydrate-Active EnZymes database (CAZy): an expert resource for Glycogenomics. Nucl Acids Res 37: D233-238
