# Supplementary material for: The Genomes of the Fungal Plant Pathogens Cladosporium fulvum and Dothistroma septosporum Reveal Adaptation to Different Hosts and Lifestyles But Also Signatures of Common Ancestry
Source: PLoS Genet. 2012 Nov 29;8(11):e1003088. doi: 10.1371/journal.pgen.1003088 (PMC3510045; doi:10.1371/journal.pgen.1003088)
Supplement: Table S6 — Growth diameters of Cladosporium fulvum, Dothistroma septosporum and other fungi on various carbon sources. (DOC) [file pgen.1003088.s013.doc]

**Table S6. Colony diameters of *Cladosporium fulvum, Dothistroma septosporum* and other fungi cultured on various carbon sources.**

| (diameter in cm) | Cf | Ds | Mg | Sn | Mo | Ss |
| --- | --- | --- | --- | --- | --- | --- |
| No C | 0.5 | 0.3 | 0.4 | 0.7 | ­0.6 | 0 |
| No C | 0.5 | 0.3 | 0.4 | 0.6 | 0.6 | 0 |
| D-glucose | 0.7 | 0.7 | 0.4 | 0.7 | 1.7 | 2.2 |
| D-glucose | 0.6 | 0.7 | 0.4 | 0.7 | 1.7 | 2.3 |
| D-xylose | 0.5 | 0.4 | 0.4 | 0.6 | 1.2 | 0.1 |
| D-xylose | 0.5 | 0.4 | 0.4 | 0.6 | 1.2 | 0.1 |
| D-arabinose | 0.4 | 0.3 | 0.4 | 0.6 | 1.3 | 0.1 |
| D-arabinose | 0.4 | 0.3 | 0.4 | 0.5 | 1.3 | 0.1 |
| Sucrose | 0.5 | 0.7 | 0.3 | 1.1 | 1.7 | 0.8 |
| Sucrose | 0.5 | 0.6 | 0.3 | 1.1 | 1.7 | 0.8 |
| Inulin | 0.6 | 0.3 | 0.4 | 0.9 | 1.9 | 0.1 |
| Inulin | 0.6 | 0.3 | 0.4 | 0.9 | 1.8 | 0.1 |
| Birchwood xylan | 0.7 | 0.3 | 0.3 | 0.5 | 1.7 | 0 |
| Birchwood xylan | 0.7 | 0.3 | 0.3 | 0.5 | 1.8 | 0 |
| Apple pectin | 0.8 | 1.2 | 0.6 | 1.0 | 1.8 | 3.2 |
| Apple pectin | 0.8 | 1.2 | 0.7 | 1.0 | 1.8 | 3.2 |
| Lignin | 0.6 | 0.6 | 0.3 | 0.8 | 0.7 | 0.1 |
| Lignin | 0.6 | 0.7 | 0.3 | 0.9 | 0.8 | 0.1 |

*Cf*, *Cladopsorium fulvum*; *Ds*, *Dothistroma septosporum*; *Mg,* *Mycosphaerella graminicola*; *Sn*, *Septoria nodorum*; *Mo*, *Magnaporthe oryzae*; *Ss*, *Sclerotinia sclerotiorum*

D-glucose, D-xylose, L-arabinose and sucrose were added at a final concentration of 25 mM. Inulin, birchwood xylan, apple pectin and lignin were added at a final concentration of 1% (w/v). Numbers represent diameters from duplicate plates.
