# Supplementary material for: The Genomes of the Fungal Plant Pathogens Cladosporium fulvum and Dothistroma septosporum Reveal Adaptation to Different Hosts and Lifestyles But Also Signatures of Common Ancestry
Source: PLoS Genet. 2012 Nov 29;8(11):e1003088. doi: 10.1371/journal.pgen.1003088 (PMC3510045; doi:10.1371/journal.pgen.1003088)
Supplement: Table S8 — Comparison of oxidoreductase gene numbers in Cladosporium fulvum, Dothistroma septosporum, Mycosphaerella graminicola and Stagonospora nodorum. (DOC) [file pgen.1003088.s015.doc]

**Table S8. Comparison of oxidoreductase gene numbers in *Cladosporium fulvum* (*Cf*), *Dothistroma septosporum* (*Ds*), *Mycosphaerella graminicola* (*Mg*) and *Stagonospora nodorum* (*Sn*).**

| Module code^a^ | Putative function | *Cf* | *Ds* | *Mg* | *Sn* | Eastwood 2011 categories^b^ | Sn from Eastwood 2011 |
| --- | --- | --- | --- | --- | --- | --- | --- |
| AA1_1 | Laccase^c^ | 5/10^d^ | 3/9 | 2/5 | 1/8 | Multicopper oxidases | 8 |
| AA1_2 | Ferroxidase | 1/1 | 1/2 | 1/1 | 1/1 | ND^e^ | ND |
| AA2 | Lignin or manganese or versatile peroxidase | 0/0 | 0/1 | 0/1 | 0/4 | Class II peroxidase | 5 |
| AA3_1 | Cellobiose dehydrogenase | 2/2 | 2/2 | 1/1^f^ | 3/4 ^f^ | Cellobiose dehydrogenase | 3 |
| AA3_2 | Aryl-alcohol oxidase | 0/5 | 0/5 | 0/4 | 0/2 | Aryl-alcohol oxidase | 2 |
| AA3_2 | Glucose oxidase | 0/1 | 1/1 | 0/0 | 1/2 | Glucose oxidase | 2 |
| AA3_3 | Alcohol oxidase | 3/4 | 2/2 | 2/2 | 3/3 | Alcohol oxidase | 3 |
| AA4 | Vanillyl-alcohol oxidase | 0/0 | 1/1 | 0/0 | 0/1 | ND | ND |
| AA5_1 | Glyoxal oxidase, copper radical oxidase | 2/2 | 2/2 | 0/0 | 2/2 | Glyoxal oxidase | 1 |
| AA5_2 | Galactose oxidase | 1/2 | 1/1 | 1/1 | 3/3 | ND | ND |
| AA6 | Quinone reductase | 1/1 | 1/1 | 1/1 | 1/1 | Quinone reductase | 1 |
| AA7 | Glucooligosaccharide oxidase | 0/0 | 0/0 | 0/0 | 1/3 | ND | ND |
| AA8 | Iron reductase domain | 3/3 | 3/3 | 2/4 | 9/9 | ND | ND |

^a^ Sequence-based family definitions for oxidoreductase enzyme modules as determined by Henrissat and Levasseur (unpublished). Families AA1, AA3 and AA5 are subdivided into sequence-based subfamilies denoted AA1_1, AA1_2, AA3_1, AA3_2, AA3_3, AA3_4, AA5_1 and AA5_2.

^b^ To enable comparison with oxidoreductase analyses of Eastwood et al (2011), equivalent categories are indicated, along with numbers of those genes recorded for *Stagonospora nodorum* (*Sn*) (Hane et al. 2007).

^c^ One putative laccase gene from each species also has a carbohydrate-binding domain (CBM20).

^d^ n/m : the first number represents the close relative(s), i.e. those whose function can be reliably inferred while the second number is the total number (close plus distant relatives)

^e^ ND: not determined.

^f^ One also has an iron reductase domain and carbohydrate-binding domain (CBM1).

**References**

Eastwood DC, Floudas D, Binder M, Majcherczyk A, Schneider P, et al. (2011) The plant cell wall-decomposing machinery underlies the functional diversity of forest fungi. Science 333: 762-765.

Hane JK, Lowe RGT, Solomon PS, Tan KC, Schoch CL, et al. (2007) Dothideomycete-plant interactions illuminated by genome sequencing and EST analysis of the wheat pathogen *Stagonospora nodorum*. Plant Cell 19: 3347-3368.
