# Supplementary material for: The Genomes of the Fungal Plant Pathogens Cladosporium fulvum and Dothistroma septosporum Reveal Adaptation to Different Hosts and Lifestyles But Also Signatures of Common Ancestry
Source: PLoS Genet. 2012 Nov 29;8(11):e1003088. doi: 10.1371/journal.pgen.1003088 (PMC3510045; doi:10.1371/journal.pgen.1003088)
Supplement: Table S9 — Key secondary metabolism enzyme identifiers in Cladosporium fulvum and Dothistroma septosporum. (DOC) [file pgen.1003088.s016.doc]

**Table S9. Key secondary metabolism enzyme identifiers in *Cladosporium fulvum* and *Dothistroma septosporum.***

| **Classification** | ***Cladosporium***  ***fulvum*** | ***Dothistroma septosporum*** |
| --- | --- | --- |
| PKS^a^ | **Pks1: 191425**  Pks2: 186350  Pks3: 188474  Pks4: 188483  Pks5: 184292  Pks6: 184395  Pks7: 196875  Pks8: 196070  Pks9: 188153  **PksA: 194256** | **Pks1: 47338**  Pks2: 73814  Pks3: 90367  Pks4: 24654  **PksA: 48345** |
| NRPS^b^ | Nps1: 185841  **Nps2: 193954**  Nps3: 186614  Nps4: 192008  Nps5: 191780/191781  Nps6: 189598  Nps7: 196498/196499/196500/196501  Nps8: 191358  Nps9: 190730  Nps10: 196066/196067/196068 | Nps1: 52251  **Nps2: 90481**  Nps3: 71189 |
| Hybrid PKS-NRPS | Hps1: 192259  Hps2: 188140/188141/188142 | Hps1: 180045  Hps2: 157678 |
| DMATS^c^ | Dma1: 191785 | Dma1: 28625 |

Numbers are JGI protein identification numbers for

*C. fulvum* (<http://genome.jgi-psf.org/Clafu1/Clafu1.home.html>) and

*D. septosporum* (<http://genome.jgi.doe.gov/Dotse1/Dotse1.home.html>).

Genes shown in bold are considered to be involved in biosynthetic pathways shared between the two species, based on similarity and conserved synteny of associated cluster genes.

^a^Polyketide Synthase.

^b^Non-Ribosomal Peptide Synthetase.

^c^DiMethylAllyl Tryptophan Synthase.
