# Supplementary material for: The Genomes of the Fungal Plant Pathogens Cladosporium fulvum and Dothistroma septosporum Reveal Adaptation to Different Hosts and Lifestyles But Also Signatures of Common Ancestry
Source: PLoS Genet. 2012 Nov 29;8(11):e1003088. doi: 10.1371/journal.pgen.1003088 (PMC3510045; doi:10.1371/journal.pgen.1003088)
Supplement: Table S10 — Putative dothistromin genes in Cladosporium fulvum and Dothistroma septosporum. (DOC) [file pgen.1003088.s017.doc]

**Table S10. Putative dothistromin genes in *Dothistroma septosporum* and *Cladosporium fulvum.***

| Dothistromin gene name | Predicted *A. flavus* ortholog | *D. septosporum* Protein ID^a^ | *C. fulvum* Protein ID^a^ | *Ds-Cf* % amino acid identity |
| --- | --- | --- | --- | --- |
| *HexA* | *aflA* | 66976 | 194433 | 85.5 |
| *HexB* | *aflB* | 181128 | 194434 ^b^ | 86.6 |
| *PksA* | *aflC* | 192192 | 194256 | 86.5 |
| *Nor1* | *aflD* | 75691 | 194456 | 86.6 |
| *AvnA* | *aflG* | 57312 | 194434^b^ | 88.9 |
| *AdhA* | *aflH* | 48495 | 194457 | 88.6 |
| *AvfA* | *aflI* | 75546 | 194254 | 74.3 |
| *Est1* | *aflJ* | 75609 | 194417 | 81.1 |
| *VbsA* | *aflK* | 75656 | 194430 | 94.4 |
| *VerB* | *aflL* | 75692 | 194459 | 93.3 |
| *Ver1^c^* | *aflM* | 192193 | 197005 | 97.7 |
| *AflR* | *aflR* | 75566 | 197014 | 83.6 |
| *AflJ* | *aflS* | 57214 | 197013 | 49 |
| *DotC* | *aflT* | 75413 | 194202 | 86.2 |
| *CypX* | *aflV* | 139960 | 194255 | 89.6 |
| *MoxY* | *aflW* | 75547 | 194253 | 90.9 |
| *OrdB* | *aflX* | 75648 | 194437 | 94.0 |
| *HypC* | *aflZ* | 75655 | 194431 | 80.0 |

^a^Protein identification (accession) numbers refer to those at (<http://genome.jgi.doe.gov/Dotse1/Dotse1.home.html>) for *D. septosporum* and

(<http://genome.jgi-psf.org/Clafu1/Clafu1.home.html>) for *C. fulvum*.

^b^*Cf-AvnA* was not annotated separately from the adjacent gene *Cf-HexB* thus both are numbered 194434

^c^Previously called *dotA*.
