# Supplementary material for: The Genomes of the Fungal Plant Pathogens Cladosporium fulvum and Dothistroma septosporum Reveal Adaptation to Different Hosts and Lifestyles But Also Signatures of Common Ancestry
Source: PLoS Genet. 2012 Nov 29;8(11):e1003088. doi: 10.1371/journal.pgen.1003088 (PMC3510045; doi:10.1371/journal.pgen.1003088)
Supplement: Table S11 — Regulatory genes involved in development and secondary metabolism of Cladosporium fulvum and Dothistroma septosporum. (DOC) [file pgen.1003088.s018.doc]

**Table S11. Regulatory genes involved in development and secondary metabolism of *Cladosporium fulvum* (*Cf*) and *Dothistroma septosporum* (*Ds*).**

| **Protein** | **Pathway** | ***Cf***  **protein ID** | **e-value** | ***Ds* protein ID** | **e-value** | **Query** | **Ref** |
| --- | --- | --- | --- | --- | --- | --- | --- |
| PPT | 4’- phosphopantetheinyl transferase | 196722 | 1E-40 | 70926 | 1.16E-36 | XP_755193.1 |  |
| LaeA | Histone methyl transferase | 186126 | 3E-28 | 148869 | 2.03E-20 | AAR01218.1 |  |
| HdaA | Histone deacetylase | 192224 | 1E-111 | 69309 | 1.2E-130 | XP_748144.1 |  |
| RcoA (Tup1) | Chromatin-associated repressor | 191985 | 1E-178 | 72346 | 0.0 | ACD46267.1 |  |
| VeA | Light-dependent regulator | 188542 | 4E-63 | 69562 | 8.45E-64 | AAS07022.1 |  |
| VelB | Light-dependent regulator | 184120 | 3E-65 | 139168 | 1.31E-64 | ABQ17967.1 |  |
| FphA | Red light phytochrome-like receptor | 186523 | 0.0 | 121759 | 0.0 | CAI30283.1 |  |
| WC1-LreA | Blue light sensor | 187550 | 1E-141 | 74976 | 4.2E-139 | AAP47230.1 |  |
| WC2-LreB | Blue light sensor | 186128 | 2E-56 | 70543 | 7.29E-61 | AAP47576.1 |  |
| FadA | G-protein alpha subunit | 184993 | 0.0 | 68770 | 0.0 | AAC49476.1 |  |
| GpgA | G-protein gamma subunit | 194037 | 3E-10 | 67735 | 9.25E-31 | ABG73391.1 |  |
| SfaD | G-protein beta subunit | 186471 | 1E-137 | 69818 | 0.0 | AAC33436.1 |  |
| FlbA | Regulator of G-proteins | 189643 | 0.89 | 72196 | 5.0E-162 | Ds: AAA73955.1  Cf: 72196 |  |
| PhnA | Phosducin regulator of G-proteins | 186472 | 5E-38 | 69817 | 7.63E-45 | XP_657686.1 |  |
| RgsA | Regulator of G-protein | 189643 | 0.088 | 72181 | 6.3E-42 | CBF81270.1 |  |
| PkaA | cAMP-dep. kinase regulatory subunit | 189651 | 1E-127 | 70102 | 8.7E-91 | CBF69742.1 |  |
| PkaC | cAMP-dep. kinase catalytic subunit | 191615 | 1E-167 | 70835 | 0.0 | AAF75762.1 |  |
| PkcA | Protein kinase C | 187240 | 0.0 | 178140 | 0.0 | BAD02338.1 |  |
| StuA | Transcription factor | 194867 | 1E-102 | 45737 | 1.52E-92 | AAA33325.2 |  |
| mpkB | MAP kinase | 189973 | 0.0 | 73345 | 0.0 | ABL85066.1 |  |
| AreA | Transcription factor | 193392 | 1E-109 | 41021 | 4.5E-110 | CAA36731.1 |  |
| CreA | Transcription factor | 195369 | 2E-45 | 71762 | 1.51E-82 | AAR02858.1 |  |
| PacC | Transcription factor | 189565 | 3E-92 | 68527 | 6.81E-86 | CAA87390.1 |  |
| HapB | CCAAT-binding tetrameric transcription factor | 192369 | 7E-37 | 68866 | 2.63E-40 | CAA74100.2 |  |
| HapC | CCAAT-binding tetrameric transcription factor | 190694 | 1E-34 | 71840 | 1.77E-56 | AAC49411.1 |  |
| HapE | CCAAT-binding tetrameric transcription factor | 189467 | 2E-71 | 72361 | 8.21E-74 | AAD12363.1 |  |
| PpoA | Fatty acid oxygenase (oxylipins) | 197623 | 0.0 | 67567 | 0.0 | AAR88626.1 |  |
| PpoB | Fatty acid oxygenase (oxylipins) | No hit | - | No hit | - | AAX35769.1 |  |
| PpoC | Fatty acid oxygenase (oxylipins) | 197370 | 1E-131 | 119905 | 2.0E-113 | AAT36614.1 |  |
| CsnE | COP9 subunit 5 (regulates Ppo) | 188488 | 1E-114 | 69442 | 7.1E-124 | AAM95164.1 |  |
| Cand1 | cullin RING ubiquitin ligases inhibitor | 196436 | 0.0 | 69750 | 0.0 | XP_660062.1 |  |
| StrA | ER striatin | 195525 | 1E-148 | 72705 | 0.0 | XP_681340.1 |  |
| HXK1 | Hexokinase | 194815 | 0.0 | 73129 | 0.0 | ABY89285.1 |  |

**References**

1. Marquez-Fernandez O, Trigos A, Ramos-Balderas JL, Viniegra-Gonzalez G, Deising HB, et al. (2007) Phosphopantetheinyl transferase CfwA/NpgA is required for *Aspergillus nidulans* secondary metabolism and asexual development. Euk Cell 6: 710-720.

2. Bok JW, Keller NP (2004) LaeA, a regulator of secondary metabolism in *Aspergillus* spp. Eukaryotic Cell 3: 527-535.

3. Lee I, Oh JH, Shwab EK, Dagenais TRT, Andes D, et al. (2009) HdaA, a class 2 histone deacetylase of Aspergillus fumigatus, affects germination and secondary metabolite production. Fung Genet Biol 46: 782-790.

4. Hicks J, Lockington RA, Strauss J, Dieringer D, Kubicek CP, et al. (2001) RcoA has pleiotropic effects on *Aspergillus nidulans* cellular development. Mol Microbiol 39: 1482-1493.

5. Calvo AM, Bok J, Brooks W, Keller NP (2004) VeA is required for toxin and sclerotial production in *Aspergillus parasiticus*. Appl Environ Microbiol 70: 4733-4739.

6. Bayram O, Krappmann S, Ni M, Bok JW, Helmstaedt K, et al. (2008) VelB/VeA/LaeA complex coordinates light signal with fungal development and secondary metabolism. Science 320: 1504-1506.

7. Blumenstein A, Vienken K, Tasler R, Purschwitz J, Veith D, et al. (2005) The *Aspergillus nidulans* phytochrome FphA represses sexual development in red light. Curr Biol 15: 1833-1838.

8. Atoui A, Kastner C, Larey CM, Thokala R, Etxebeste O, et al. (2010) Cross-talk between light and glucose regulation controls toxin production and morphogenesis in *Aspergillus nidulans*. Fung Genet Biol.

9. Hicks JK, Yu J-H, Keller NP, Adams TH (1997) *Aspergillus* sporulation and mycotoxin production both require inactivation of the FadA Ga protein-dependent signaling pathway. EMBO J 16: 4916-4923.

10. Seo JA, Han KH, Yu JH (2005) Multiple roles of a heterotrimeric G-protein gamma-subunit in governing growth and development of *Aspergillus nidulans*. Genetics 171: 81-89.

11. Rosen S, Yu JH, Adams TH (1999) The *Aspergillus nidulans sfaD* gene encodes a G protein beta subunit that is required for normal growth and repression of sporulation. EMBO J 18: 5592-5600.

12. Yu JH, Rosen S, Adams TH (1999) Extragenic suppressors of loss-of-function mutations in the *Aspergillus* FlbA regulator of G-protein signaling domain protein. Genetics 151: 97-105.

13. Seo JA, Yu JH (2006) The phosducin-like protein PhnA is required for G beta gamma-mediated signaling for vegetative growth, developmental control, and toxin biosynthesis in *Aspergillus nidulans*. Euk Cell 5: 400-410.

14. Han KH, Seo JA, Yu JH (2004) Regulators of G-protein signalling in *Aspergillus nidulans*: RgsA downregulates stress response and stimulates asexual sporulation through attenuation of GanB (G alpha) signalling. Mol Microbiol 53: 529-540.

15. Shimizu K, Keller NP (2001) Genetic involvement of a cAMP-dependent protein kinase in a g protein signaling pathway regulating morphological and chemical transitions in *Aspergillus nidulans*. Genetics 157: 591-600.

16. Herrmann M, Sprote P, Brakhage AA (2006) Protein kinase C (PkcA) of *Aspergillus nidulans* is involved in penicillin production. Appl Environ Microbiol 72: 2957-2970.

17. Twumasi-Boateng K, Yu Y, Chen D, Gravelat FN, Nierman WC, et al. (2009) Transcriptional profiling identifies a role for BrlA in the response to nitrogen depletion and for StuA in the regulation of secondary metabolite clusters in *Aspergillus fumigatus*. Euk Cell 8: 104-115.

18. Atoui A, Bao D, Kaur N, Grayburn WS, Calvo AM (2008) *Aspergillus nidulans* natural product biosynthesis is regulated by mpkB, a putative pheromone response mitogen-activated protein kinase. Appl Environ Microbiol 74: 3596-3600.

19. Cary JW, Ehrlich KC, Kale SP, Calvo AM, Bhatnagar D, et al. (2006) Regulatory elements in aflatoxin biosynthesis. Mycotoxin Res 22: 105-109.

20. Espeso EA, Penalva MA (1992) Carbon catabolite repression can account for the temporal pattern of expression of a penicillin biosynthetic gene in *Aspergillus nidulans*. Mol Microbiol 6: 1457-1465.

21. Ehrlich KC, Cary JW, Montalbano BG (1999) Characterization of the promoter for the gene encoding the aflatoxin biosynthetic pathway regulatory protein AFLR. Biochimica et Biophysica Acta - Gene Structure Expression 1444: 412-417.

22. Steidl S, Papagiannopoulos P, Litzka O, Andrianopoulos A, Davis MA, et al. (1999) AnCF, the CCAAT binding complex of *Aspergillus nidulans,* contains products of the *hapB, hapC,* and *hapE* genes and is required for activation by the pathway-specific regulatory gene *amdR*. Mol Cell Biol 19: 99-106.

23. Tsitsigiannis DI, Keller NP (2006) Oxylipins act as determinants of natural product biosynthesis and seed colonisation in *Aspergillus nidulans*. Mol Microbiol 59: 882-892.

24. Nahlik K, Dumkow M, Bayram O, Helmstaedt K, Busch S, et al. (2010) The COP9 signalosome mediates transcriptional and metabolic response to hormones, oxidative stress protection and cell wall rearrangement during fungal development. Mol Microbiol 78: 964-979.

25. Helmstaedt K, Schwier EU, Christmann M, Nahlik K, Westermann M, et al. (2011) Recruitment of the inhibitor Cand1 to the cullin substrate adaptor site mediates interaction to the neddylation site. Mol Biol Cell 22: 153-164.

26. Wang C-L, Shim W-B, Shaw BD (2010) *Aspergillus nidulans* striatin (StrA) mediates sexual development and localizes to the endoplasmic reticulum. Fung Genet Biol 47: 789-799.

27. Kim H, Smith JE, Ridenour JB, Woloshuk CP, Bluhm BH (2011) HXK1 regulates carbon catabolism, sporulation, fumonisin B(1) production and pathogenesis in *Fusarium verticillioides*. Microbiology 157: 2658-2669.
