# Supplementary material for: The Genomes of the Fungal Plant Pathogens Cladosporium fulvum and Dothistroma septosporum Reveal Adaptation to Different Hosts and Lifestyles But Also Signatures of Common Ancestry
Source: PLoS Genet. 2012 Nov 29;8(11):e1003088. doi: 10.1371/journal.pgen.1003088 (PMC3510045; doi:10.1371/journal.pgen.1003088)
Supplement: Table S12 — Conditions for Cladosporium fulvum EST libraries. (DOC) [file pgen.1003088.s019.doc]

**Table S12. Conditions for *Cladosporium fulvum* EST libraries**

| **Induction condition** | **EST Library** |
| --- | --- |
| pH.9 | Induced |
| pH.7 | Induced |
| pH.4 | Induced |
| MS Basal medium -Carbon | Induced |
| Mannitol | Induced |
| Nitrogen limitation | Induced |
| Carbon minus | Induced |
| B5 without vitamins & sucrose | Induced |
| Excess of Nitrogen | Induced |
| Excess of Carbon | Induced |
| AF from non-infected tomato (MM) | Induced |
| AF from infected MM-Cf9 tomato | Induced |
| Induction with Alpha-solanine | Induced |
| Heat-shock | Induced |
| Cold-shock | Induced |
| Glucose/Glucose oxidase | Induced |
| 5 mM H_2_O_2_ | Induced |
| Benomyl | Induced |
| Imidazole | Induced |
| Azoxystrobine | Induced |
| Tryfroxystrobin | Induced |
| Cypronazole | Induced |
| **Total** | **22** |
| Progressive Starvation for 5 days | Starved |
| Progressive Starvation for 8 days | Starved |
| Progressive Starvation for 12 days | Starved |
| Progressive starvation for 16 days | Starved |
| Progressive Starvation for 22 days | Starved |
| **Total** | **5** |
| Infected Heinz 4 dpi | *In planta* |
| Infected Heinz 6 dpi | *In planta* |
| Infected Heinz 9 dpi | *In planta* |
| Infected Heinz 12 dpi | *In planta* |
| Infected Heinz 15 dpi | *In planta* |
| **Total** | **5** |
