# Supplementary material for: The Genomes of the Fungal Plant Pathogens Cladosporium fulvum and Dothistroma septosporum Reveal Adaptation to Different Hosts and Lifestyles But Also Signatures of Common Ancestry
Source: PLoS Genet. 2012 Nov 29;8(11):e1003088. doi: 10.1371/journal.pgen.1003088 (PMC3510045; doi:10.1371/journal.pgen.1003088)
Supplement: Table S13 — Conditions for Dothistroma septosporum EST libraries. (DOC) [file pgen.1003088.s020.doc]

**Table S13. Conditions for *Dothistroma septosporum* EST libraries**

| **Induction condition** | **EST Library** |
| --- | --- |
| Pine needle water agar [1] for 8 days | CHAA |
| Water agar for 8 days | CHAA |
| Pine minimal medium broth [1] for 8 days | CHAA |
| Water for 8 days | CHAA |
| **Total** | **4** |
| DSM agar (light) for 8 days | CHAB |
| DSM agar (dark) for 8 days | CHAB |
| V8 juice agar for 8 days | CHAB |
| Potato dextrose broth for 8 days | CHAB |
| DM broth [3] for 8 days | CHAB |
| **Total** | **5** |
| Infected *Pinus radiata* mixed late stage forest sample | *In planta* |
| **Total** | **1** |

**References**

1. Carsolio C (1994) Characterization of Ech-42, a T*richoderma harzianum* endochitinase gene expressed during mycoparasitism. Proc Natl Acad Sci U S A 91: 10903-10907.

2. Bradshaw RE, Ganley RJ, Jones WT, Dyer PS (2000) High levels of dothistromin toxin produced by the forest pathogen *Dothistroma pini.* Mycol Res 104: 325-332.

3. Schwelm A, Barron NJ, Zhang S, Bradshaw RE (2008) Early expression of aflatoxin-like dothistromin genes in the forest pathogen *Dothistroma septosporum*. Mycol Res 112: 138-146.
