# Supplementary material for: Correction: The Genomes of the Fungal Plant Pathogens Cladosporium fulvum and Dothistroma septosporum Reveal Adaptation to Different Hosts and Lifestyles But Also Signatures of Common Ancestry
Source: PLoS Genet. 2015 Dec 22;11(12):e1005775. doi: 10.1371/journal.pgen.1005775 (PMC4700972; doi:10.1371/journal.pgen.1005775)

Figure S4. Growth profile assays for *C. fulvum* (A) and *D. septosporum* (B)

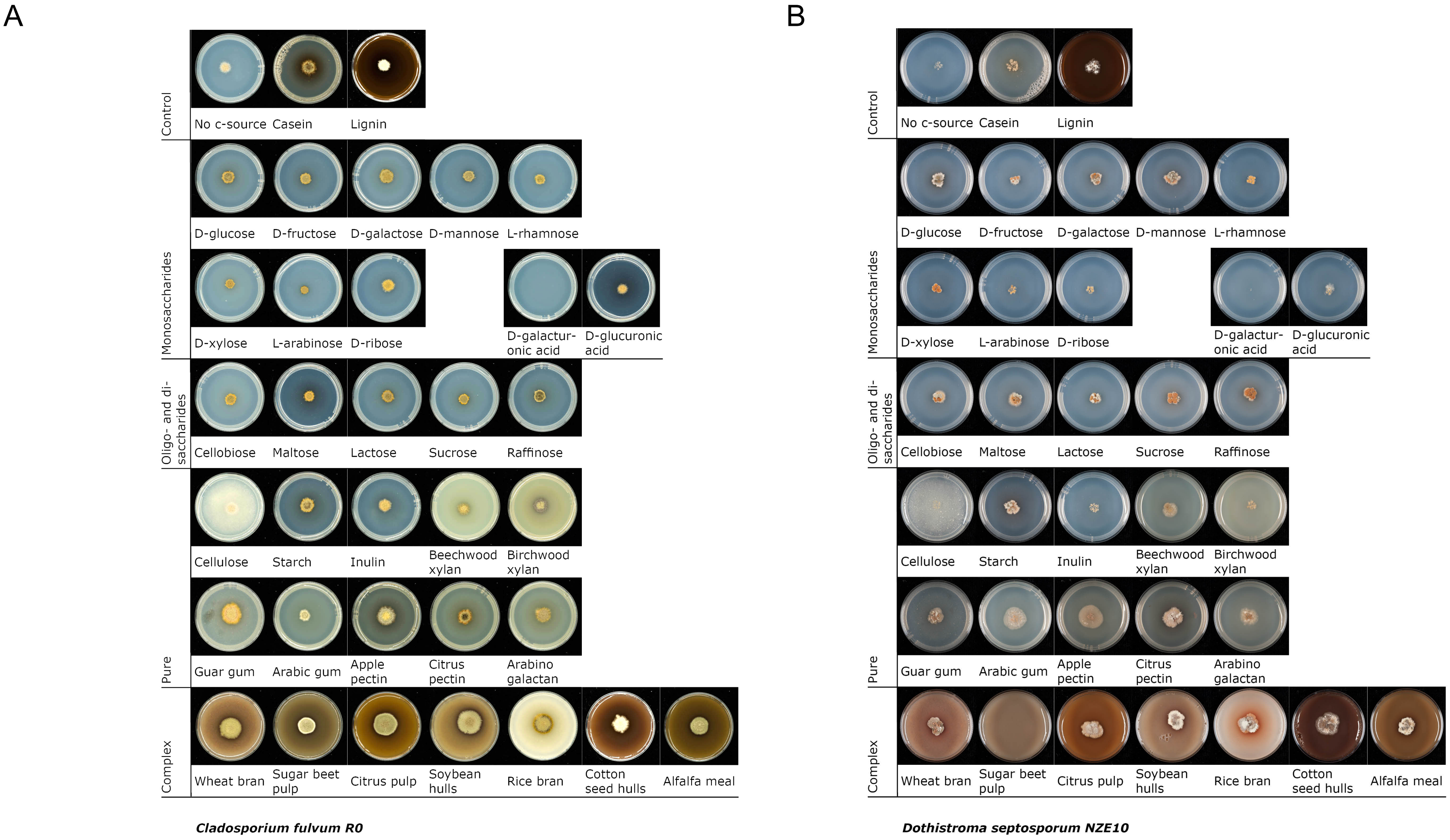

Supplement: S4 Fig — The fungi were grown on 32 solid agar media containing well-defined or complex carbohydrate substrates as detailed at www.fung-growth.org. A) C. fulvum grown for 2 weeks and B) D. septosporum grown for 4 weeks, both in the dark at 22–25°C. (PDF) [file pgen.1005775.s001.pdf]
